# MtrA Response Regulator Controls Cell Division and Cell Wall Metabolism and Affects Susceptibility of Mycobacteria to the First Line Antituberculosis Drugs

**DOI:** 10.3389/fmicb.2018.02839

**Published:** 2018-11-23

**Authors:** Purushotham Gorla, Renata Plocinska, Krishna Sarva, Akash T. Satsangi, Emmanuel Pandeeti, Robert Donnelly, Jaroslaw Dziadek, Malini Rajagopalan, Murty V. Madiraju

**Affiliations:** ^1^Biomedical Research, The University of Texas Health Science Center, Tyler, TX, United States; ^2^Institute of Medical Biology, Polish Academy of Sciences, Lodz, Poland; ^3^Department of Pathology and Laboratory Medicine, Rutgers New Jersey Medical School, Newark, NJ, United States

**Keywords:** *Mycobacterium*, two component regulatory system, MtrA-CHIPSeq, MtrA-cell division, MtrA-response regulator

## Abstract

The biological processes regulated by the essential response regulator MtrA and the growth conditions promoting its activation in *Mycobacterium tuberculosis*, a slow grower and pathogen, are largely unknown. Here, using a gain-of-function mutant, MtrA_Y 102C_, which functions in the absence of the cognate MtrB sensor kinase, we show that the MtrA regulon includes several genes involved in the processes of cell division and cell wall metabolism. The expression of selected MtrA targets and intracellular MtrA levels were compromised under replication arrest induced by genetic manipulation and under stress conditions caused by toxic radicals. The loss of the *mtr*A gene in *M. smegmatis*, a rapid grower and non-pathogen, produced filamentous cells with branches and bulges, indicating defects in cell division and cell shape. The Δ*mtr*A mutant was sensitized to rifampicin and vancomycin and became more resistant to isoniazid, the first line antituberculosis drug. Our data are consistent with the proposal that MtrA controls the optimal cell division, cell wall integrity, and susceptibility to some antimycobacterial drugs.

## Introduction

Tuberculosis remains the leading cause of mortality globally and accounts for, on average, 1.5 million deaths a year. *Mycobacterium tuberculosis*, the causative agent of tuberculosis, is a successful pathogen that employs a host of regulatory networks for its optimal survival upon infection (Cole et al., 1998), one of which is the paired histidine-aspartate two-component regulatory signal transduction system (TCSS) (Hoch, 2000; Stock et al., 2000). This system includes a membrane-bound sensor kinase and a cytosolic response regulator (RR). The sensor kinases respond to specific environmental stimuli, become autophosphorylated and engage in the transphosphorylation of the cognate RR *via* the phosphorelay signal transduction process. The phosphorylated RR then binds to specific sequences in the promoter regions of several target genes and modulates their expression. The *M. tuberculosis* genome contains eleven TCSSs and several orphan kinases and response regulators. Among these, MtrAB and PrrAB are essential for *M. tuberculosis* survival (Zahrt and Deretic, 2000; Haydel et al., 2012). The MtrAB system includes the MtrB (Rv3245c) sensor kinase and the MtrA RR (Rv3246c). Although it was first described nearly 20 years ago, the essential MtrAB system remains poorly characterized.

Earlier studies (Fol et al., 2006) designed to evaluate the roles of MtrA characterized the viability of *M. tuberculosis* strains overproducing phosphorylation-competent wild-type MtrA+ (WT, Rv78), phosphorylation-defective MtrA (MtrA_D56N_, Rv129) and MtrB along with MtrA+ (Rv79) upon infection (the uncommon Rv names Rv78, Rv129, Rv79 are the numbers of *M. tuberculosis* strains from the collection of our laboratory). These studies revealed that the regulation of *M. tuberculosis* proliferation upon infection is in part dependent upon optimal MtrA levels and MtrA phosphorylation (MtrA∼P) and that MtrB activity is likely involved in regulating MtrA∼P (Fol et al., 2006). These data also indicated that MtrA∼P level is modulated during intracellular growth. Other studies identified that the promoters (*P*) of the cell wall hydrolase *rip*A (*rv1477*), the cell wall mycolyl transferase *fbp*B (*rv1886c*) and the replication initiator *dna*A (*rv0001*) are MtrA targets (Rajagopalan et al., 2010; Plocinska et al., 2012). The role of MtrAB two component system was also studied in *M. smegmatis* cells, a rapid grower and non-pathogen. The MtrB (MSMEG_1875) sensor kinase is not essential in *M. smegmatis*, and *mt*rB disruption compromised MtrA-target expression and cell division (Plocinska et al., 2012). The *mtrB* KO phenotype was reversed by either the production of an intact MtrB or overproduction of MtrA (MSMEG_1874) carrying Y102C mutation which favors MtrA phosphorylation and/or DNA binding even in the absence of MtrB (Plocinska et al., 2012). The overproduction of MtrA_Y 102C_ in MtrB KO mutant caused not only the reversal of the Δ*mtrB* phenotype but also increased the expression levels of MtrA targets (*dnaA, ripA fbpB, ftsI* and *wag31*) (Plocinska et al., 2012). Recent studies show MtrB interacts with FtsI (Rv2163c, penicillin-binding protein 3 with transpeptidase activity) and Wag31 (Rv2145c, the cell wall synthesis protein). The expression levels of *mtrA*, along with MtrA targets *dnaA, fbpB* and *ripA* were decreased under FtsI depletion conditions. FtsI, in contrast to Wag31, functions as a positive modulator of MtrB activation and MtrA regulon expression (Plocinska et al., 2014). It was also shown that phosphorylation defective MtrA carrying both the D56N and Y102C mutations was able to bind its DNA targets and reverse, at least partially, *mtrB* KO phenotypes in *M. smegmatis* indicating that phosphorylation is not required for the function of MtrA_Y 102C_ (Satsangi et al., 2013). More recently it was reported that the access of the wild type MtrA to origin of replication (*oriC*) in tubercle bacilli depends on its phosphorylation. The abundant *oriC* binding by phosphorylated MtrA as well as MtrA_Y 102C_ reduced the *dnaN (rv0002)* and *dnaA* expression, interfered with replication synchrony, and compromised cell division (Purushotham et al., 2015). Moreover, the *in vitro* study has shown that phosphorylated and non-phosphorylated MtrA_Y 102C_ binds *oriC* with similar affinity (Purushotham et al., 2015).

The high-throughput sequencing (CHIP-seq) analysis was also applied to identify the MtrA targets in two distinct studies (Minch et al., 2015; Chatterjee et al., 2018).

Although the above studies identified a handful of MtrA targets and connected MtrA activity to the cell cycle, several questions remain. For example, how does MtrA activity impact the cell division and cell wall metabolism processes? Which of the genes involved in these such important processes are under MtrA∼P control, and what are the members of the MtrA∼P regulon? Here, we performed chromatin immunoprecipitation of MtrA_Y 102C_ phosphorylation competent protein followed by high-throughput sequencing under active and stationary-phase growth conditions to elucidate a comprehensive MtrA∼P regulon. Our CHIP-seq analysis for MtrA_Y 102C_ a gain-of-function protein that binds to its targets independent of phosphorylation (Satsangi et al., 2013), identified and moreover expanded new targets, not being identified by previously published CHIP-seq analysis for MtrA, performed by Minch et al. (2015) and Chatterjee et al. (2018). Additionally, we evaluated the growth conditions where the MtrA system is active and, finally, created and characterized a *M. smegmatis* Δ*mtr*A mutant strain. Based on transposon mutagenesis studies (TRASH), *mtrA* is an essential gene for growth and survival of *M. tuberculosis* (Sassetti et al., 2003). Despite numerous attempts we were not able to construct Δ*mtrA* mutant in *M. tuberculosis* cells. These studies revealed that MtrA is a key regulator of optimal cell wall integrity and cell division in replicating cells and its depletion affects susceptibility of *M. smegmatis* cells to the first line antituberculosis drugs.

## Materials and Methods

### Bacterial Strains and Proteins

The oligonucleotide primers used in the study are listed in Supplementary Table S1. The description of strains and plasmids is included in Supplementary Table S2. *M. tuberculosis* (H37Rv) and *M. smegmatis* (mc^2^155) strains were grown in Middlebrook 7H9 media supplemented with OADC (oleic acid-albumin-dextrose-catalase) and ADC supplements, respectively. Recombinant maltose binding protein fusions of EnvZ, MtrA, and MtrA_Y 102C_ were produced in *Escherichia coli* as described (Plocinska et al., 2012). Bacterial growth was assayed by measuring changes in absorbance at 600 nm, and viability was determined by assaying colony-forming units per mL. For some experiments, in order to induce stress conditions, the actively growing *M. tuberculosis* cells were exposed to 0.2% SDS or 100 μM DETA-NO for 2 or 16 h, respectively. Next, cells were harvested and RNA was isolated according to the protocol described previously (Fol et al., 2006).

### Cloning and Construction of Plasmids

The plasmids and oligonucleotide primers used in this study are listed in Supplementary Tables S1, S2. The coding regions of various genes were PCR amplified with Phusion DNA polymerase (New England BioLabs Inc., Ipswich, MA, United States) and cloned into various plasmids using standard molecular biology techniques. Cloned regions were confirmed by sequencing. The PCR products corresponding to ChIP-seq peaks were amplified using primer pairs (Supplementary Table S1) and cloned as HindIII-SacI fragments into the plasmid pUC57. FAM-labeled primers targeting the vector sequence flanking the insert were used to generate DNA probes for EMSA.

### Phenotype Analysis

Microscopy: Actively growing *M. tuberculosis* and *M. smegmatis* cells were visualized by bright-field and fluorescent microscopy as described (Plocinski et al., 2012). All *M. tuberculosis* cells were fixed in 4% paraformaldehyde prior to visualization. To evaluate sensitivity to antibiotics, actively growing *M. smegmatis* cultures were diluted to an OD600 of 0.05 for 6 h, and approximately 1 × 10^5^ cells were spread with sterile cotton-tipped swabs. Next, *E*-test strips (ampicillin, vancomycin, rifampicin, or isoniazid) were placed on the culture plates and incubated at 37°C for 4 days prior to recording as described (Plocinski et al., 2013; Plocinska et al., 2014). The MIC values were determined following the E-test manufacturer’s recommendations.

### Creation of *M. smegmatis mtrA* Mutant

A two-step recombination protocol was used to delete the *mtr*A gene of *M. smegmatis*. First, the 5′ end of *mtrA* (125 bp) and the upstream region were PCR amplified using the primers MtrAsmegGR1ScaI and MtrAsmegGR2HindIII and cloned into the p2NIL vector to create pDR45 (Supplementary Tables S1, S2). Next, a 1530-bp fragment including 365 bp from the 3′ end of *mtrA* and the downstream region was PCR amplified with the primers MtrAsmegGR3HindIII and MtrAsmegGR4PacI and cloned into pDR45 to create pDR47. An 850-bp gentamicin cassette was then cloned into the HindIII site of pDR47 to create pDR49. Finally, a 6000-bp cassette containing the *lac*Z and *sacB* genes from pGOAL17 was inserted in pDR49 to create the final suicidal recombination vector pDR51. The plasmid DNA of pDR51 was pre-treated with UV light and electroporated into *M. smegmatis* competent cells. Prior to double-cross-over (DCO) screening, the plasmid pDR54 expressing the *M. tuberculosis mtrA* gene under a tetracycline-inducible promoter was transformed into one representative SCO strain. White DCO colonies that were resistant to sucrose and sensitive to kanamycin were further confirmed by PCR and Southern blotting approaches (GE Healthcare). Finally, the pDR54 plasmid was swapped out with the pMV306K vector as described (Chauhan et al., 2006b).

### Verification of the Essentiality of *mtrB* in *M. tuberculosis*

A two-step recombination protocol was used to verify the essentiality of *mtrB* in tubercle bacilli. The upstream region of *M. tuberculosis mtrB* gene (2145 bp) was cloned into suicidal recombination p2Nil vector, followed by the downstream fragment of *mtrB* sequence (1566 bp), creating pDR56. Next, gentamicin cassette was inserted into HindIII site. Finally, the screening cassette from pGOAL17 vector was cloned and resulting suicide delivery vector pDR58 was used to engineer the direct *mtrB* mutant as described above and by Dadura et al. (2017). Next, the complementation plasmid expressing *mtrB* gene under acetamide promoter (pRD102) (Plocinska et al., 2012) was introduced into representative SCO strain, in order to process for DCO screening. Next, the pRD102 was swapped with pKS4 vector expressing *mtrB*-*gfp* and kanamycin resistance cassette (Plocinska et al., 2012). Finally, the pKS4 vector was swapped with pDS4, expressing *mtrA*_Y 102C_ and hygromycin cassette. The genotypes of Δ*mtrB*::*mtrA*_Y 102C_ strain was confirmed by Southern blot hybridization using probe to *mtrB* gene and following manufacturer instructions (GE Healthcare). Plasmids and primers used for PCR amplification are listed in Supplementary Tables S1, S2.

### RNA Extraction and Quantitative Real-Time PCR

Extraction of total RNA and quantitative real-time PCR were performed in a BioRad iCycler iQ^TM^ Real-Time PCR detection system using FAM fluorophore-labeled 2X iQ SYBR Supermix (BioRad, Cat# 1708880) as described (Maloney et al., 2009; Rajagopalan et al., 2010). The threshold cycle (*Ct*) value of each gene of interest was normalized to the *Ct* value of 16S rRNA, and the fold expression was calculated [fold change = 2^-Δ(Δ^*^Ct^*^)^]. Expression data were obtained from an average of three independent RNA preparations, and each gene of interest was investigated in triplicate. Fold differences of 2 or more were considered significant. Primers used for qRT-PCR are listed in Supplementary Table S1.

### Electrophoretic Mobility Shift Assay (EMSA)

Electrophoretic mobility shift assay was carried out to detect MtrA binding to FITC-labeled promoter *PrpfB* and FAM-labeled promoters: *PripA, PsigD, PoxyS, Pwag31, PbetP, Prv3887c, PwhiB3, PdacB1* or MtrA_Y 102C_ binding to FAM-labeled promoters: *PbetP, Prv2525* and *PdacB1* as described (Plocinska et al., 2012; Satsangi et al., 2013; Purushotham et al., 2015). The 200 bp upstream regions of chosen targets were amplified using primers listed in Supplementary Table S1. MtrA/MtrAY102C were phosphorylated by EnvZ (Al Zayer et al., 2011) and incubated at 1, 2, 4, 6, 10 μM concentration with 200 fmols promoter DNA’s in buffer containing 50 mM Tris-HCl pH 7.5, 50 mM NaCl, 10 mM MgCl_2_, 10 mM CaCl_2_. Next, 20 pmoles poly dI/dC and shared salmon sperm DNA (1 μg) were added, reactions were incubated at 37°C for 15 min and resolved in 5% polyacrylamide gels. The DNA-protein complexes were visualized using Molecular Imager Fx (BioRad).

### Chromatin Immunoprecipitation (ChIP)

Detailed methodology for ChIP-seq sample preparation, processing of libraries, SOLiD sequencing, data analyses and motif prediction are presented in the Supplementary Data. Briefly, *M. tuberculosis* cultures producing MtrA_Y 102C_ growing for 3 (exponential growth) and 8 (stationary growth) days were cross-linked by fixing in 1% formaldehyde and processed for ChIP analysis with anti-MtrA antibodies essentially as described (Fol et al., 2006; Rajagopalan et al., 2010). The protein-DNA complexes obtained following incubation of sheared lysates with anti-MtrA antibodies were recovered using ImmunoPure immobilized Protein G agarose beads (Thermo Fisher Scientific, Rockford, IL, United States), and the cross-links were reversed by incubation at 65°C for 16 h. DNA samples were purified using DNAzol, resuspended in 50 μl of TE buffer, processed and used either for individual target evaluation (Fol et al., 2006; Rajagopalan et al., 2010; Plocinska et al., 2012) or for SOLiD library construction (detailed in the Supplementary Data).

## Results

### The *mtrB* of Tubercle Bacilli Is Not Essential in the MtrA_Y 102C_ Genetic Background

The high density transposon mutagenesis (TRASH) suggested that in distinction to *M. smegmatis* the membrane-bound sensor kinase MtrB responsible for the phosphorylation of the response regulator MtrA is essential in *M. tuberculosis* (Sassetti et al., 2003). Since the MtrA_Y 102C_ is believed to be phosphorylation competent and functions in the absence of MtrB, we tested whether *mtrB* can be inactivated in *M. tuberculosis* complemented with *mtrA*_Y 102C._ The gene replacement protocol (Parish and Stoker, 2000; Dziadek et al., 2002b) was used to engineer *M. tuberculosis* mutants carrying both wild type and Δ*mtrB* genes (SCO) which were further processed for homologous recombination to select double cross-over mutants (DCO) carrying either wild type or mutated *mtrB*. We have analyzed over 50 DCO mutants by using PCR screening and in all cases only wild type *mtrB* gene was detected. Multiple attempts to replace complementing vector pRD102 with empty pMV306 vector confirmed essentiality of MtrB protein in *M. tuberculosis* cells. On the other hand the native *mtrB* was replaced with an inactivated copy (Δ*mtrB::gm^R^*) when *mtrB-gfp* or *mtrA*_Y 102C_ were introduced with integration vectors into an *attB* site. The genotypes of the merodiploid strains (Δ*mtrB::gm^R^-attB::mtrB-gfp* or -*attB::mtrA*_Y 102C_) were confirmed by Southern hybridization (Supplementary Figure S1), confirming that MtrA_Y 102C_ is able to replace at least partially the function of phosphorylated MtrA.

### The Replacement of Cys With Tyr in Position 102 of MtrA Results in Signal Independent Phosphorylation of MtrA

**FIGURE 1:**
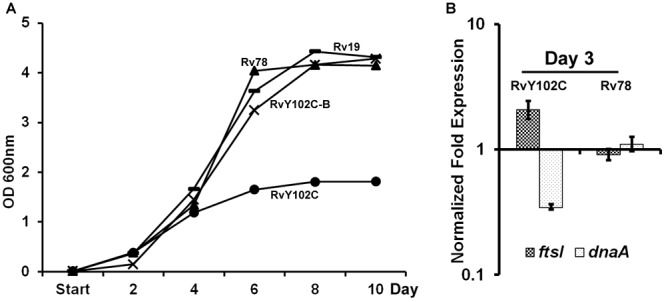
Characterization of RvY102C. **(A)** Growth of *M. tuberculosis* Rv19, Rv78, RvY102C, RvY102C-B carrying empty vector, *mtrA, mtrA*_Y 102C_ and *mtrA*_Y 102C_-*mtrB* plasmids, respectively (see Supplementary Table S2). **(B)** Expression of *dna*A and *fts*I in RvY102C (normalized to 16S rRNA) determined by qRT-PCR. Data shown are fold expression in Rv78 and RvY102C relative to Rv19. The total RNA was isolated from three independent biological replicates and the data are represented as the means ± standard errors from three independent experiments.

Based on the crystal structure of MtrA indicating tyrosine at position 102 is at the interdomain interface (Friedland et al., 2007), we have previously engineered *M. tuberculosis* strain producing MtrA_Y 102C_ (RvY102C), which contains cysteine in place of the tyrosine located at the interface of the regulatory and DNA-binding domains (Plocinska et al., 2012). Because MtrA_Y 102C_ is phosphorylation competent and functions in the absence of the cognate sensor kinase MtrB (Plocinska et al., 2012; Satsangi et al., 2013), we reasoned that MtrA_Y 102C_ would be activated in the absence of the specific exogenous signals that are otherwise necessary for the MtrB autophosphorylation and transphosphorylation activities. The RvY102C mutant initially grew similarly to the WT and MtrA overproducing strain (Rv78) during exponential phase, however, reached the stationary phase much faster (OD600 value for Rv19 – 4,315; Rv78 – 4,144; RvY102C-B – 4,28; RvY102C – 1,812) (Figure 1A). Similar results were also obtained when the plasmid carrying MtrA_Y 102C_ was transformed into other *M. tuberculosis* strain CSU#1, suggesting that the expression of *mtrA*_Y 102C_ is enough to affect the growth in two different genetic background strains (Supplementary Figure S2). Overexpression of *mtr*B along with *mtr*A_Y 102C_ reversed the observed growth defect associated with MtrA_Y 102C_ overproduction (Figure 1A, compare RvY102C-B with RvY102C), corroborating the previous data that MtrB modulates MtrA∼P potential (Fol et al., 2006). Independently, we evaluated the expression levels of the MtrA targets *dna*A (Fol et al., 2006) and *fts*I [see below, also (Plocinska et al., 2012; Plocinska et al., 2014) in the RvY102C background relative to WT]. Expression of *dna*A was decreased, whereas that of the *fts*I was increased in RvY102C relative to WT (Figure 1B). No significant differences in the *dna*A and *fts*I expression were noted in Rv78 (Figure 1B).

### MtrA Regulon

**FIGURE 2:**
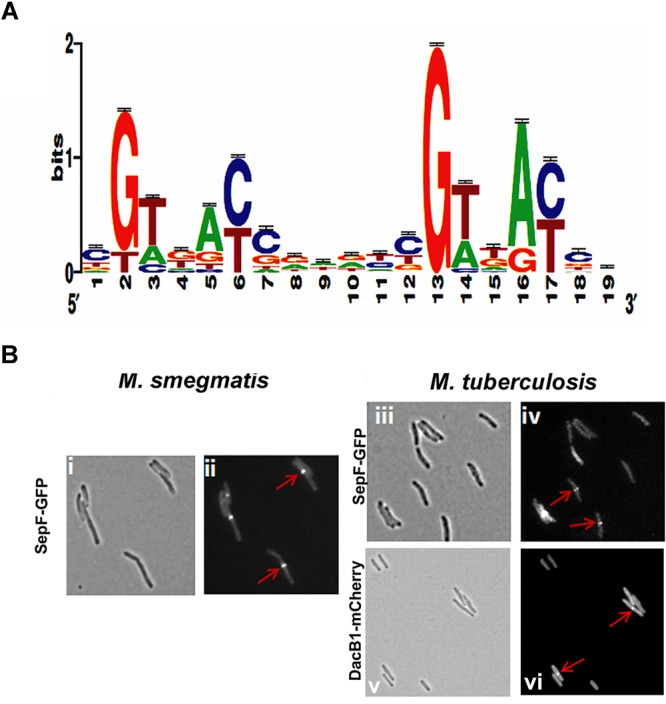
MtrA motif and validation of DacB1 and SepF septasomal association: **(A)** MtrA logo determined by analyzing the MtrA regulon ChIP-Seq peaks. The *p*-value of the deduced motif is 2.6e-123, and the motif is a direct repeat of 9 nucleotides with a spacing of 2 nucleotides, starting with either G or T and possessing a sequence significantly similar to the previously defined MtrA-motif based on DNase I footprinting of *ori*C and *Pfbp*B targets (Rajagopalan et al., 2010), as well as to that of the MtrA-motifs of *C. glutamicum* and TB database (Brocker et al., 2011; Galagan et al., 2013), except for differences with respect to the weightings of the individual bases. **(B)** Localization of DacB1-mCherry in *M. tuberculosis* and SepF-GFP in *M. smegmatis* and *M. tuberculosis*. Bright-field **(i,iii,v)** and respective fluorescence **(ii,iv,vi)** images of and *M. tuberculosis* producing DacB1-mCherry and *M. smegmatis* and *M. tuberculosis* producing SepF-GFP are shown. Arrows denote septal localization.

We reasoned from the above data that the RvY102C background would enable us to elucidate a comprehensive MtrA regulon in the absence of exogenous MtrAB system activation signals. Considering the growth characteristics of RvY102C, samples collected from exponential phase (day 3) and stationary phase (day 8) cultures were processed for ChIP-seq studies to define a full spectrum of the MtrA regulon based on the two different growth states. CHIP-seq analysis were performed using anti-MtrA polyclonal antibodies to detect all MtrA, MtrA-P as well as MtrA_Y 102C_. We identified a total of 216 ChIP-seq peaks that were present in duplicate samples from day 3, extracted the sequences corresponding to ChIP peaks, analyzed the data using the MEME software, and defined the MtrA motif using a Web logo tool, which consists of the a degenerated direct repeat of c-G-T/A-n-A-C/T-c, separated by four nucleobases (Figure 2A). This motif, which begins with either G or T, has a significant sequence similarity to the previously defined MtrA motif based on DNaseI footprinting of *ori*C and *Pfbp*B (Rajagopalan et al., 2010). However, differences with respect to the weights of the individual bases were noted. The predicted MtrA motif was then used to search for possible matches in the day 8 ChIP-seq peaks with relatively high scores. Using this approach we identified a total of 278 target sites, of which 93 were common to day 3 and day 8, whereas 62 were unique to day 8 only. A comprehensive list of MtrA regulon genes based on both sets of data was generated (Supplementary Table S3 and Supplementary Figure S3A, select peaks at both growth states) and classified into families based on Tuberculist (Supplementary Figure S3B). As could be expected, this list includes new targets not reported previously (Galagan et al., 2013). The identification of the confirmed MtrA targets *Pfbp*B and *Prip*A and the absence of the non-targets *Pfts*Z (*rv2150c*) and *Ppfk*B (*rv2029c*) (Rajagopalan et al., 2010; Plocinska et al., 2012) combined with the presence of an MtrA motif in every designated ChIP-seq peak increased the degree of confidence in our analysis. MtrA binding to select targets *PsigD* (*rv3414c*), *PoxyS* (*rv0117*), *Pwag31, PbetP* (rv0917), *Prv3887, PwhiB3* (*rv3416*) and MtrA_Y 102C_ binding to *PbetP, Prv2526* was further validated by EMSA (Supplementary Figure S4). The previously reported MtrA binding to *PrpfB* (*rv1009*) (Rajagopalan et al., 2010) and *PripA* (Plocinska et al., 2012) was a positive control in our experiment. To investigate the possibility of protein-DNA complexes we incubated recombinant MtrA and MtrA_Y 102C_ (Plocinska et al., 2012) with FAM-labeled DNA. We identified complexes of MtrA-P as well as MtrA in the absence of phosphorylation with *PrpfB, PripA, Prv3887*. In case of *PsigD* and *PoxyS* we observed better binding to MtrA-P than to MtrA. Only MtrA-P retarded *Pwag31* and *PwhiB3*. Both, MtrA and MtrA-P did not show binding to *PbetP*.

### Cell Division and Cell Wall Metabolism Genes Within MtrA Regulon

Cell division involves the midcell FtsZ assembly (Z-ring formation), followed by successive localization of multiple proteins to a Z-ring, which is also referred to as divisome assembly; septal cell wall synthesis; and cell separation events (Adams and Errington, 2009). The cell division process is interconnected and coordinated with cell wall elongation, which in mycobacteria occurs from the poles (Aldridge et al., 2012; Santi et al., 2013). We noted that the MtrA regulon includes a number of cell division and cell wall metabolism genes, including peptidoglycan hydrolase (*rip*A), mycolyltransferases (*fbp*B, *fbp*C (*rv0129c*)), penicillin-binding protein involved in peptidoglycan biosynthesis (*fts*I, *dac*B1 (*rv3330*), FtsZ-interacting protein (*sep*F) (*rv2147c*), essential DivIVa family cell division protein (*wag*31) and resuscitation-promoting factor *rpf* (A-E) (*rv0867c, rv1009, rv1884c, rv2398c, rv2450c*, respectively) (Supplementary Table S3). The identification of *dac*B1 gene by ChIP was positively validated by *in vitro* study using EMSA assay (Supplementary Figures S5A,Bi,ii). The increasing concentrations of MtrA or MtrA_Y 102C_, phosphorylated by EnvZ and non-phosphorylated were incubated with FAM-labeled promoter of *dacB1*. EMSA studies showed better binding of MtrA-P than MtrA to *PdacB1* (Supplementary Figure S5Bi) and similar binding of MtrA_Y 102C_ with and without phosphorylation (Supplementary Figure S5Bii).

With the exception of *dac*B1, the involvement of the above target in the septum synthesis and cell wall expansion processes in mycobacteria have been established (Belisle et al., 1997; Nguyen et al., 2007; Plocinska et al., 2012; Plocinski et al., 2012; Gupta et al., 2015). In *B. subtilis*, SepF interacts with FtsZ, promotes FtsZ anchoring to membranes and supports the late steps of septum synthesis (Hamoen et al., 2006; Sauvage et al., 2008). In mycobacteria SepF protein also interacts with FtsZ and regulates the cell division process (Gupta et al., 2015). Through fluorescent protein-fusion experiments, we were able to confirm that *M. smegmatis* and *M. tuberculosis* SepF and *M. tuberculosis* DacB1 are also septasomal components (Figure 2B). We found the SepF-GFP localized to the mid-cell in 42% of *M. smegmatis* cells (*n* = 100) and in 36.6% of *M. tuberculosis* cells (*n* = 101). DacB1-mCherry localization to the septum was counted in 14.5% of *M. tuberculosis* cells (*n* = 103). The GFP expressed alone within the control cells revealed dispersed fluorescence as published (Dziadek et al., 2002a). Thus, the ability of MtrA to target a wide array of cell division and cell wall expansion proteins implies that its activity is crucial for those processes.

### The Stress Conditions Down-Regulate MtrA Expression and Its Regulon

**FIGURE 3:**
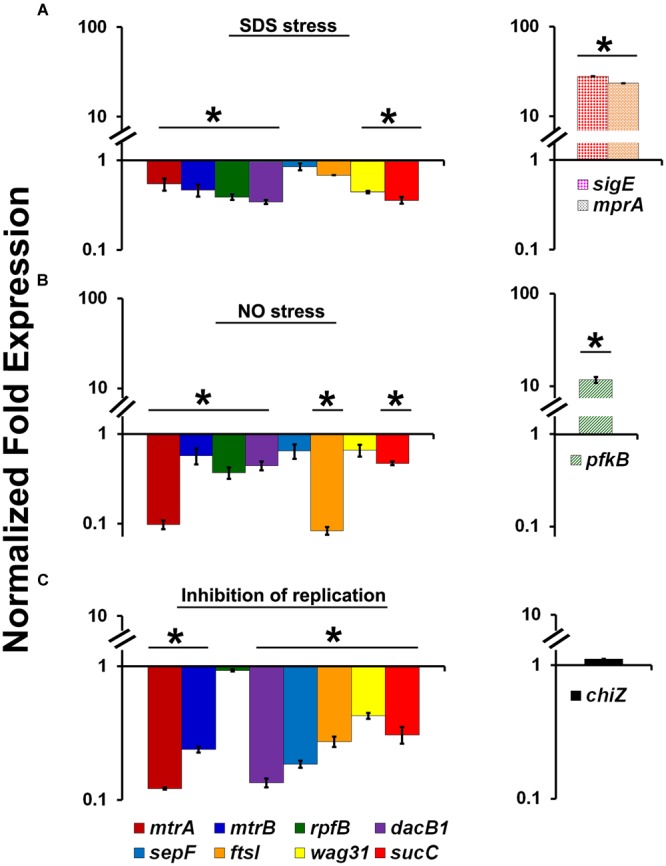
Expression profiles of select MtrA target genes (*mtrA, mtrB, rpfB, dacB1, sepF, ftsI, wag31*, and *sucC*) under stress and replication-arrest conditions. **(A)** Total RNA was extracted from actively growing *M. tuberculosis* cultures exposed to 0.2% SDS for 2 h and the expression levels were analyzed by qRT-PCR as described in Section “Materials and Methods.” **(B)** Total RNA was extracted from actively growing *M. tuberculosis* cultures exposed to 100 μM DETA-NO for 16 h and qRT-PCR was performed to analyze gene expression profiles. **(C)** For evaluating the MtrA-target expression under replication inhibition conditions, *M. tuberculosis dna*A^Ts^ cultures growing at permissive 37°C were shifted to 30°C and incubated for 30 h (non-permissive temperature). RNA samples prepared from cultures growing at both permissive and non-permissive temperatures were processed for evaluation of the target gene expression using qRT-PCR approach. The expression levels of selected genes at non-permissive temperature referred “Inhibition of replication” condition, relative to permissive temperature (37°C) are present on the graph. The qRT-PCR data are normalized to 16S rRNA levels. Mean values and standard deviations from three independent experiments are shown. Expression levels more than twofold was considered as significant upregulation and less than 0.5 fold as the significant reduction in gene expression of each target. Significant expression of each target or group of targets was marked with ‘^∗^’. The targets showed without ‘^∗^’ indicates no significant change in expression. The statistical analyses were performed using the Student’s *t*-test.

The ability of MtrA to affect the cell division and cell wall metabolism processes suggests that its activity is relevant and possibly necessary in replicating cells. To test this possibility, we evaluated MtrA target gene expression under selected growth conditions that compromise proliferation, such as cell envelope stress, DNA and protein damage, and DNA replication inhibition. It is anticipated that *M. tuberculosis* encounters these types of stresses in the hostile host environment upon infection (Smith, 2003). Accordingly, we exposed actively growing *M. tuberculosis* to SDS and DETA-NO to induce envelope/membrane stress and nitric oxide (NO) stress, respectively, as described (Chauhan et al., 2006a; He et al., 2006; Pang et al., 2007). SDS stress induces the *mpr*A (*rv0981*, regulator of TCSS MprAB) and *sig*E (*rv1221*) genes and their associated regulons; both MprA and SigE are regulated by each other under SDS stress (He et al., 2006; Pang et al., 2007). As *sig*E is also a member of the MtrA regulon (this study), we explored the possibility of whether SDS exposure also induces the expression of other MtrA-regulon members. SDS stress led to an induction of *mpr*A and *sig*E expression as expected (Figure 3A), whereas the expression of *mtr*A along with *mtr*B and most of the MtrA targets was decreased by 2- to 3-fold (Figure 3A). Similar to the situation with SDS stress, DETA-NO exposure also compromised the expression of *mtr*A and its targets while the expression of *pfk*B, a member of the DosRS regulon and a marker for NO stress, was elevated (Figure 3B). It was previously reported, MtrA-target expression was also compromised in *M. smegmatis* cells upon exposure to mitomycin C, an agent that damages DNA and interferes with DNA replication (Plocinska et al., 2012). The expression of *chi*Z (*rv2719c*), a marker for DNA damage, was elevated in both *M. tuberculosis* (Chauhan et al., 2006a) and *M. smegmatis* cells exposed to mitomycin C (Plocinska et al., 2012). It is not readily apparent why the expression of some targets did not change under the above stress conditions; either their expression continues under limiting MtrA∼P conditions or is complex and may perhaps also be impacted by other yet-to-be-identified regulator(s). Together, these data indicate that the replication arrest down-regulate MtrA expression and MtrA regulon. To verify this hypothesis we used temperature-sensitive *M. tuberculosis* mutant expressing DnaA protein, referred to as *dnaA*^Ts^, that is defective for binding to ATP at 30°C. This strain is cold sensitive for replication initiation at 30°C; however, the non-permissive temperature does not affect the ongoing rounds of replication. Upon a shift to the permissive temperature of 37°C, the mutant resumes replication initiation and DNA synthesis in a synchronous manner (Nair et al., 2009). Accordingly, RNA samples collected from the *dna*A^Ts^ mutant growing at a permissive temperature (37°C) and those incubated at a non-permissive temperature (30°C) for 30 h were processed for *mtrA, mtrB, rpfB, dacB1, sepF, ftsI, wag31, sucC* and *chiZ* expression analysis (Please see the schematic of experimental plan in Supplementary Figure S6). Compromised expression of the MtrA targets, with the exception of *rpf*B and *chiZ*, at non-permissive temperatures relative to 37°C was noted (Figure 3C). Expression levels were, however, restored upon a shift to 37°C (Purushotham et al., 2015).

**FIGURE 4:**
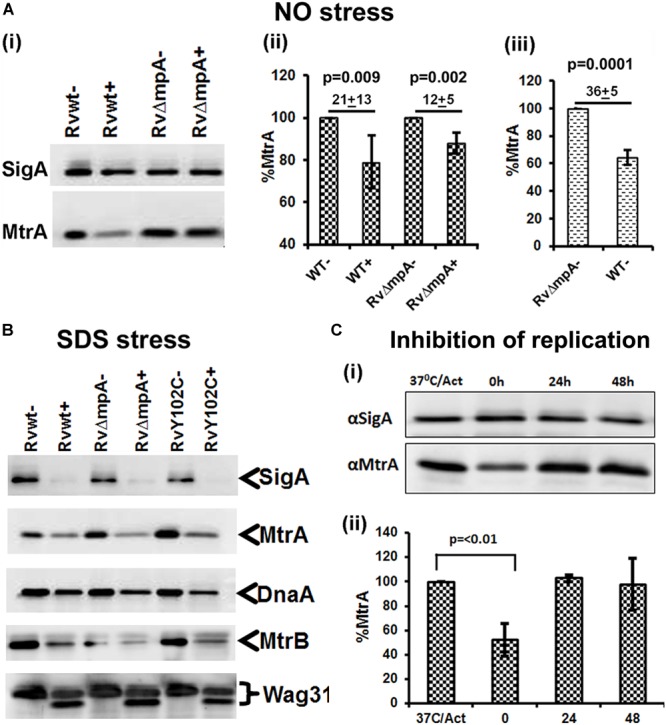
MtrA levels decrease under stress and replication-arrest. Two micrograms of total protein lysates from WT and RvΔ*mpa* strains exposed to DETA-NO **(A)**, SDS **(B)** and isolated from temperature sensitive *M. tuberculosis* dnaA^Ts^ mutant strain (Nair et al., 2009) **(C)** were resolved by SDS-PAGE, transferred to PVDF membranes and immunoblotted with α-MtrA, α-DnaA or α-SigA and quantitated as described (Fol et al., 2006; Rajagopalan et al., 2010). In all cases, untreated cultures are marked with ‘–‘ and treated with ‘+’. **(A)** Includes 3 sub-panels– **(i)**: immunoblots showing SigA and MtrA proteins. **(ii)** MtrA and SigA specific bands were quantified using the volumetric analysis tool of the QuantityOne software, the MtrA/SigA ratio was calculated and data presented as percent MtrA level in WT and RvΔ*mpa* mutant strains treated with DATA-NO, treating the MtrA level in WT and mutant strains not treated with DATA-NO as 100%. **(iii)** Percent of MtrA level in WT strain in comparison to MtrA level in Rv*Δmpa* strain, treated as 100%. **(B)** Immunoblots of SigA, MtrA, DnaA, MtrB, and Wag31 levels in *M. tuberculosis* strains. **(C)** Total protein lysates were isolated from *dnaA*^Ts^ cells collected at four different time points of the synchronization plan: 37°C/Act – actively growing cells at 37°C; 0 – cells shifted to 30°C and grown for 30 h; 24 – cells shifted to 37°C and grown for 24 h; 48 – cells shifted to 37°C and grown for 48 h. The schematic of typical *dnaA*^Ts^ synchronization plan is shown on Supplementary Figure S6 (i) immunoblots showing MtrA and SigA proteins. **(ii)** The MtrA/SigA ratio was calculated as described above. The bar graph shows the percent of MtrA protein level at indicated time period: 0, 24, 48 h in synchronized cells, treating the MtrA level in actively growing cells at 37°C (37°C/Act) as 100%. All data shown are averages ± standard error from three independent experiments. The statistical analyses were performed using the Student’s *t*-test.

We asked whether the compromised expression of the MtrA targets under stress and a replication-arrested state is, in part, due to altered intracellular MtrA levels. Immunoblot analysis of MtrA levels in *M. tuberculosis* cells, relative to the housekeeping protein SigA, under NO stress revealed a significant reduction in MtrA levels (21 ± 13%) comparing to MtrA levels in wild-type cells, without NO stress (100%) (Figures 4Ai,ii). The NO-damaged proteins are believed to be processed for degradation *via* the Mpa-dependent proteasome pathway (Darwin et al., 2003), and MtrA is a predicted proteasome target (Festa et al., 2010). We found that MtrA was also degraded, albeit modestly, under NO stress in the *mpa* mutant (12 ± 5%) in comparison to MtrA levels (100%) in *mpa* mutant without NO stress (Figure 4Aii). Immunoblot analysis of the MtrA levels in the WT background in the absence of stress revealed 36 ± 5% reduction, assuming the MtrA levels in the *mpa* mutant as 100% (Figure 4Aiii). MtrA was also degraded in the SDS-exposed cultures, although SigA, DnaA, MtrB, and Wag31 (Figure 4B), were also degraded under these conditions; hence, the data are not normalized. We also found that MtrA levels were decreased by twofold in *dnaA^Ts^* mutant at the non-permissive temperature of 30°C (time point marked as “0” on Figure 4C), but the levels were restored to near WT levels at 24 and 48 h time point upon a shift to permissive conditions (37°C) (time points marked as “24” and “48” on Figure 4C). The diagram representing synchronization plan is shown on Supplementary Figure S6. Together, our results indicate that intracellular MtrA is subject to degradation under stress and in non-replicating cells and that the Mpa proteasome activity is likely important for maintaining MtrA levels.

**FIGURE 5:**
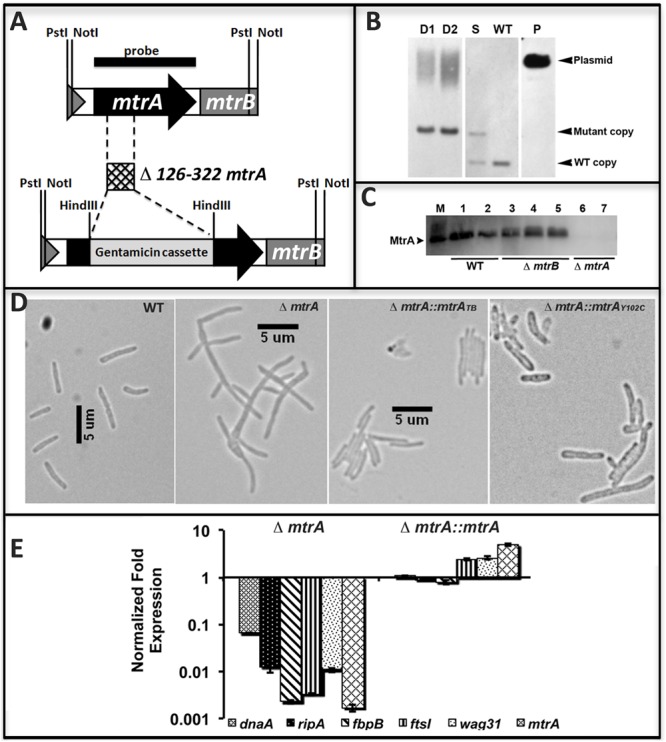
Construction and characterization of *M. smegmatis mtrA* mutant. **(A)** Schematic of *mtrAB* region showing the deletion of nucleotides 126–322 followed by insertion of gentamicin cassette in the *mtrA* coding region. **(B)** Southern blot confirming the deletion of *mtr*A. Genomic DNA was isolated from WT *M. smegmatis*, one single-crossover (S) and two double-crossovers (D1 and D2), digested with *Not*I, transferred to Hybond-N+ membrane (GE Healthcare) and probed with *mtr*A fragment (black bar in **A**). Bands corresponding to the WT (1306-bp) and mutant (1921-bp) copy were seen in single crossover and those corresponding to WT only or mutant only copy were seen in WT and double crossover strains, respectively. Size of mutant band includes 851-bp gentamicin cassette. **(C)** The loss of MtrA in Δ*mtrA* strain was confirmed by immunoblotting with MtrA antibodies. **(D)** Loss of MtrA leads to filamentation and cell shape defects. Exponential cultures of *M. smegmatis* WT, Δ*mtrA*, Δ*mtrA Pami::mtrA*, and Δ*mtrA Pami::mtrA_Y 102C_* were examined by bright field microscopy and imaged as described in the text. **(E)** qRT analysis of MtrA targets. Expression levels of select genes was measured, normalized to *sigA* and data presented as fold expression relative to the WT strain. The results shown are average from three independent experiments.

The above data support the hypothesis that MtrA activity is intimately associated with replicating cells and is critical for optimal cell division and cell wall metabolism processes. To gain further insights into this issue, we attempted to create and characterize an *M. tuberculosis mtr*A-defective mutant. Our innumerable attempts to create an *mtr*A conditional-expression strain were not successful. Given the sequence similarity of the *mtr*A region (consisting of the *mtr*A, *mtr*B, *lpq*B genes) between *M. smegmatis*, a non-pathogen, and *M. tuberculosis*, we attempted to create and characterize the *M. smegmatis mtr*A mutant as an alternative. The native *mtr*A gene in *M. smegmatis* was replaced with a mutant copy wherein a gentamycin cassette replaced the 198-bp internal coding region by homologous recombination (Figure 5A). The genotype of obtained Δ*mtrA* mutant cells was confirmed by Southern blot hybridization (Figure 5B). The absence of MtrA in the mutant lysates was confirmed by immunoblotting (Figure 5C). These results indicated that the *M. smegmatis mtr*A gene is not essential, unlike the *M. tuberculosis* counterpart (Zahrt and Deretic, 2000; Griffin et al., 2011; our unpublished data). *M. smegmatis*Δ*mtr*A cells were filamentous with branches and buds, indicating cell division and cell shape defects (Figure 5D), and were defective for MtrA-target genes expression (Figure 5E). Expression of the *M. tuberculosis mtr*A (Δ*mtr*A::*mtr*A_TB_) from the amidase (Figure 5D) promoter reversed the cell length and gene expression defects. We also found that production of MtrA_Y 102C_ (Δ*mtr*A::*mtr*A_Y 102C_) as a sole source for MtrA reversed the filamentous phenotype, except that cells were somewhat wider with an altered cell shape (Figure 5D). Together, these results indicate that the *M. tuberculosis mtr*A can substitute for the function of the *M. smegmatis* counterpart and is critical for the regulation of cell division and cell wall metabolism.

### The Inactivation of *mtrA* Affects Susceptibility of *M. smegmatis* to Antimycobacterial Agents

One consequence of the defective cell division and cell wall metabolism of the Δ*mtr*A mutant could be altered susceptibility to antimycobacterial drugs, notably cell wall targeting antibiotics. When tested, Δ*mtr*A was nearly 12-fold more resistant to isoniazid than the parent strain and 16-fold more sensitive to vancomycin (Table 1). Intriguingly, the Δ*mtr*A mutant sensitized to rifampin (Table 1), whereas the parent strain was resistant at the concentrations tested as published (Alexander et al., 2003). The Δ*mtr*A mutant showed a modest increase in sensitivity to ampicillin (Table 1).

**Table 1:** Antibiotic resistance profile of *ΔmtrA* in comparison to the wild type *M. smegmatis*.

Antibiotic	MIC for *M. smegmatis* strains (μg/ml)	
	WT	Δ*mtrA*
Vancomycin	1.5	0.094
Ampicillin	8.0	2.0
Isoniazid	1.0	12.0
Rifampin	>32	0.25

It is pertinent to note that increased susceptibility to the first-line antimycobacterial drug rifampicin with an associated increase in resistance to isoniazid is a characteristic feature of persistent bacilli (see Discussion). The sequencing of *katG* gene (MSMEG*_*6380) confirmed the observed high resistance of Δ*mtrA M. smegmatis* mutant cells to isoniazid is not due to the accumulation of mutation(s) in *katG*.

## Discussion

The response regulators of bacterial two component systems are often required to be phosphorylated by their partnering sensor kinase in order to bind to the target DNA sequences.

The phosphorylation usually occurs under specific, often unidentified conditions and is induced by specific triggers, e.g., change in the pH, osmotic pressure, temperature, concentration of growth limiting substances and ions. The global CHIP-seq analysis is among the most powerful methods currently available for identification of the DNA targets for a given transcription regulator.

This method was previously applied for *M. tuberculosis* overproducing C-terminal FLAG-MtrA (Minch et al., 2015) and N-terminal His-tagged MtrA (Chatterjee et al., 2018). A total of 114 genes were selected as putative MtrA targets in both studies. Using EMSA approach, Minch et al. (2015) confirmed binding of MtrA to the upstream region of Rv0494. EMSA and q-PCR techniques were used to validate the binding of MtrA to upstream regions of *rpfA, rpfC, rpoB, relF, rpfB*, and *whiB4* genes by Chatterjee et al. (2018). Here, we expressed phosphorylation competent MtrA_Y 102C_ mutant protein, able to function in the absence of the cognate sensor kinase MtrB, under the control of acetamide promoter in *M. tuberculosis* and the CHIP-seq analyses were performed from exponential and stationary phases of growth. The CHIP-seq analysis based on anti-MtrA antibodies recognizing MtrA, MtrA-P as well as MtrA_Y 102C_ allowed to identify 278 MtrA targets. Fifty six targets were found to be common to Minch et al. (2015) studies, 27 were common to Chatterjee et al. (2018) and 14 targets were common to both previously published studies. The remaining 195 targets were identified with MtrA_Y 102C_ overproduction strain, exclusively. Among the 14 putative MtrA targets identified together with Minch and Chatterjee, are resuscitation promoting factors: *rpfA* (Rv0867c), *rpfB* (Rv1009), *rpfC* (Rv1884c), and PPE family proteins: PPE19 (Rv1361c), PPE35 (Rv1918c), PPE38 (Rv2352c). The EMSA assay was applied to verify previously identified *rpfB* (Minch et al., 2015; Chatterjee et al., 2018), *ripA* (Minch et al., 2015), *wag31* (Minch et al., 2015) and new targets (*sigD, oxyS, wag31, betP, rv3887, whib3*) of MtrA. The venn diagram showing comparison of MtrA targets identified in our studies with targets identified by Minch et al. (2015) and Chatterjee et al. (2018) is located in the Supplementary Figure S3C. Common targets are also marked in Supplementary Table S3. The MtrA binding site consensus was determined based on the CHIP-seq datasets. The motif consensus found in our study is consistent with our previous findings and it shows a high level of sequence conservation with the MtrA-binding motifs reported for *Corynebacterium glutamicum* (Brocker et al., 2011) and *Streptomyces coelicolor* (Zhang et al., 2017), n-A/G-**T**-a-A-**C**-a and n-G-**T**-n-**A**-C-c-(c), respectively. Since signals promoting MtrA phosphorylation are unknown, applying CHIP-seq analyses for MtrA_Y 102C_ made it possible to identify new targets and expanded the MtrA regulon. On the other hand we have to remember that growth kinetic for RvY102C is affected in stationary phase, reaching it faster than control strains, suggesting that MtrA_Y 102C_, which mimics phosphorylated MtrA, is slowing down the replication and/or cell division, e.g., as a down-regulator of *dnaA* involved in the initiation of DNA replication (Figure 1B, Fol et al., 2006). On the other hand, we cannot exclude that MtrA_Y 102C_ is not fully active and able to replace the function of phosphorylated MtrA for 100%. It may be also associated with partially disturbed regulation of genes involved in the process of cell division. It was previously reported that MtrAY102C is a phosphorylation competent and acts as a gain-of-function (GoF) protein in the absence of MtrB. Its overexpression restores the *ΔmtrB* phenotype in *M. smegmatis* cells and increases the expression of *dnaA, pfbpB, ripA, ftsI* and *wag31* – MtrA targets (Plocinska et al., 2012). The Wag31∼P/Wag31 protein levels were reduced in *ΔmtrB* strain overproducing MtrA_Y 102C_ (Plocinska et al., 2014). The MtrA carrying both D56N (phosphorylation-defective) and Y102C (constitutively active protein) mutations was found to be phosporylation defective although bound to its promoters of *PfbpB* and *PripA*. Also expression of MtrA_D56N-Y 102C_ reversed Δ*mtrB* phenotype (Satsangi et al., 2013). The *in vivo* studies has shown both MtrAY102C∼P/MtrAY102C binds to *oriC* and *mtrA* F2, F4, F5 wild-type boxes (Purushotham et al., 2015). In this study, using the qRT-PCR approach, we have shown increased expression of *ftsI* and decreased expression of *dnaA* in *M. tuberculosis* strain producing MtrA_Y 102C_. The CHIP-seq analysis for MtrA_Y 102C_ performed under active and stationary phase of growth identified comprehensive MtrA∼P regulon.

Here, we showed that several players involved in the cell division and cell wall metabolism processes are members of the MtrA regulon and that MtrA levels and activity are compromised under stress and replication-arrest conditions. We also showed that the *M. smegmatis mtr*A is not an essential gene and that MtrA activity contributes to susceptibility to the antimycobacterial agents rifampin and isoniazid. Together, these results are consistent with the proposal that MtrA controls the cell division and cell wall integrity, affecting the susceptibility of bacilli to some antimycobacterial agents in replicating cells.

Despite being one of the two essential two component systems, the activities of the MtrAB TCSSs are unknown. The results presented in this study provide valuable insights into the environmental conditions promoting MtrAB TCSS activation. We observed here the ability of MtrA to target the key players involved in cell division, such as *fts*I, *dac*B1, *rip*A, *fbp*B, and *fbp*C in actively multiplying cells (Supplementary Table S3). Additionally, MtrA protein levels and activity are reduced under replication-arrest and growth-compromising conditions such as exposure to NO and envelope stress (Figures 3, 4). Admittedly, these studies were based on the evaluation of MtrA levels and MtrA-target expression under limited stress and growth-arrest conditions, although it is known that *M. tuberculosis* encounters multiple stresses upon infection. Although MtrA levels under other environmental stress conditions remain to be evaluated, a consequence of MtrA degradation could be a suboptimal ratio of MtrA∼P/MtrA and, hence, compromised MtrA-regulon expression. This line of thinking leads to a proposal that a growth-arrest state and compromised MtrA activity go hand-in-hand and that replicating cells promote MtrA activation and, hence, are enriched with the optimal MtrA∼P/MtrA levels required for optimal MtrA-target expression. Indeed, MtrA_Y 102C_ overproduction which could lead to an increase in the ratio of MtrA∼P/MtrA is associated with an increase in the expression of MtrA-targets (Figure 1B). Because the DosRS and MprAB systems are induced under NO – and envelope-stress conditions (Bretl et al., 2011), it is reasonable to assume that one effective adaptation strategy of *M. tuberculosis* to cope with NO and envelope stress is the downregulation of the MtrAB with a concomitant upregulation of the MprAB and DosRS, the elements of two component systems. Such a scenario can also be envisioned during intracellular growth and infection. In partial agreement with this line of thought are the reports showing the upregulation of the MtrA targets *rpf*A-E and *rip*A, which aid in cell wall processing and the resolution of the septa upon *M. tuberculosis* reactivation and exit from the dormant and non-replicative persistent states (Mukamolova et al., 2002; Davies et al., 2008; Gupta et al., 2010; Kana and Mizrahi, 2010; Kondratieva et al., 2011).

Our results showing the stabilization of intracellular MtrA levels in the *mpa* mutant background are consistent with the notion that the intracellular MtrA is reduced *via* the Mpa-mediated proteasome pathway. Proteins damaged during NO stress are processed for degradation by the Mpa-dependent proteasome pathway, and this type of proteasomal degradation is critical for optimal *M. tuberculosis* survival upon infection (Darwin et al., 2003; Festa et al., 2010). However, our results also showed that MtrA was degraded, *albeit modestly*, under envelope and NO stress in the *mpa* mutant (Figure 4). These results signal the involvement of Mpa-independent pathways for MtrA processing *in vivo*, although the identity of such pathway(s) is unknown. Unregulated MtrA∼P owing to elevated MtrA production upon infection is detrimental for *M. tuberculosis* proliferation, and the activity of the MtrB sensor kinase is implicated in maintaining optimal ratios of MtrA∼P to MtrA (Fol et al., 2006). Thus, our results showing the reduction of the intracellular MtrA levels under stress and replication-arrest conditions by Mpa and possibly other proteolytic pathways adds another layer of control for monitoring MtrA∼P potential in replicating cells.

The question then arises of what signals in the replicating cells promote MtrA activation. One possibility is that cell wall synthetic and hydrolytic products, which accumulate as a consequence of cell wall metabolism, promote MtrA-regulon activation. Earlier studies indicated that the association between MtrB and the septum promotes MtrA∼P and MtrA-regulon expression (Plocinska et al., 2012). Thus, nascent septa and or cell wall metabolic products could be two of the many triggers that promote MtrA activation and, hence, the output of the MtrAB two component system. Further studies are required to address these issues.

Elucidation of the MtrA regulon and the identification of cell division and cell wall metabolism players as the regulon members combined with the filamentation and cell shape-defect phenotypes of the *M. smegmatis* Δ*mtr*A mutant (Figure 5 and Supplementary Table S3) support the notion that MtrA plays a vital role in the regulation of cell division and cell shape maintenance in mycobacteria. Interestingly, *fts*Z, whose gene product is critical for the initiation of the septal Z-ring and cell division, is not an MtrA target (Fol et al., 2006; Rajagopalan et al., 2010). These data imply that FtsZ rings can be formed independently of MtrA and that MtrA activity impacts cell division and cell wall metabolism at steps subsequent to the FtsZ-ring assembly. It is pertinent to note that response regulators affecting cell division in other bacteria are known. Analyses of their activities lead to a proposal that the MtrA system has departed significantly from the known eubacterial response regulators affecting cell division and cell wall metabolism processes. For example, the essential response regulator CtrA of *Caulobacter crescentus* promotes the cell cycle-dependent ordered expression of *fts*Z, *fts*Q, and *fts*A, the core genes required for the initiation and progression of cell division. CtrA∼P has been shown to act as a transcriptional repressor of *fts*Z and an activator of *fts*Q and *fts*A (Kelly et al., 1998; Wortinger et al., 2000). Similarly, the essential response regulator YycF in *B. subtilis* affects the expression levels of *fts*Z and *fts*A (Fukuchi et al., 2000) and those genes are involved in cell wall metabolism such as autolytic enzymes and hydrolases (Bisicchia et al., 2010; Fukushima et al., 2010). The *fts*A-like gene is absent in the mycobacterial genomes; MtrA also distinguishes itself from CtrA and YycF in that it specifically targets the players involved in septum and cell wall synthesis, i.e., *fts*I, *dac*B1, *sep*F, *wag*31, and cell wall expansion and remodeling, i.e., *rip*A, *rpf*A-E, *fbp*B, *fbp*C (Figures 3, 4, Plocinska et al., 2014). Thus, unlike in the other organism, MtrA activity targets septum synthesis and cell wall expansion, the late steps of the cell division process. This leaves open a question how the regulation and stabilization of FtsZ-mediated Z-ring assembly in mycobacteria is accomplished.

Antibiotic susceptibility experiments with *M. smegmatis*Δ*mtr*A mutant clearly showed that MtrA impacts tolerance to antimycobacterial drugs. The Δ*mtr*A mutant is 16-fold more resistant to isoniazid than the parent strain but still significantly sensitive to rifampin (Table 1). Isoniazid is a first-line mycobactericidal drug that rapidly kills actively dividing bacilli, whereas rifampin is a sterilizing drug that kills both replicating and persistent bacilli (Tomasz et al., 1970; Dickinson et al., 1977). It is pertinent to note that increased tolerance to isoniazid with continued susceptibility to rifampin is a characteristic feature of persistent bacilli and a clinical feature of latent tuberculosis infection (Mc, 1959). Although the antibiotic susceptibility experiments were based on *M. smegmatis*Δ*mtr*A, given the conserved organization of the MtrA region and the players involved in cell division and cell wall metabolism in *M. tuberculosis* and *M. smegmatis*, it is logical to speculate that MtrA activity is reduced during persistence, thereby impacting antibiotic tolerance.

The MtrAB TCSS is conserved in high G+C rich actinobacteria such as *Corynebacterium glutamicum, C. diphtheria* and *Streptomyces* sps (Hoskisson and Hutchings, 2006); *mtr*AB genes can be deleted in *C. glutamicum* (Moker et al., 2004) much like the situation with the MtrAB system of *M. smegmatis* (this study and Plocinska et al., 2012). Also, the *C. glutamicum mtr*AB mutant is sensitive to vancomycin and ampicillin (Moker et al., 2004), like the *M. smegmatis* counterpart.

On the other hand, in the contradiction to our observations, Li and colleagues reported that *M. smegmatis* construct expressing antisense *mtr*A RNA is sensitive to isoniazid and did not show susceptibility to rifampicin (Li et al., 2010). Although the levels of MtrA protein are unknown, the *mtr*A expression is reported to be reduced 0.38 fold under antisense *mtr*A condition (Li et al., 2010). Our studies were carried out in a clean *mtr*A mutant background, hence cannot directly be comparable with the reported *mtr*A antisense studies.

## Author Contributions

PG, RP, KS, AS, EP, and RD conceived and designed the experiments. PG, RP, KS, AS, EP, and RD performed the experiments. PG, RP, KS, AS, EP, RD, JD, MR, and MM analyzed the data. MR, MM, JD, PG, and RP wrote the paper.

## Conflict of Interest Statement

The authors declare that the research was conducted in the absence of any commercial or financial relationships that could be construed as a potential conflict of interest.
