# Supplementary material for: MtrA Response Regulator Controls Cell Division and Cell Wall Metabolism and Affects Susceptibility of Mycobacteria to the First Line Antituberculosis Drugs
Source: Front Microbiol. 2018 Nov 23;9:2839. doi: 10.3389/fmicb.2018.02839 (PMC6265350; doi:10.3389/fmicb.2018.02839)
Supplement: Supplementary file 1 [file Data_Sheet_1.doc]

**Supplementary Data:**

**MtrA response regulator controls cell division and cell wall metabolism and affects susceptibility of mycobacteria to the first line antituberculosis drugs**

Purushotham Gorla^1^, Renata Plocinska^*2^, Krishna Sarva ^1^, Akash T. Satsangi ^1^, Emmanuel V. Pandeeti^1^, Robert Donnelly^3^, Jaroslaw Dziadek ^2^, Malini Rajagopalan^*1^ and Murty V. Madiraju^*1^

1. **Methodology for ChIP-Seq**
2. **Figures and Legends**
3. **Tables**

**Methodology and the MtrA-regulon**

*ChIP-Sequencing -* Purified ChIP-DNA was end-repaired, polished and used to prepare barcoded libraries following the manufacturers’ protocol (Applied Biosystems (ABI), Foster City, CA P/N 4443713). Briefly, DNA fragments were washed by binding to AmPure XP Beads (Beckman Coulter Genomics, Danvers, MA, P/N A63881), mixed with the multiplex library P1 and the appropriate barcode double stranded adaptors (ABI, P/N 444837), and ligated, and the generated library was nick translated and amplified using multiplex library PCR primers. Multiple purification steps using the AmPure beads were performed to isolate the amplified fragments prior to SOLiD sequencing. Various quantification methods were used to check the concentration of the libraries, including quantitative PCR, the PicoGreen Assay (Invitrogen, Carlsbad, CA) and a Bioanalyzer (Agilent, Santa Clara, CA). The barcoded libraries were then pooled at equimolar amounts such that the concentration of the pool was 500 pM. Next, emulsion PCR (ePCR) reactions were performed on the library pool according to the manufacturers’ protocol (ABI, P/N 4392173). The aqueous and oil phases were prepared separately and then emulsified using an ULTRA-TURRAX tube drive (IKA, Wilmington, NC). The resulting emulsion was then transferred into a 96-well plate and amplified for 60 PCR cycles. The emulsions were broken using 2-butanol, and the templated beads were purified and washed to remove any residual 2-butanol, oil, and aqueous phase containing PCR reagents. The templated bead enrichment procedure isolates beads with full-length extension products that were produced by oligo-hybridization using the sequence of the P2 (barcoded) primer and ePCR. The yield of the enriched templated beads from one full-scale ePCR was 150 to 300 million beads. The P2 enriched beads were 3’-terminally modified with the bead linker by a terminal transferase to prepare them for covalent attachment to the glass slide. The end-modified enriched beads were deposited on the slides, incubated at 37°C for one hour, and mounted on the SOLiD 3.5 instrument (ABI). The slides were run on the instrument collecting data for 50 bases per read and the 5 base barcodes. The barcodes were sorted on the instrument and exported for further analysis. Quality control analysis of the data was performed on the instrument.

*Data Analysis and Motif prediction -* Data from the SOLiD 3.5 instrument was exported to an external server where it was analyzed using the CLC Genomics Workbench software package (CLC bio, Cambridge, MA, USA). The reads from the instrument were mapped to the H37Rv reference genome (NCBI accession #NC_000962) using color space alignment. ChIP peaks were detected using the ChIP-Seq Analysis tool in the package with the following criteria: for mapping we chose length fraction 0.7, similarity 0.8, deletion and insertion cost 3 each whereas for ChIP-Seq analysis window size of 250 and maximum FDR of 1% were considered. ChIP-Seq peaks for days

3 and 8 were annotated with the Artemis genome browser (http://www.sanger.ac.uk/resources/software/artemis/), and the ChIP-Seq peaks that overlap in the duplicates and are present upstream of a gene or overlapping the transcriptional start site of a gene were considered for the identification of the MtrA motif. All the targets obtained from the ChIP data were grouped based on the function of the ChIP peak associated genes [^1^](#_ENREF_1). When a ChIP peak is associated with two divergently transcribed *orfs*, both the *orfs* were considered as targets of MtrA. The ChIP peaks from duplicate samples of day 3 were analyzed using the MEME motif prediction tool (http://meme.sdsc.edu/meme/cgi-bin/meme.cgi) choosing the maximum motif width as 30 nt and the minimum width as 6 nt. The distribution of motif occurrence per sequence was selected as zero or one. The MtrA motif predicted for day 3 was searched in the ChIP-Seq peaks of day 8 by scanning the sequence using the default parameters of MAST tool. The sequence alignments obtained from MEME were used as an input file for the creation of the MtrA motif using the Web logo tool (http://weblogo.berkeley.edu/).

**Figure S1**

**
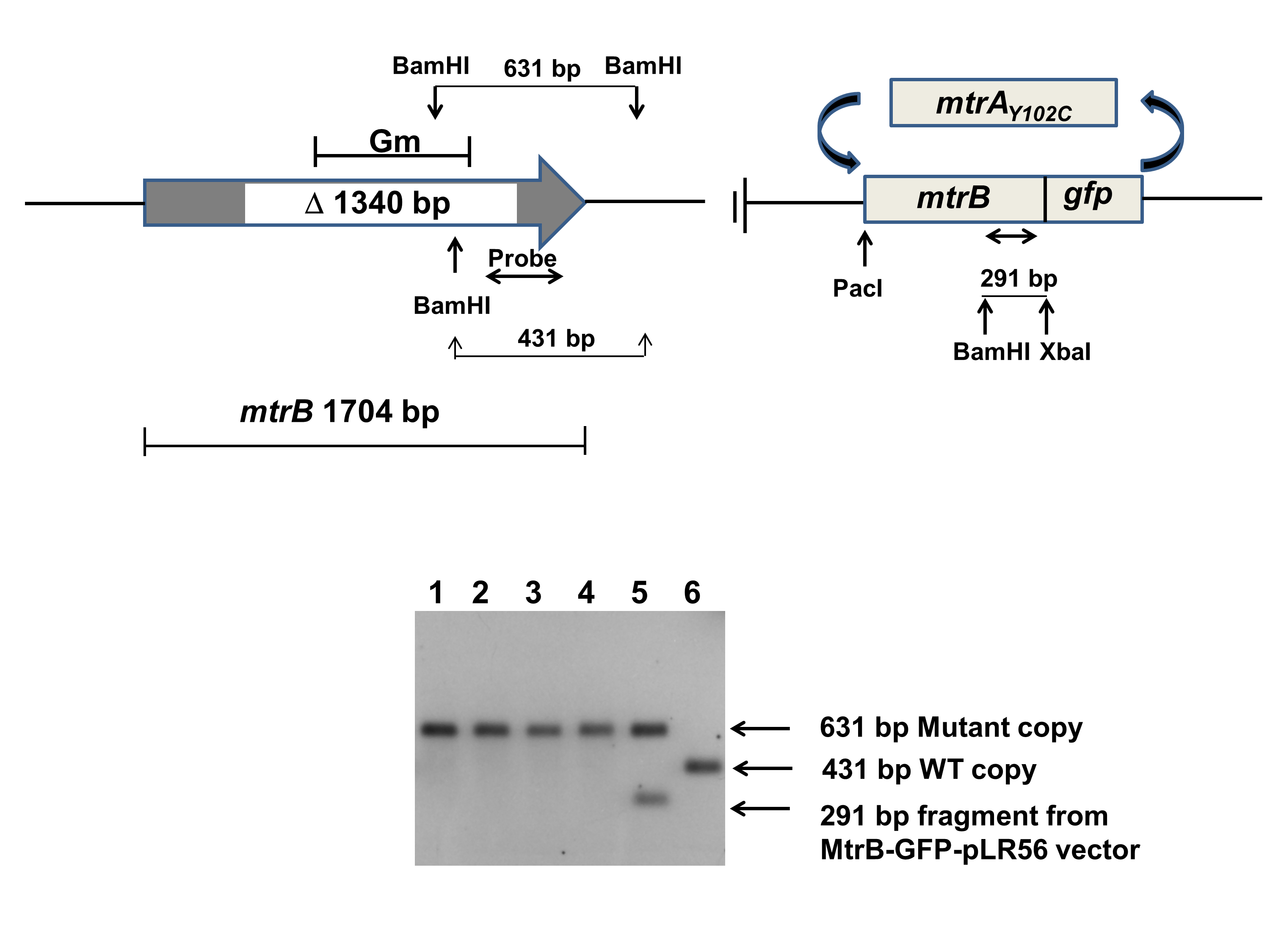
**

**Fig. S1** Construction of *M. tuberculosis* Δ*mtrB* mutant strain complimented with MtrA_Y102C_.

1. Schematic representation of *mtrB* native and complemented loci.
2. Southern blot confirming the genotype of Δ*mtrB*::Pami *mtrA*_Y102C_ mutant. The chromosomal DNAs were isolated, digested with BamHI/PacI/XbaI restriction enzymes, transferred to Hybond-N+ membrane (GE Healthcare) and probed with *mtrB* fragment. The detected band for wild type copy corresponds to 431 bp, mutant copy 631 bp. The present 291 bp band corresponds to Ptet::*mtrB*-GFP vector being swapped with Pami::*mtrA*_Y102C_.

Legend: 1 – 4 - Δ*mtrB*::Pami *mtrA*_Y102C_ # 1, 2, 3, 4; 5 - Δ*mtrB*::Pami *mtrB*-*gfp*; 6 – wild type.

Gm indicates gentamycin cassette.

**Figure S2**


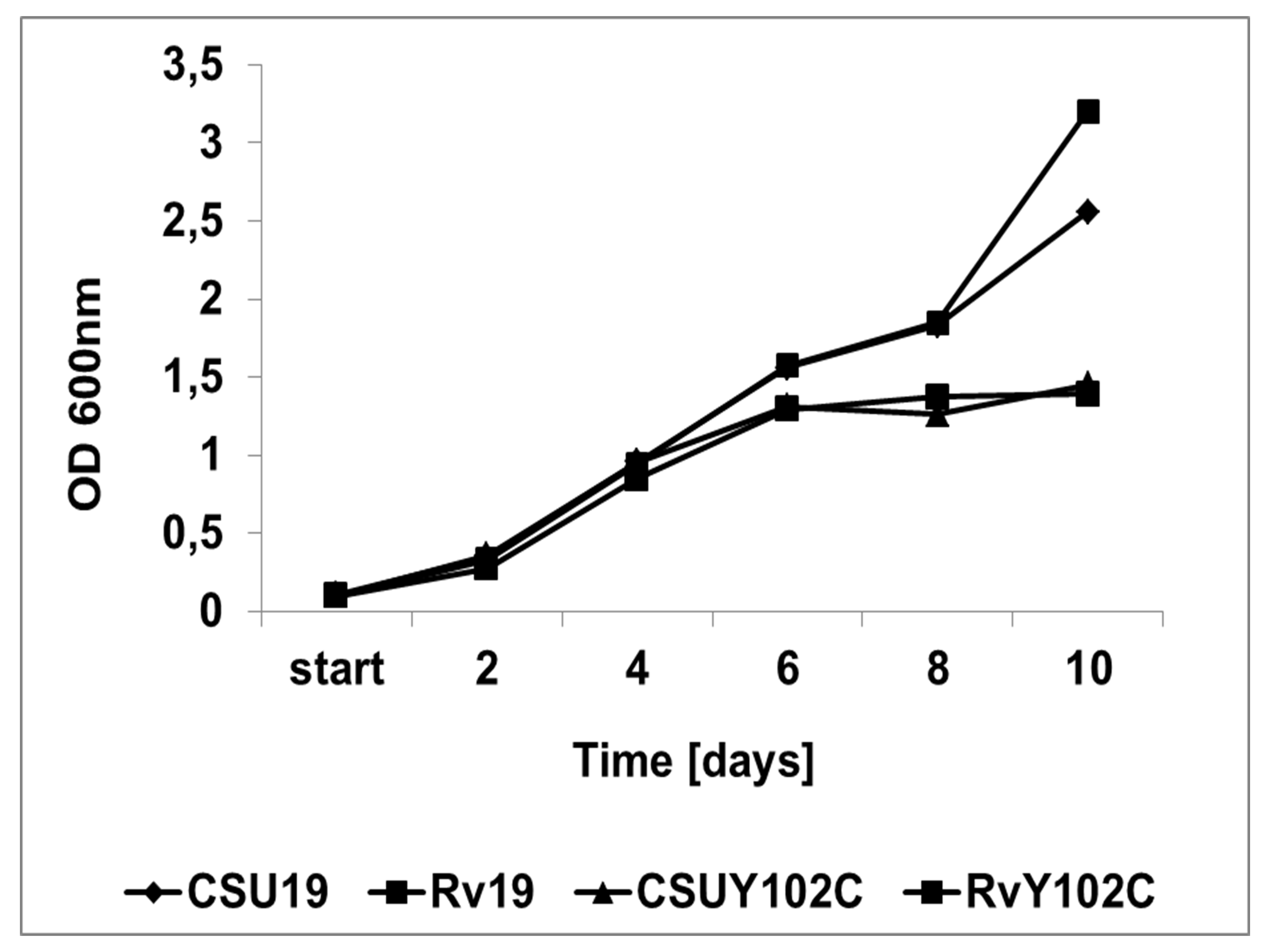


Fig S2. Growth of *M. tuberculosis* H37Rv (Rv) and CSU#1 strains (CSU) carrying pJfr19 empty vector (19) or *mtrA*_Y102C_ plasmid (Y102C). The *M. tuberculosis* actively growing cells were diluted to 0.1 OD600 and the optical density was measured at designated time points.

**Figure S3**


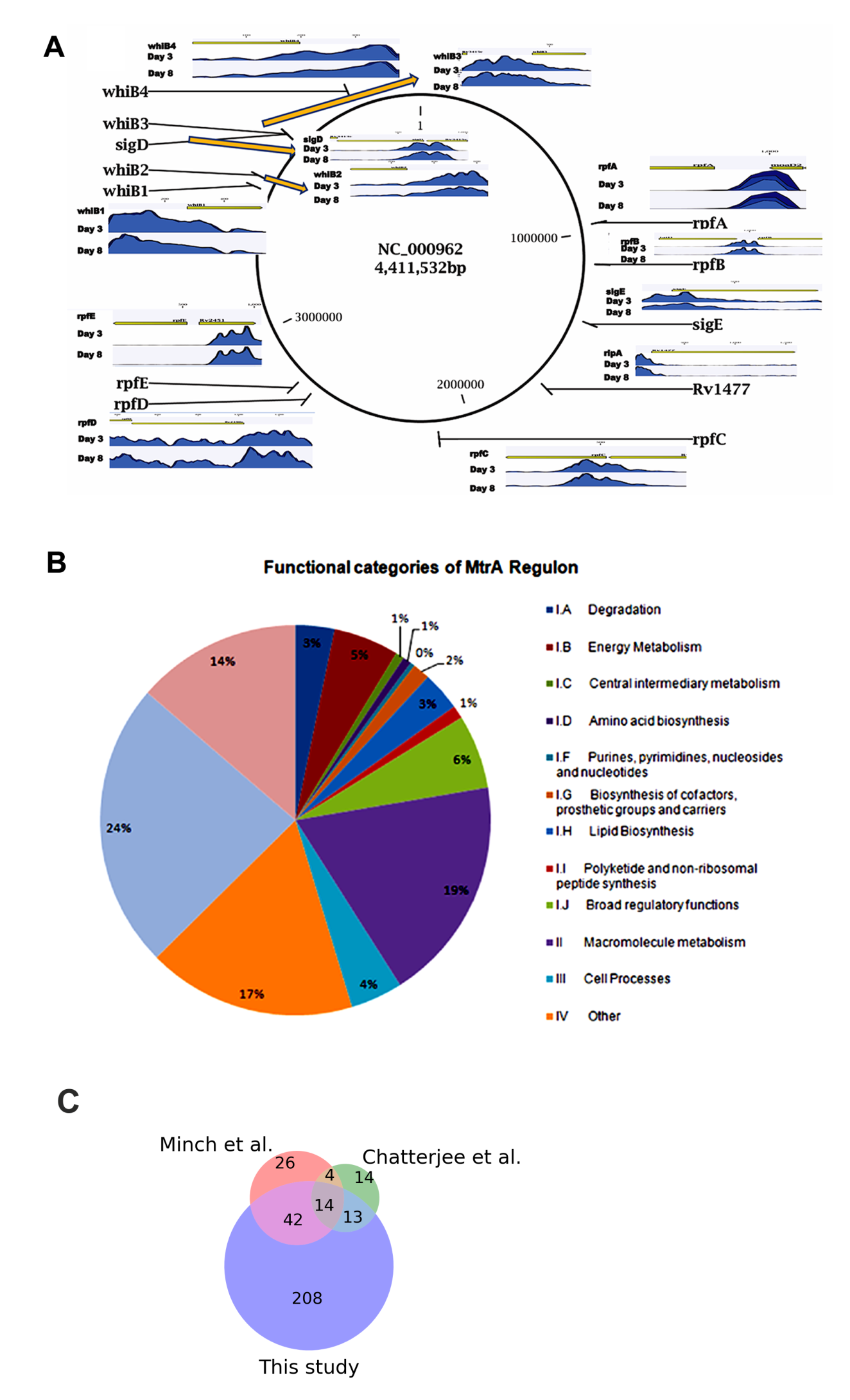


**Fig. S3** (A) Circular genomic map showing select MtrA targets. (B) Select MtrA ChIP-Seq peaks for exponential (day 3) and stationary (day 8) targets are presented. These targets include all five annotated *rpf* genes, *rpfA, rpfB, rpfC, rpfD* and *rpfE,* which are believed to exhibit cell wall hydrolase activities critical for cell wall expansion [^2-4^](#_ENREF_2) , are individually nonessential but contribute to persistence when combined [^2^](#_ENREF_2),[^5-7^](#_ENREF_5); four WhiB family iron-sulfur transcription factors that play important roles in cell division, virulence, starvation, oxidative stress and drug resistance [^8-10^](#_ENREF_8) ; two ECF- sigma factors *sig*E and *sig*D, which are induced during stationary phase and in response to SDS exposure and membrane stress [^11-15^](#_ENREF_11) ; and *rip*A, an essential cell wall hydrolase believed to be critical for *M. tuberculosis* reactivation. Thus, the ability of MtrA to target a wide array of unlinked genes supports a notion that MtrA~P levels likely impact their activities and the associated processes. (C) Venn diagram representing comparison of CHIP-seq analysis by Gorla et al., Chatterjee et al. and Minch et al.

**Figure S4**


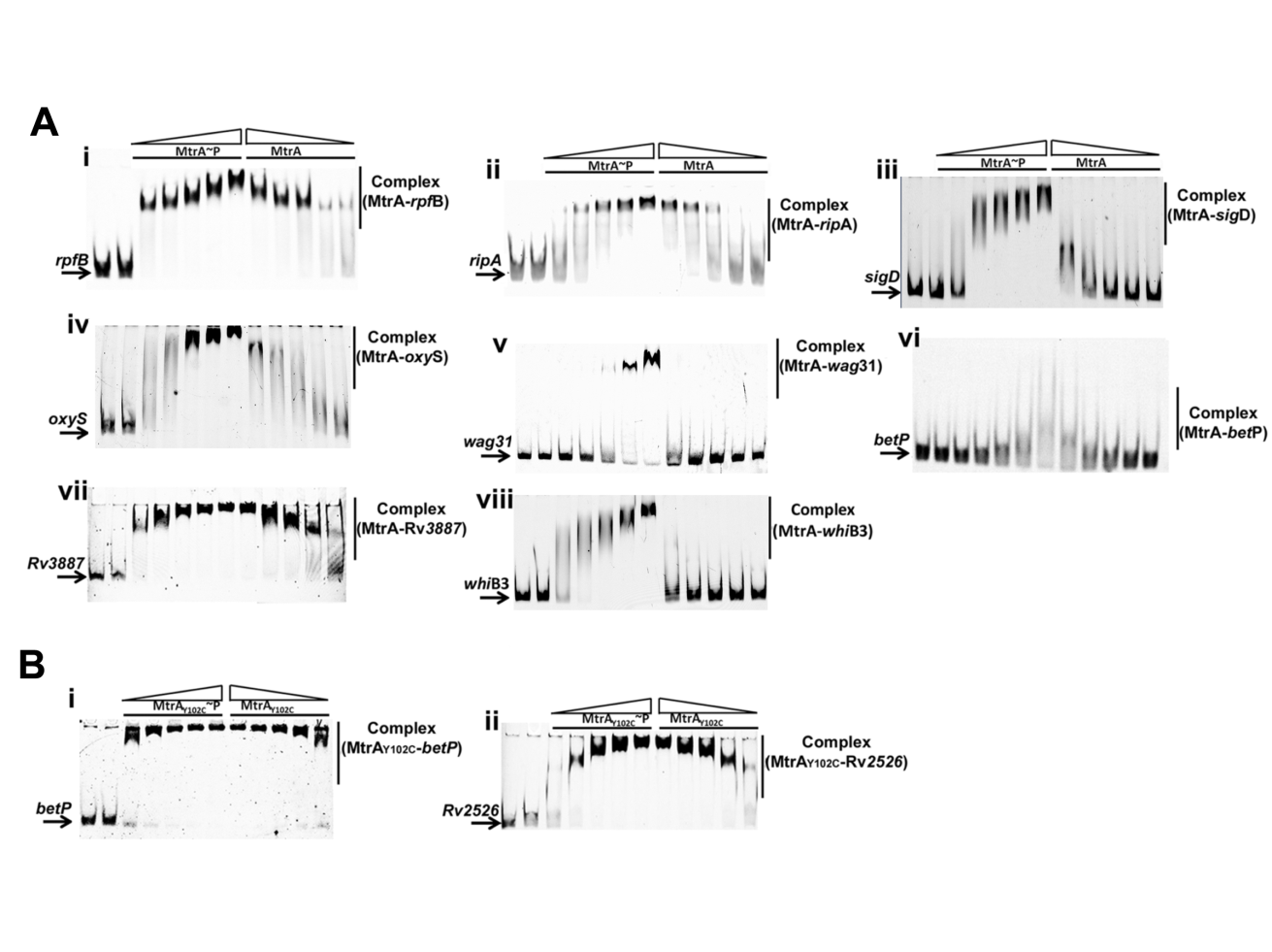


**Fig. S4 EMSA analysis of MtrA binding to select promoter targets.** *(A)* MtrA protein was phosphorylated by EnvZ and MtrA or MtrA~P at 1, 2, 4, 6 and 10 µM concentration was incubated for 10 min with 200 fmoles of FAM-labeled *Prpf*B (panel i), *Prip*A (panel ii), *Psig*D (panel iii), *PoxyS* (panel iv)*, Pwag31* (panel v)*, PbetP* (panel vi)*, Prv3887c* (panel vii) and *whi*B3 (panel viii). As control, unphosphorylated MtrA was incubated with the respective promoters. The DNA-protein complexes were resolved in 5% polyacrylamide gels at 4^0^C, gels were scanned with a Molecular Imager (Fx) and data analyzed using QuantityOne software. *(B)* MtrA_Y102C_ binding to *PbetP* (panel B-i), and *Pr*v2526 (panel B-ii). MtrA_Y102C_ was phosphorylated by EnvZ and the protein-DNA complexes were resolved on acrylamide gels and analyzed as described above. Given the differences in the phosphorylation potential between MtrA and MtrA_Y102C_, it is possible that binding to these targets is promoted in the presence of higher pools of MtrA_Y102C_~P.

**Figure S5**


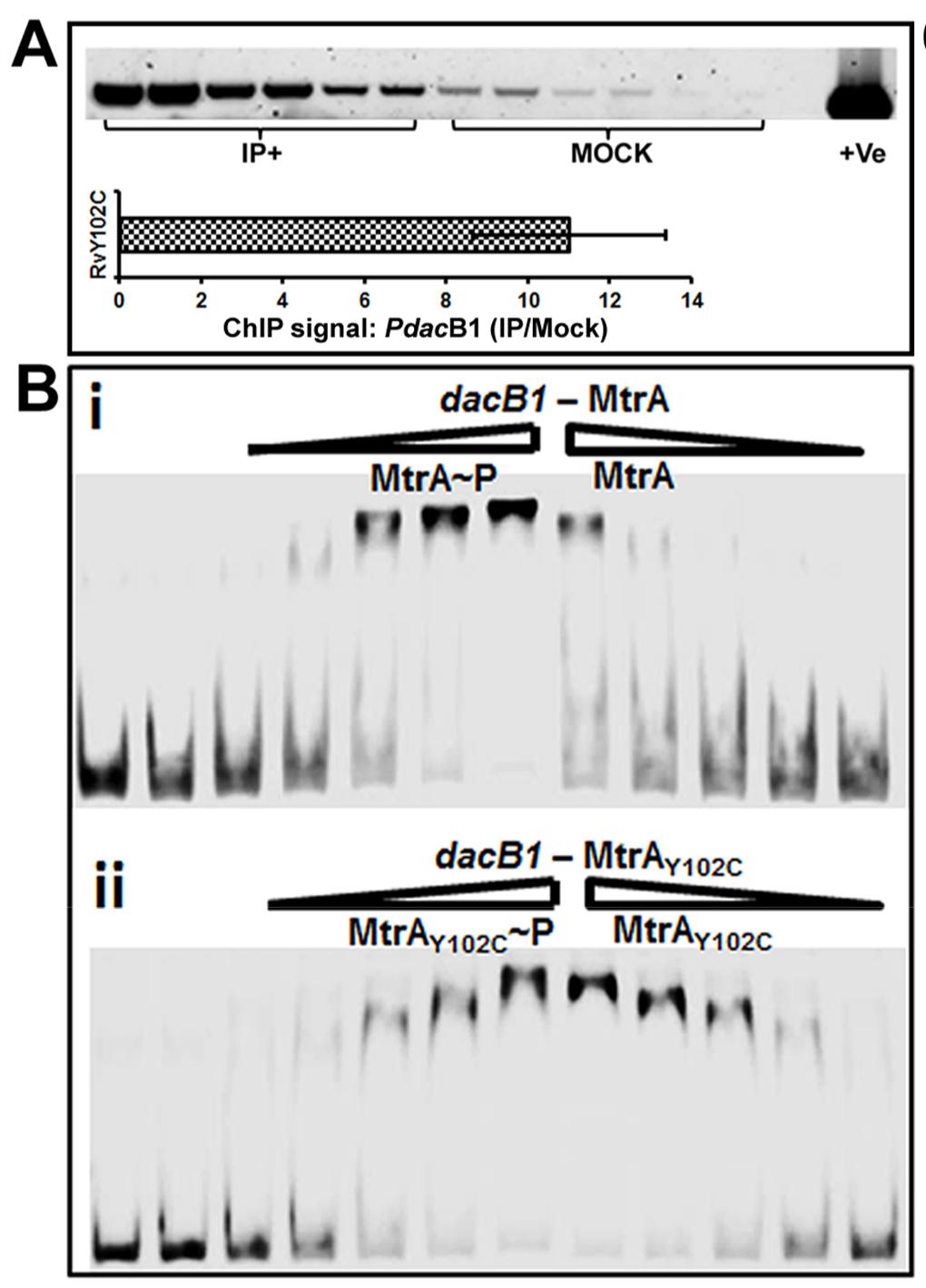


**Fig. S5** ***PdacB1*** **is an MtrA target***.* (A) ChIP analysis of *dac*B1 region enriched from *M. tuberculosis* cells overproducing MtrA_Y102C_. The ChIP assays were performed with MtrA specific antibodies followed by PCR of *Pdac*B1 (MtrA target) or *Pfts*Z (non-target). The ratio of IP to the mock (no antibody set) signal was determined and normalized against the *Pfts*Z promoter value and is shown on the Y-axis. The data shown are MtrA (IP/Mock) normalized to FtsZ (IP/Mock). (B) Binding of MtrA (panel i) and MtrA_Y102C_ (panel ii) to *Pdac*B1 target. The oligonucleotides used to prepare FITC labeled *dac*B1 DNA substrate are shown in Table S1. DNA-binding assays were performed essentially as described under Fig. S3.

**Figure S6**


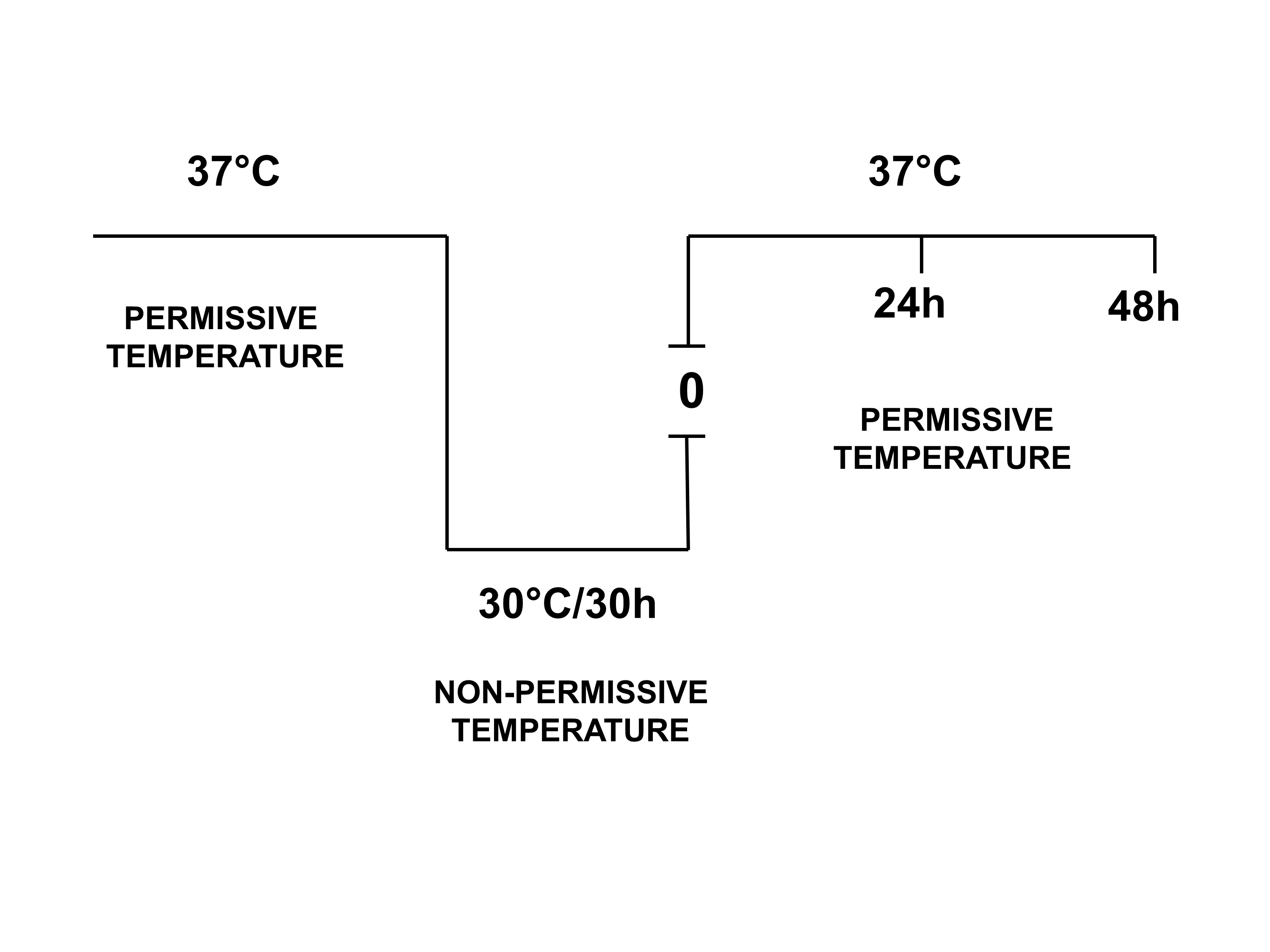


**Fig S6**. Diagram representing a typical synchronization plan for *M. tuberculosis* *dnaA* temperature sensitive mutant cells.


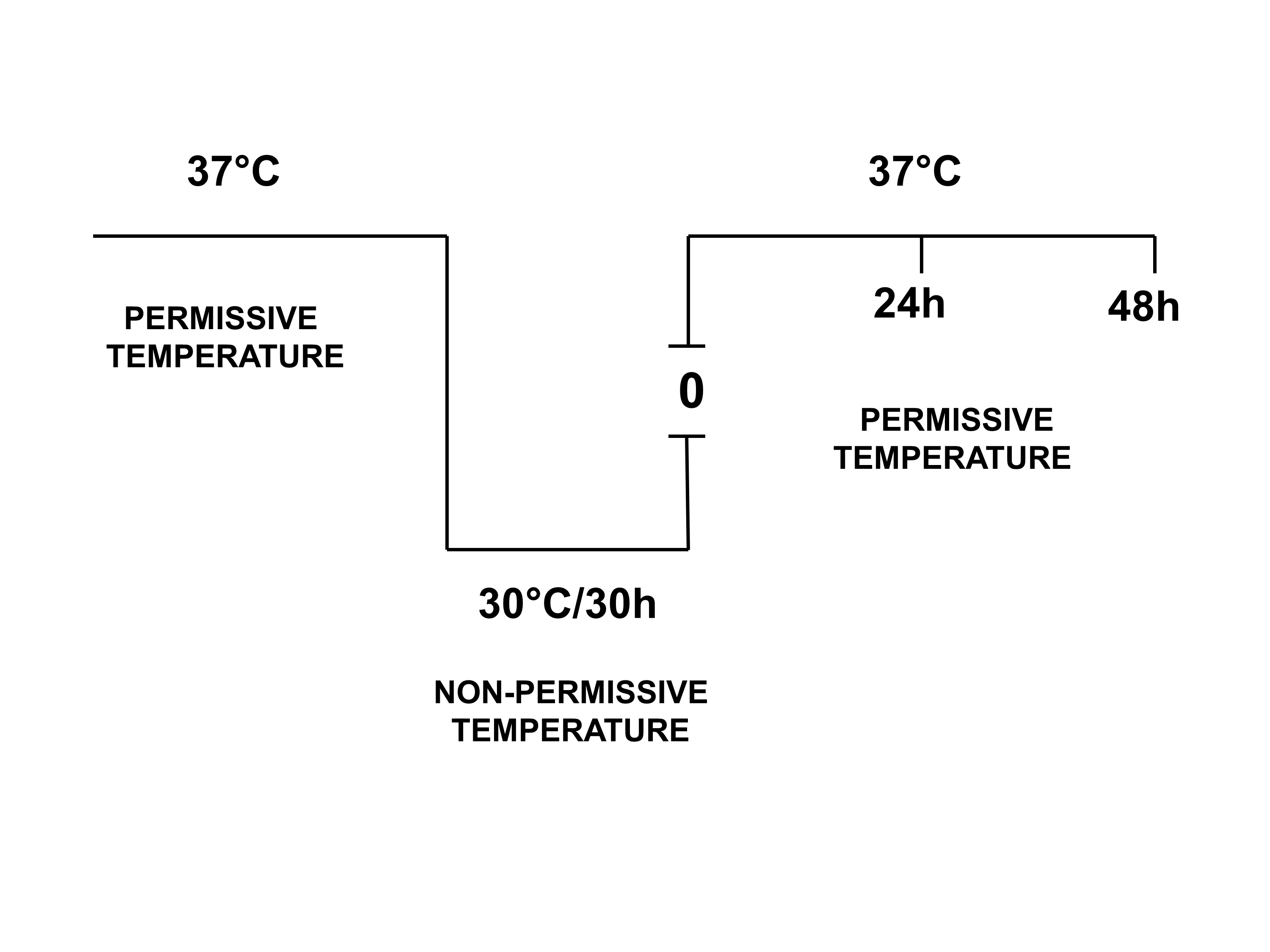


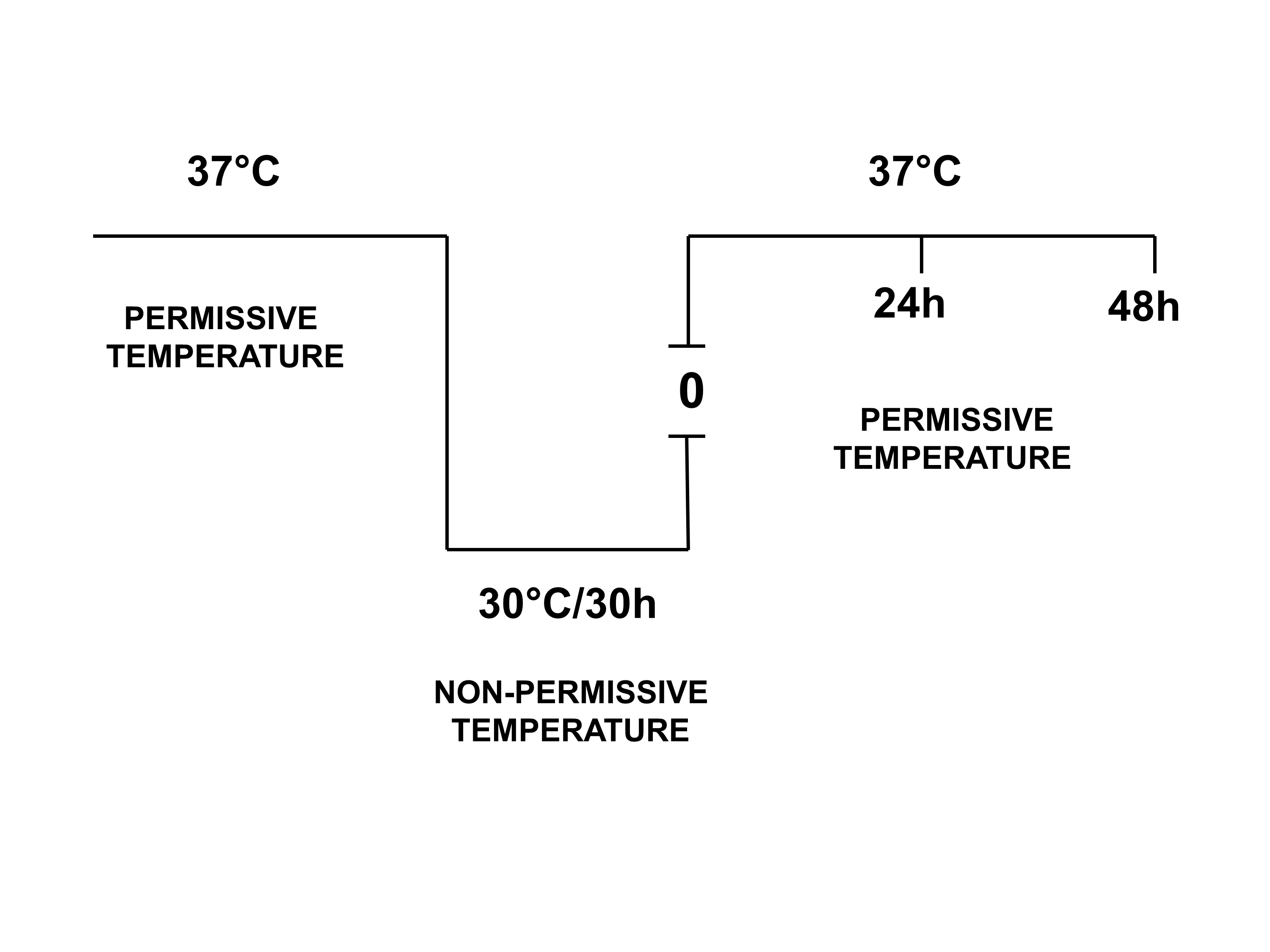


##

## **Table S1. Oligonucleotide primers used in this study**

| **qRT-PCR** | | |
| --- | --- | --- |
| **Target/gene** | **Name of Primer** | **Sequence (5’->3’)** |
| 16Sr RNA | 16Sr RNAqRT1  16Sr RNAqRT2 | ggccggctacccgtcgtc gagatactcgagtggcgaac |
| *mtr*A | mtrAqRT1 mtrAqRT2 | cgacctgttggtcgcattgg cgctggacatgcacgttcac |
| *mtr*B | mtrBqRT1 mtrBqRT2 | agcgagctggaccgattcgag cgcgttgttgaccgttgtgc |
| *sigE* | sigEqRT1 sigEqRT2 | gatgagctggtccgtcagcac gtgcccggctggtaattctgg |
| *sepF* | sepFqRT1 sepFqRT2 | ggtcatcatggatctggtgtc tcgcgaccttgtcgaacgag |
| *wag31* | wag31-qRT1 wag31-qRT2 | cggggatgaacgaggaacagg ggccagcatcttgtccgactcg |
| *ftsI* | ftsIqRT1 ftsIqRT2 | tggtgcaacgcgatccgatg cagccagggttgatctgctg |
| *dacB1* | dacB1qRT1 dacB1qRT2 | gcaccaaagtcggggtgaac gccacgcgtaccggtagaac |
| *rpfB* | rpfBqRT1 rpfBqRT2 | caactcgcgatgaccgacac cgttgagctgcaccgtcttg |
| *ripA* | ripAqRT1 ripAqRT2 | tggaaaccgctcgggacaac aaggtgtcgaaccggtgctg |
| *fbpB* | fbpBqRT1 fbpBqRT2 | tcaggggatggggcctagcc gcttggggatctgctgcgta |
| *sucC* | Rv0951-qRT1 Rv0951-qRT2 | tggccatgccgagttcgagg ctcaccggcatacgcgacga |
| *dnaA* | dnaA-TB1 dnaA-TB2 | agtccggtcctcggcaagac tgaagtcgttggtgaattcctc |
| *mprA* | mprAqRTF mprAqRTF | tctccgacctgacgctggac gcggattggcgatcagcatc |

| *pfkB* | MR123pfkBRT1 MR123pfkBRT2 | gctgatcctggatacatctgg agttcggatccgacgcactc |
| --- | --- | --- |
| *chiZ* | Tb-chiZqRT1 Tb-chiZqRT2 | ggtcgggatgtcgaggactg gccaccaaaccaagccagag |
| ***M.smeg qRT-PCR oligos*** | | |
| *sigA* | sigAqRT_F sigAqRT_R | gtgggcagcgaccaaagcaag acttcgccgctgttcgcttg |
| *mtrA* | MR199-Msmg-MtrA_F MR199-Msmg-MtrA_R | tcactggccgagatgctcac cacatcgatgccgttcatcc |
| *wag31* | wag31-Msmeg-qRT1 wag31-Msmeg-qRT2 | ttccgagatcatgggaaccattg gagctgcgactccaggtaggtc |
| *ftsI* | MR197-Msmg-FtsIF MR197-Msmg-FtsIR | ccgacggttcggtgacctac cttcgccatctggacctgc |
| *dnaA* | dnaA-Msmeg-qRT1 dnaA-Msmeg-qRT2 | cttcatcaactcgctgcgtgac tggaagaactcctcctggatgc |
| *ripA* | ripA-MsmgqRT1 ripA-MsmgqRT2 | gttcctgcagaagctcggaatc cacgagtagggcacaccgatc |
| *fbpB* | Ms85BqRT1 Ms85BqRT2 | ttcgagatgttcctcgactc ccacttgtaggtgacgcag |
|  | | |
| **ChIP assay** | | |
| *Pfts*Z | MVM508 MVM238 | tgcccgccgcgtatcggcgc gcttcctccctggtggggct |
| *Pdac*B1 | ChIPdacB1-F ChIPdacB1-R | caccgacgaccgcgggga ctgtgcagagtaggtgatcggc |

**EMSA**

| *rpf*B | FITC-*rpf*B-F FITC-*rpf*B-R | cggtgattgggtctgatga tctagatatccatggatcc | |
| --- | --- | --- | --- |
| *ripA* | FAM-*rip*A-F  *rip*A-R | ttgccgcgctaaccgggcgg tcgactcagatctcctagggctcaa | |
| *sig*D | FAM-*sig*D-F  *sig*D-R | gcggtgaattcttcgttaacggcatattac cctgaagcttagtggcgtcaccgatg | |
| *oxy*S | FAM-*oxy*S-F  *oxy*S-R | ccggaattccaccgagacacgggtgtg cccaagctttggttcgttatctatccgttgtg | |
| *wag*31 | FAM-*wag*31-F  *wag*31-R | gaggaattcttgagagacggttcggcc ggcaagcttgtagccgtggtgcaactacc | |
| *bet*P | FAM-*bet*P-F  *bet*P-R | cgggaattcgcagtggcgttacgctc gggaagcttcaagctccgtttctcac | |
| *rv3887* | FAM-*3887*F  *3887*R | cgggaattccgccgccgatgcacgc gggaagcttgtcggtgcggcatcactttc | |
| rv2526 | FAM-Rv*2526*-F Rv*2526*-R | cggaattcgagagaccgacatatgccac gggaagcttgcgacgattttgcccattgc | |
| whiB3 | FAM-whiB3-F  whiB3-R | cggaattcgcgcaacggtccgata gggaagcttggattccctctctg | |
| *dac*B1 | *dac*B1-FAMF *dac*B1-FAMR | caccgacgaccgcgggga ctgtgcagagtaggtgatcggc | |
| **Cloning** | | | |
| **Name of primer** | **Sequence (5’->3’)** | | **Construct**  **created** |
| RvmtrBGR1PstI  RvmtrBGR2HindIII | aactgcagtcatcccaacgcagccgg  cgaagctttccctcacgggtacgtcggtc | | pDR55 |
| RvmtrBGR3HindIII  RvmtrBGR4KpnI | cgaagcttactgaggccccgtgtcatcg  ggggtaccggcgattttgcgtctgctcg | | pDR56 |
| MR176dacBF-NdeI- MOD  MR176dacB-  modXba | ggaattccatatggccttcttacgttcggtatcg  atcggtctagaactagtgctgcggccggcg | | pPG2 |
| SepF-NdeI  MR92rSepF-XbaI | attcccatatgaatagtcactgtagtcac  ttgctctagattggtaggcgtagaacccgg | | pKS23 |
| MVM188 XbaIF-  GFPednKpnI | gctcatgaaacaacaacctgcagatgagtaaag  ggggtaccctatttgtatagttcatcca | | pKS24 |
| MVM409-MtrA-F Y99C_R | ccggaattccatatgaggcaaaggatttt gaacggcttcatgatgcagtcgtcggcgcccg | | pDS15a |
| MtrAsmegGR1 | cagtactaccggccgcctgctgatc | | pDR45 |
| ScaI |  | |  |
| MtrAsmegGR2 | caagcttgagtgcctggctgccgtcg | |  |
| HindIII |  | |  |
| MtrAsmegGR3 | caagcttagccgaaggagctcgtcgcg | | pDR47 |
| HindIII |  | |  |
| MtrAsmegGR4 | gttaattaacgacaattcggccacgccc | |  |
| PacI |  | |  |

**Table S2. Strains and Plasmids used in this study**

| **Strains** | | |
| --- | --- | --- |
| **Name** | **Description** | **Reference** |
| TOP10 TOP10-GOLD | *Escherichia coli* strains | Invitrogen Inc. |
| MC^2^155 | *Mycobacterium smegmatis* | Laboratory  stock |
| MC^2^155 (pPG2) | *M. smegmatis* cells overproducing *M. tb* specific DacB1-mcherry | This study |
| MC^2^155 (pKS24) | *M. smegmatis* cells overproducing *M. tb* specific SepF- GFP | This study |
| MC^2^155 (pRD73) | *M. smegmatis* cells overproducing *mtrB_TB_-gfp* | [^16^](#_ENREF_16) |
| ∆*mtr*A | *M. smegmatis mtrA deletion strain* | This study |
| ∆*mtr*A *Pami::mtrA_TB_* | *M. smegmatis* ∆*mtr*A with pMG78 | This study |
| ∆*mtr*A *Pami::mtr*A_D56N_ | *M. smegmatis* ∆*mtr*A with pMG129 | This study |
| ∆*mtr*A *Pami::mtr*A_Y102C_ | *M. smegmatis* ∆*mtr*A with pDS4 | This study |
| *dna*Acos | *M. tuberculosis dna*Acos strain cold-sensitive for replication initiation at 30^0^C | [^17^](#_ENREF_17) |
| H37Rv | Virulent wild type *Mycobacterium tuberculosis* | Laboratory stock |
| ∆*mtrB* | *M. tuberculosis mtrB* deletion strain | This study |
| ∆*mtrB Pami::mtrA_Y102C_* | *M. tuberculosis* ∆*mtrB* with pDS4 | This study |
| Rv-pDAM | H37Rv overproducing truncated MtrA | This study |
| CSU#1 | Clinical strain of *M. tuberculosis* | TBRU, CO |
|  |  |  |
| *M. tuberculosis*  (pPG2) | *M. tuberculosis* cells overproducing DacB1-mcherry | This study |
| *M. tuberculosis*  (pKS24) | *M. tuberculosis* cells overproducing SepF -GFP | This study |
| Rv19 | H37Rv with pJFR19 | [^18^](#_ENREF_18) |
| Rv78 | H37Rv overproducing MtrA from pMG78 plasmid | [^18^](#_ENREF_18) |
| RvY102C | H37Rv overproducing MtrA_Y102C_ from pDS4 plasmid | This study |
| RvY102C-B | H37Rv overproducing MtrB along with MtrA_Y102C_ from pDS15 plasmid | This study |
| Rv∆*mp*A | *M. tuberculosis* CDC1551 MpA proteasome mutant (transposon mutagenesis mutant) | [^19^](#_ENREF_19) , BEI Resources |
| **Cloning Vectors** | | |
| pLR52 | *E. coli* – *Mycobacterium* shuttle vector, replicating,  with *tet* promoter and *tet* repressor, Hyg^r^ | [^20^](#_ENREF_20) |
| pJFR19 | Integration proficient *Mycobacterium* vector with amidase promoter, Hyg^r^ | [^18^](#_ENREF_18) |
| pGOAL17 | Plasmid carrying *Pac*I cassette, Amp*^r^* | [^21^](#_ENREF_21) |
| pMV306K | Integration proficient *Mycobacterium* vector, promoter-less, Km^r^ | Cliff Barry, NIH |
| pUC57 | Plasmid cloning vector in *E. coli* | Genescript USA Inc. |
| pGEM-TE | TA cloning vector system | Promega Corp. |
| p2NIL | Non-replicating recombination vector, Km^r^ | [^21^](#_ENREF_21) |
| **Plasmids** | | |
| pPG2 | The coding sequence of *dac*B1-*mCherry* was amplified and cloned in pJFR19, an integrating vector | This study |
| pKS23 | The coding sequence of *sep*F was amplified and cloned into pJFR19, an integrating vector | This study |
| pKS24 | The *gfp* was amplified and cloned downstream to *sep*F gene in pKS23 to generate *sep*F-*gfp* fusion | This study |
| pMG78 | *Mtb* specific *mtr*A_WT_ was amplified and cloned into pJFR19, an integrating vector | [^18^](#_ENREF_18) |
|  |  |  |

| pMG129 | *mtr*A_D56N_ mutant was amplified and cloned into  pJFR19, an integrating vector | [^18^](#_ENREF_18) |
| --- | --- | --- |
| pDS4 | *mtr*A_Y102C_ mutant was amplified and cloned into pJFR19, an integrating vector | [^16^](#_ENREF_16) |
| pDS15a | *mtr*A_Y102C_-*mtr*B was amplified using pMG79 (Fol et al., 2006) as a template and cloned into pGEM-TE vector | This study |
| pDS15 | *mtr*A_Y102C_-*mtr*B was excised from pDS15a as *Nde*I- *Xba*I fragment and cloned into pJFR19, an integrating  vector | This study |
| pDR45 | The upstream region of *M. smegmatis* *mtr*A (1181 bp ) was PCR amplified and cloned into p2Nil vector | This study |
| pDR47 | The downstream region of *M. smegmatis* *mtr*A (1530 bp ) was PCR amplified and cloned into pDR45 | This study |
| pDR49 | The 850 bp *gentamycin* cassette was cloned into pDR47 | This study |
| pDR51 | The marker cassette (6 kb) containing *lac*Z and *sac*B genes amplified from pGOAL17 and cloned into  PDR49 | This study |
| pDR54 | *M. tuberculosis mtr*A under *Ptet* vector | This study |
| pDR55 | The upstream region of *M. tuberculosis* *mtrB* (2145 bp)  cloned into p2Nil vector | This study |
| pDR56 | The downstream region of *M. tuberculosis mtrB* (1566 bp)  cloned into pDR55 | This study |
| pDR57 | The *gentamicin* cassette cloned into pDR56 | This study |
| pDR58 | The 6 kb marker cassette from pGoal vector cloned into  pDR57 | This study |
| pRD73 | *mtrB_TB_-gfp* cloned in pLR52 vector*, mtrB_TB_* CDS replaced *fts*Z in pRD3 | [^16^](#_ENREF_16) |
| pRipA | *ripA* associated MtrA target cloned in pUC57 | This study |
| pSigD | *sigD* associated MtrA target cloned in pUC57 | This study |
| pSigE | *sigE* associated MtrA target cloned in pUC57 | This study |
| PWag31 | *wag31* associated MtrA target cloned in pUC57 | This study |
| pOxyS | *oxyS* associated MtrA target cloned in pUC57 | This study |
| pWhiB3 | *whiB3* associated MtrA target cloned in pUC57 | This study |
| pBetP | *betP* associated MtrA target cloned in pUC57 | This study |
| pRv3887c | *rv3887c* associated MtrA target cloned in pUC57 | This study |
| pRv2526 | *rv2526* associated MtrA target cloned in pUC57 | This study |

The letters in superscripts A, B and C represent MtrA targets that are present unique day3, common to day3 and day 8, and unique to day8 respectively. The letters M and CH represent targets identified by Minch et al.,2015 and Chatterjee et al.,2018, respectively. Target assignments were based on ChiP-Seq peaks located closest to the start codon.

# Table S3 MtrA-Regulon

| **Functional Group** | | **Gene** | | **Synonym** | **Function** | **Distance of ChIP**  **peak from start codon** |
| --- | --- | --- | --- | --- | --- | --- |
| I.A.3 | Fatty acids | *rv0244c* | *A* | *fadE5* | acyl-CoA dehydrogenase | -52 |
| I.A.3 | Fatty acids | *rv0404* | *A, M* | *fadD30* | acyl-CoA synthase | 13 |
| I.A.3 | Fatty acids | *rv0456c* | *A* | *echA2* | Conserved hypothetical protein | -98 |
| I.A.3 | Fatty acids | *rv2486* | *A* | *echA14* | enoyl-CoA hydratase/isomerase  superfamily | -176 |
| I.A.3 | Fatty acids | *rv3826* | *A* | *fadD23* | acyl-CoA synthase | 107 |
| I.A.3 | Fatty acids | *rv1323* | *B* | *fadA4* | acetyl-CoA C-acetyltransferase (aka  thiL) | -41 |
| I.A.3 | Fatty acids | *rv0974c* | *C* | *accD2* | acetyl/propionyl-CoA carboxylase, [beta]  subunit | 31 |
| I.A.3 | Fatty acids | *rv1683* | *C* | *rv1683* | possible acyl-CoA synthase | 180 |
| I.A.3 | Fatty acids | *rv1750c* | *C* | *fadD1* | acyl-CoA synthase | -77 |
| I.B.1 | Glycolysis | *rv1436* | *C* | *gap* | glyceraldehyde 3-phosphate  dehydrogenase | -175 |
| I.B.3 | TCA cycle | *rv0951* | *B* | *sucC* | succinyl-CoA synthase [beta] chain | -162 |
| I.B.6.A aerobic | | *rv3156* | *A* | *nuoL* | NADH dehydrogenase chain L | -197 |
| I.B.6.B anaerobic | | *rv1161* | *A, CH* | *narG* | nitrate reductase [alpha] subunit | -173 |
| I.B.6.B anaerobic | | *rv0252* | *B, M* | *nirB* | nitrite reductase flavoprotein | -44 |
| I.B.6.C Electron transport | | *rv0654* | *B, M* | *rv0654* | putative dioxygenase | 56 |
| I.B.6.C Electron transport | | *rv0247c* | *C* | *rv0247c* | probable iron-sulphur protein | -41 |
| I.B.6.C Electron transport | | *rv1751* | *C* | *rv1751* | possible hydroxylasehyroxylase | 24 |
| I.B.6.C Electron transport | | *rv3230c* | *C* | *rv3230c* | similar to various oxygenases | -11 |
| I.B.6.C Electron transport | | *rv3777* | *C* | *rv3777* | 3-Hydroxyacyl-CoA Dehydrogenase | -306 |

| I.B.7 Miscellaneous oxidoreductases  and oxygenases | *rv0063 ^A, CH^* | *rv0063* | probable oxidoreductase | -98 |
| --- | --- | --- | --- | --- |
| I.B.7 Miscellaneous oxidoreductases  and oxygenases | *rv0245 ^A^* | *rv0245* | probable monooxygenase | -180 |
| I.B.7 Miscellaneous oxidoreductases  and oxygenases | *rv2455c ^A^* | *rv2455c* | probable oxidoreductase alpha subunit | -110 |
| I.B.7 Miscellaneous oxidoreductases  and oxygenases | *rv2893 ^A^* | *rv2893* | similar to alkanal monooxygenase alpha  chain | -282 |
| I.B.7 Miscellaneous oxidoreductases  and oxygenases | *rv3359 ^A^* | *rv3359* | probable oxidoreductase | -56 |
| I.C.5 Sulphur metabolism | *rv3248c ^A^* | *sahH* | adenosylhomocysteinase | 89 |
| I.C.5 Sulphur metabolism | *rv3762c ^C^* | *rv3762c* | probable alkyl sulfatase | -515 |
| I.D.1 Glutamate family | *rv2220 ^B^* | *glnA1* | glutamine synthase class I | 74 |
| I.D.2 Aspartate family | *rv2141c ^C^* | *dapE2* | ArgE/DapE/Acy1/Cpg2/yscS family | -1873 |
| I.F.1 Purine ribonucleotide  biosynthesis | *rv3276c ^A^* | *purK* | Phosphoribosylaminoimidazole  carboxylase ATPase | -38 |
| I.G.1 Biotin | *rv0032 ^A^* | *bioF2* | L-alanine-pimelyl CoA ligase | -424 |
| I.G.13 Cobalamin | *rv0306 ^B^* | *rv0306* | similar to BluB cobalamin synthesis protein R. | -84 |
| I.G.14 Iron utilisation | *rv3841 ^A^* | *bfrB* | bacterioferritin | 120 |
| I.G.4 Molybdopterin | *rv3109 ^A, M^* | *moaA1* | molybdenum cofactor biosynthesis | 134 |
| I.H.1 Synthesis of fatty and mycolic  acids | *rv2524c ^A, CH^* | *fas* | fatty acid synthase | -233 |
| I.H.1 Synthesis of fatty and mycolic  acids | *rv2243 ^C, M^* | *fabD* | malonyl CoA-[ACP] transacylase | -82 |
| I.H.2 Modification of fatty and  mycolic acids | *rv0469 ^A, M^* | *umaA1* | unknown mycolic acid methyltransferase | -21 |
| I.H.2 Modification of fatty and  mycolic acids | *rv0643c ^A, M^* | *mmaA3* | methoxymycolic acid synthase 3 | 96 |
| I.H.2 Modification of fatty and mycolic acids | *rv3229c ^A^* | *desA3* | acyl-[ACP] desaturase | 61 |
| I.H.3 Acyltransferases,  Mycoloyltransferases, and | *rv0129c ^A, M^* | *fbpC2* | antigen 85C | -17 |

| phospholipid synthesis |  |  |  |  |
| --- | --- | --- | --- | --- |
| I.H.3 Acyltransferases,  Mycoloyltransferases, and phospholipid synthesis | *rv1814 ^A^* | *erg3* | possible C-5 sterol desaturase | -30 |
| I.H.3 Acyltransferases, Mycoloyltransferases, and  phospholipid synthesis | *rv2746c ^A^* | *pgsA3* | CDP-diacylglycerol-glycerol-3- phosphate | 25 |
| I.H.3 Acyltransferases, Mycoloyltransferases, and  phospholipid synthesis | *rv1886c ^B, M, CH^* | *fbpB* | antigen 85B, mycolyltransferase | 26 |
| I.I Polyketide and non-ribosomal  peptide synthesis | *rv3825c ^A^* | *pks2* | polyketide synthase | -314 |
| I.I Polyketide and non-ribosomal  peptide synthesis | *rv1013 ^B^* | *pks16* | polyketide synthase | -128 |
| I.I Polyketide and non-ribosomal peptide synthesis | *rv1182 ^C^* | *papA3* | PKS-associated protein, unknown function | -89 |
| I.J.1 Repressors/activators | *rv0212c ^A^* | *nadR* | similar to E.*coli* NadR | -305 |
| I.J.1 Repressors/activators | *rv0328 ^A^* | *rv0328* | transcriptional regulator (TetR/AcrR  family) | 71 |
| I.J.1 Repressors/activators | *rv0452 ^A^* | *rv0452* | putative transcriptional regulator | -5 |
| I.J.1 Repressors/activators | *rv3219 ^A^* | *whiB1* | WhiB transcriptional activator  homologue | -69 |
| I.J.1 Repressors/activators | *rv3295 ^A^* | *rv3295* | transcriptional regulator (TetR/AcrR  family) | -114 |
| I.J.1 Repressors/activators | *rv3583c ^A, M^* | *rv3583c* | putative transcriptional regulator | -92 |
| I.J.1 Repressors/activators | *rv3681c ^A, M^* | *whiB4* | WhiB transcriptional activator  homologue | 55 |
| I.J.1 Repressors/activators | *rv0117 ^B^* | *oxyS* | transcriptional regulator (LysR family) | -138 |
| I.J.1 Repressors/activators | *rv0653c ^B^* | *rv0653c* | putative transcriptional regulator | -15 |
| I.J.1 Repressors/activators | *rv3058c ^B^* | *rv3058c* | putative transcriptional regulator | 11 |
| I.J.1 Repressors/activators | *rv3260c ^B^* | *whiB2* | WhiB transcriptional activator  homologue | -165 |
| I.J.1 Repressors/activators | *rv1359 ^C^* | *rv1359* | putative transcriptional regulator | -125 |

| I.J.1 Repressors/activators | *rv3208A ^C^* | *TB9.4* | conserved hypotheticals | -5 |
| --- | --- | --- | --- | --- |
| I.J.1 Repressors/activators | *rv3416 ^C/B^* | *whiB3* | WhiB transcriptional activator | -97 |
| I.J.2 Two component systems | *rv1033c ^C^* | *trcR* | Two component regulatory protein | 11 |
| I.J.2 Two component systems | *rv0758 ^C^* | *phoR* | Sensor histidine kinase | -308 |
| II.A.1 Ribosomal protein synthesis  and modification | *rv3241c ^A, M^* | *rv3241c* | member of S30AE ribosomal protein  family | -87 |
| II.A.1 Ribosomal protein synthesis  and modification | *rv2412 ^B^* | *rpsT* | 30S ribosomal protein S20 | 10 |
| II.A.1 Ribosomal protein synthesis  and modification | *rv1642 ^C^* | *rpmI* | 50S ribosomal protein L35 | -309 |
| II.A.3 Aminoacyl tRNA synthases and  their modification | *rv0041 ^B^* | *leuS* | leucyl-tRNA synthase | -48 |
| II.A.3 Aminoacyl tRNA synthases and their modification | *rv3674c ^B^* | *nth* | probable endonuclease III | -19 |
| II.A.5 DNA replication, repair, recombination and  restriction/modification | *rv0002 ^A^* | *dnaN* | DNA polymerase III | -1525 |
| II.A.5 DNA replication, repair, recombination and  restriction/modification | *rv3644c ^B, CH^* | *rv3644c* | similar in N-term to DNA polymerase III | 4 |
| II.A.5 DNA replication, repair,  recombination and restriction/modification | *rv0058 ^C^* | *dnaB* | DNA helicase | -138 |
| II.A.7 RNA synthesis, RNA  modification and DNA transcription | *rv3232c ^A^* | *ppk2* | alternative sigma factor for siderophore  production | -57 |
| II.A.7 RNA synthesis, RNA  modification and DNA transcription | *rv1221 ^B^* | *sigE* | ECF subfamily sigma subunit | 127 |
| II.A.7 RNA synthesis, RNA  modification and DNA transcription | *rv3414c ^B^* | *sigD* | ECF subfamily sigma subunit | 54 |
| II.A.7 RNA synthesis, RNA  modification and DNA transcription | *rv0668 ^C^* | *rpoC* | [beta]' subunit of RNA polymerase | 45 |
| II.B.3 Proteins, peptides and glycopeptides | *rv1977 ^A^* | *rv1977* | probable zinc metallopeptidase | -191 |
| II.B.3 Proteins, peptides and  glycopeptides | *rv2457c ^A^* | *clpX* | ATP-dependent Clp protease ATP-  binding subunit ClpX | -70 |

| II.B.3 Proteins, peptides and  glycopeptides | *rv3883c ^A^* | *mycP1* | probable secreted protease | -68 |
| --- | --- | --- | --- | --- |
| II.B.3 Proteins, peptides and  glycopeptides | *rv0319 ^B^* | *pcp* | pyrrolidone-carboxylate peptidase | 11 |
| II.B.4 Polysaccharides, lipopolysaccharides and  phospholipids | *rv3717 ^B^* | *rv3717* | possible N-acetylmuramoyl-L-alanine amidase | -42 |
| II.B.5 Esterases and lipases | *rv2463 ^A^* | *lipP* | probable esterase | -107 |
| II.B.5 Esterases and lipases | *rv2485c ^A^* | *lipQ* | probable carboxlyesterase | -17 |
| II.B.5 Esterases and lipases | *rv2351c ^B^* | *plcA* | phospholipase C precursor | -29 |
| II.C.1 Lipoproteins(lppA-lpr0) | *rv0344c ^A^* | *lpqJ* | Lipoprotein | 7 |
| II.C.1 Lipoproteins(lppA-lpr0) | *rv1690 ^A, M^* | *lprJ* | lipoprotein | -26 |
| II.C.1 Lipoproteins(lppA-lpr0) | *rv1799 ^A^* | *lppT* | probable lipoprotein | -334 |
| II.C.1 Lipoproteins(lppA-lpr0) | *rv3623 ^A^* | *lpqG* | similar OMP28 | -104 |
| II.C.1 Lipoproteins(lppA-lpr0) | *rv0179c ^B, M^* | *lprO* | Lipoprotein | -25 |
| II.C.2 Surface polysaccharides,  lipopolysaccharides, proteins and antigens | *rv1987 ^A, M^* | *rv1987* | probable secreted protein | -27 |
| II.C.2 Surface polysaccharides, lipopolysaccharides, proteins and  antigens | *rv2145c ^A, M^* | *wag31* | antigen 84 (aka wag31) | -112 |
| II.C.2 Surface polysaccharides, lipopolysaccharides, proteins and  antigens | *rv3810 ^A, M^* | *pirG* | cell surface protein precursor (Erp protein) | -87 |
| II.C.3 Murein sacculus and  peptidoglycan | *rv3682 ^A, CH^* | *ponA2* | class A penicillin binding protein | -154 |
| II.C.3 Murein sacculus and  peptidoglycan | *rv3809c ^A^* | *glf* | UDP-galactopyranose mutase | -62 |
| II.C.3 Murein sacculus and  peptidoglycan | *rv2864c ^B, M^* | *rv2864c* | probable penicillin binding protein | -26 |
| II.C.3 Murein sacculus and peptidoglycan | *rv2911 ^B^* | *dacB2* | probable penicillin binding protein | -145 |

| II.C.4 Conserved membrane proteins | *rv0403c ^A^* | *mmpS1* | conserved small membrane protein | -22 |
| --- | --- | --- | --- | --- |
| II.C.4 Conserved membrane proteins | *rv0451c ^A^* | *mmpS4* | conserved small membrane protein | -128 |
| II.C.4 Conserved membrane proteins | *rv1522c ^B^* | *mmpL12* | conserved large membrane protein | 0 |
| II.C.4 Conserved membrane proteins | *rv3823c ^B, M^* | *mmpL8* | conserved large membrane protein | -128 |
| II.C.4 Conserved membrane proteins | *rv0450c ^C^* | *mmpL4* | conserved large membrane protein | -217 |
| II.C.4 Conserved membrane proteins | *rv0402c ^C^* | *mmpL1* | conserved large membrane protein | -292 |
| II.C.5 Other membrane proteins | *rv0345 ^A^* | *rv0345* | unknown possible membrane protein | -8 |
| II.C.5 Other membrane proteins | *rv0888 ^A^* | *rv0888* | possible membrane protein | -95 |
| II.C.5 Other membrane proteins | *rv1004c ^A, M^* | *rv1004c* | possible exported protein | -76 |
| II.C.5 Other membrane proteins | *rv1159 ^A, M^* | *pimE* | probable membrane protein | -19 |
| II.C.5 Other membrane proteins | *rv1490 ^A^* | *rv1490* | unknown putative membrane protein | -97 |
| II.C.5 Other membrane proteins | *rv3887c ^A^* | *eccD2* | probable membrane protein | 221 |
| II.C.5 Other membrane proteins | *rv3901c ^A^* | *rv3901c* | membrane protein TM stretch | 421 |
| II.C.5 Other membrane proteins | *rv0318c ^B^* | *rv0318c* | unknown transmembrane protein | 54 |
| II.C.5 Other membrane proteins | *rv0867c ^B, M, CH^* | *rpfA* | probable exported protein | -313 |
| II.C.5 Other membrane proteins | *rv1433 ^B, M^* | *rv1433* | possible membrane protein | -11 |
| II.C.5 Other membrane proteins | *rv3238c ^B^* | *rv3238c* | unknown, possible membrane protein | -328 |
| II.C.5 Other membrane proteins | *rv3645 ^B, M^* | *rv3645* | probable transmembrane protein | 73 |
| II.C.5 Other membrane proteins | *rv0475 ^C^* | *hbhA* | possible exported protein | -1 |
| II.C.5 Other membrane proteins | *rv1217c ^C^* | *rv1217c* | probable integral membrane protein | -303 |
| II.C.5 Other membrane proteins | *rv1795 ^C^* | *eccD5* | probable membrane protein | -126 |
| III.A.1 Amino acids | *rv0917 ^C^* | *betP* | glycine betaine transport | 59 |
| III.A.2 Cations | *rv1469 ^A, M^* | *ctpD* | probable cadmium-transporting ATPase | -2 |

| III.A.2 Cations | *rv2723 ^B^* | *rv2723* | probable membrane protein, tellurium  resistance | -32 |
| --- | --- | --- | --- | --- |
| III.A.2 Cations | *rv2691 ^C^* | *trkA* | probable potassium uptake protein | -482 |
| III.A.4 Anions | *rv0267 ^A^* | *narU* | similar to nitrite extrusion protein 2 | -18 |
| III.A.4 Anions | *rv0545c ^A^* | *pitA* | low-affinity inorganic phosphate  transporter | 13 |
| III.A.6 Efflux proteins | *rv2846c ^A^* | *efpA* | putative efflux protein | -223 |
| III.B Chaperones/Heat shock | *rv0251c ^B^* | *hsp* | possible heat shock protein | -19 |
| III.B Chaperones/Heat shock | *rv0351 ^B^* | *grpE* | stimulates DnaK ATPase activity | -272 |
| III.D Protein and peptide secretion | *rv2462c ^A^* | *tig* | chaperone protein | -120 |
| III.F Detoxification | *rv1908c ^B, M^* | *katG* | catalase-peroxidase | 35 |
| III.F Detoxification | *rv3171c ^B, CH^* | *hpx* | probable non-heme haloperoxidase | -127 |
| IV.A Virulence | *rv2190c ^A, CH^* | *rv2190c* | putative p60 homologue | -22 |
| IV.A Virulence | *rv1477 ^B, M^* | *ripA* | putative exported p60 protein  homologue | -76 |
| IV.A Virulence | *rv1566c ^B^* | *rv1566c* | putative exported p60 protein  homologue | -87 |
| IV.A Virulence | *rv3500c ^B^* | *yrbE4B* | part of mce4 operon | 0 |
| IV.B.1.A IS6110 | *rv2105 ^C^* | *rv2105* | possible IS6110 transposase | -108 |
| IV.B.1.B IS1081 | *rv1047 ^C^* | *rv1047* | possible IS1081 transposase | -500 |
| IV.B.1.C Others | *rv1036c ^A^* | *rv1036c* | hypothetical protein | 37 |
| IV.B.2 REP13E12 family | *rv0095c ^A^* | *rv0095c* | REP13E12 | 78 |
| IV.B.3 Phage-related functions | *rv1586c ^A, CH^* | *rv1586c* | phiRV1 integrase | 75 |
| IV.C.1.A PE subfamily | *rv0916c ^A^* | *pe7* | PE-family protein | -37 |
| IV.C.1.A PE subfamily | *rv1040c ^A^* | *pe8* | PE-family protein | -100 |
| IV.C.1.A PE subfamily | *rv1088 ^A, CH^* | *pe9* | PE-family protein | -30 |

| IV.C.1.A PE subfamily | *rv3622c ^A^* | *pe32* | PE-family protein | -56 |
| --- | --- | --- | --- | --- |
| IV.C.1.A PE subfamily | *rv1169c ^B^* | *pe11/lipX* | PE-family protein | -2 |
| IV.C.1.A PE subfamily | *rv1788 ^B^* | *pe18* | PE-family protein | 2 |
| IV.C.1.A PE subfamily | *rv1791 ^B, M^* | *pe19* | PE-family protein | -41 |
| IV.C.1.B PE_PGRS subfamily | *rv1468c ^A^* | *pe_pgrs29* | PE_PGRS-family protein | -62 |
| IV.C.1.B PE_PGRS subfamily | *rv1067c ^B^* | *pe_pgrs19* | PE_PGRS-family protein | -39 |
| IV.C.1.B PE_PGRS subfamily | *rv1396c ^B, CH^* | *pe_pgrs25* | PE_PGRS-family protein | -30 |
| IV.C.1.B PE_PGRS subfamily | *rv2162c ^B, M^* | *pe_pgrs38* | PE_PGRS-family protein | -33 |
| IV.C.1.B PE_PGRS subfamily | *rv3345c ^B^* | *pe_pgrs50* | PE_PGRS-family protein | -183 |
| IV.C.1.B PE_PGRS subfamily | *rv0980c ^C^* | *pe_pgrs19* | PE_PGRS-family protein | -8 |
| IV.C.2 PPE family | *rv0096 ^A^* | *ppe1* | PPE-family protein | -94 |
| IV.C.2 PPE family | *rv1706c ^A^* | *ppe23* | PPE-family protein | -86 |
| IV.C.2 PPE family | *rv1787 ^A^* | *ppe25* | PPE-family protein | -66 |
| IV.C.2 PPE family | *rv1802 ^A^* | *ppe30* | PPE-family protein | 21 |
| IV.C.2 PPE family | *rv1918c ^A,M, CH^* | *ppe35* | PPE-family protein | -76 |
| IV.C.2 PPE family | *rv2352c ^A, M, CH^* | *ppe38* | PPE-family protein | -11 |
| IV.C.2 PPE family | *rv2892c ^A^* | *ppe45* | PPE-family protein | -20 |
| IV.C.2 PPE family | *rv0305c ^B^* | *ppe6* | PPE-family protein | 43 |
| IV.C.2 PPE family | *rv1039c ^B^* | *ppe15* | PPE-family protein | 25 |
| IV.C.2 PPE family | *rv3159c ^B^* | *ppe53* | PPE-family protein | 120 |
| IV.C.2 PPE family | *rv1361c ^B, M, CH^* | *ppe19* | PPE-family protein | -40 |
| IV.C.2 PPE family | *rv3136 ^C^* | *ppe51* | PPE-family protein | 43 |

| IV.D Antibiotic production and  resistance | *rv1170 ^B, M^* | *mshb* | similar to S. lincolnensis lmbE | 4 |
| --- | --- | --- | --- | --- |
| IV.D Antibiotic production and  resistance | *rv3290c ^B^* | *lat* | lysine-[epsilon] aminotransferase | 112 |
| IV.F Cytochrome P450 enzymes | *rv0327c ^A^* | *cyp135A1* | cytochrome P-450  monooxygenasemonoxygenase | -38 |
| IV.F Cytochrome P450 enzymes | *rv3059 ^A^* | *cyp136* | possible lanosterol 14-alpha-  demethylases | 21 |
| IV.F Cytochrome P450 enzymes | *rv1676 ^C^* | *rv1676* | possible cytochrome P450 | -227 |
| IV.H Miscellaneous transferases | *rv0188 ^A, M^* | *rv0188* | putative methyltransferase | 72 |
| IV.H Miscellaneous transferases | *rv2294 ^A^* | *rv2294* | aminotransferase | -57 |
| IV.H Miscellaneous transferases | *rv3699 ^A^* | *rv3699* | Probable methyltransferase | -552 |
| IV.H Miscellaneous transferases | *rv0187 ^B^* | *rv0187* | probable o-methyltransferase | -642 |
| IV.H Miscellaneous transferases | *rv1523 ^B, M, CH^* | *rv1523* | possible methyl-sterol transferase | 100 |
| IV.H Miscellaneous transferases | *rv2958c ^B^* | *rv2958c* | similar to variety of glycosyltransferases | -103 |
| IV.H Miscellaneous transferases | *rv1220c ^B^* | *rv1220c* | probable methyltransferase | -245 |
| IV.H Miscellaneous transferases | *rv1220c ^B^* | *rv1220c* | probable methyltransferase | -245 |
| IV.H Miscellaneous transferases | *rv1373 ^C^* | *rv1373* | slight similarity to sulfotransferases | -291 |
| V Conserved hypotheticals | *rv0130 ^A^* | *htdZ* | conserved hypothetical protein | -98 |
| V Conserved hypotheticals | *rv0208c ^A, M^* | *rv208c* | conserved hypothetical protein | 28 |
| V Conserved hypotheticals | *rv0266c ^A^* | *oplA* | conserved hypothetical protein | -48 |
| V Conserved hypotheticals | *rv0312 ^A^* | *rv0312* | conserved hypothetical protein | -15 |
| V Conserved hypotheticals | *rv1038c ^A^* | *esxJ* | conserved hypothetical protein | -15 |
| V Conserved hypotheticals | *rv1158c ^A^* | *rv1158c* | conserved hypothetical protein | -13 |
| V Conserved hypotheticals | *rv1215c ^A^* | *rv1215c* | conserved hypothetical protein | 1 |
| V Conserved hypotheticals | *rv1321 ^A^* | *rv1321* | conserved hypothetical protein | -236 |

| V Conserved hypotheticals | *rv1793 ^A^* | *esxN* | conserved hypothetical protein | 9 |
| --- | --- | --- | --- | --- |
| V Conserved hypotheticals | *rv1830 ^A^* | *rv1830* | conserved hypothetical protein | -77 |
| V Conserved hypotheticals | *rv1951c ^A^* | *rv1951c* | conserved hypothetical protein | -173 |
| V Conserved hypotheticals | *rv1952 ^A^* | *vapB14* | conserved hypothetical protein | 63 |
| V Conserved hypotheticals | *rv1996 ^A^* | *rv1996* | conserved hypothetical protein | -177 |
| V Conserved hypotheticals | *rv2458 ^A^* | *mmuM* | conserved hypothetical protein | -127 |
| V Conserved hypotheticals | *rv2493 ^A^* | *vapB38* | conserved hypothetical protein | -306 |
| V Conserved hypotheticals | *rv2631 ^A^* | *rv2631* | conserved hypothetical protein | -10 |
| V Conserved hypotheticals | *rv2747 ^A^* | *argA* | conserved hypothetical protein | 54 |
| V Conserved hypotheticals | *rv2758c ^A, M^* | *vapB21* | conserved hypothetical protein | 72 |
| V Conserved hypotheticals | *rv3353c ^A, CH^* | *rv3353c* | conserved hypothetical protein | 8 |
| V Conserved hypotheticals | *rv3354 ^A, M^* | *rv3354c* | conserved hypothetical protein | 4 |
| V Conserved hypotheticals | *rv3488 ^A^* | *rv3488* | conserved hypothetical protein | -86 |
| V Conserved hypotheticals | *rv3697c/A ^A^* | *vapB/C48* | conserved hypothetical protein | 38 |
| V Conserved hypotheticals | *rv3866 ^A^* | *espG1* | conserved hypothetical protein | 180 |
| V Conserved hypotheticals | *rv3888c ^A^* | *rv3888c* | conserved hypothetical protein | 88 |
| V Conserved hypotheticals | *rv0004 ^B^* | *rv0004* | conserved hypothetical protein | -469 |
| V Conserved hypotheticals | *rv0116c ^B, M, CH^* | *ldtA* | conserved hypothetical protein | 61 |
| V Conserved hypotheticals | *rv0207c ^B^* | *rv0207c* | conserved hypothetical protein | 67 |
| V Conserved hypotheticals | *rv0320 ^B, M^* | *rv0320* | conserved hypothetical protein | 25 |
| V Conserved hypotheticals | *rv0937c ^B^* | *mku* | conserved hypothetical protein | 33 |
| V Conserved hypotheticals | *rv0938 ^B^* | *ligD* | conserved hypothetical protein | 25 |
| V Conserved hypotheticals | *rv0950c ^B, M, CH^* | *rv0950c* | conserved hypothetical protein | 30 |

| V Conserved hypotheticals | *rv1291c ^B, M^* | *rv1291c* | conserved hypothetical protein | -8 |
| --- | --- | --- | --- | --- |
| V Conserved hypotheticals | *rv1435c ^B^* | *rv1435c* | conserved hypothetical protein | 7 |
| V Conserved hypotheticals | *rv1754c ^B, M^* | *rv1754c* | conserved hypothetical protein | 123 |
| V Conserved hypotheticals | *rv1815 ^B, M, CH^* | *rv1815* | conserved hypothetical protein | -28 |
| V Conserved hypotheticals | *rv1884c ^B, M, CH^* | *rpfC* | conserved hypothetical protein | 116 |
| V Conserved hypotheticals | *rv2411c ^B^* | *rv2411c* | conserved hypothetical protein | 6 |
| V Conserved hypotheticals | *rv2417c ^B^* | *rv2417c* | conserved hypothetical protein | -174 |
| V Conserved hypotheticals | *rv2721c ^B^* | *rv2721c* | conserved hypothetical protein | -115 |
| V Conserved hypotheticals | *rv3209 ^B^* | *rv3209* | conserved hypothetical protein | 201 |
| V Conserved hypotheticals | *rv3261 ^B^* | *fbiA* | conserved hypothetical protein | -93 |
| V Conserved hypotheticals | *rv3547 ^B^* | *ddn* | conserved hypothetical protein | 85 |
| V Conserved hypotheticals | *rv3716c ^B, M^* | *rv3716c* | conserved hypothetical protein | 46 |
| V Conserved hypotheticals | *rv3787c ^B^* | *rv3787c* | conserved hypothetical protein | -47 |
| V Conserved hypotheticals | *rv3867 ^B^* | *espH* | conserved hypothetical protein | -24 |
| V Conserved hypotheticals | *rv0040c ^B, M^* | *mtc28* | conserved hypothetical protein | -21 |
| V Conserved hypotheticals | *rv1009 ^B, M, CH^* | *rpfB* | conserved hypothetical protein | -15 |
| V Conserved hypotheticals | *rv2865 ^B, CH^* | *relB2* | conserved hypothetical protein | -80 |
| V Conserved hypotheticals | *rv2450c ^B^* | *rpfE* | conserved hypothetical protein | -295 |
| V Conserved hypotheticals | *rv2143 ^C^* | *rv2143* | conserved hypothetical protein | -544 |
| V Conserved hypotheticals | *rv0047c ^C^* | *rv0047c* | conserved hypothetical protein | -11 |
| V Conserved hypotheticals | *rv0074 ^C^* | *rv0074* | conserved hypothetical protein | -710 |
| V Conserved hypotheticals | *rv1269c ^C^* | *rv1269c* | conserved hypothetical protein | -51 |
| V Conserved hypotheticals | *rv2053c ^C^* | *rv2053c* | conserved hypothetical protein | -32 |

| V Conserved hypotheticals | *rv2699c ^C^* | *rv2699c* | conserved hypothetical protein | -13 |
| --- | --- | --- | --- | --- |
| V Conserved hypotheticals | *rv2700 ^C^* | *rv2700* | conserved hypothetical protein | -89 |
| V Conserved hypotheticals | *rv3415c ^C^* | *rv3415c* | conserved hypothetical protein | -101 |
| V Conserved hypotheticals | *rv2389c- ^C^*  *rv2390c* | *rpfD* | Resuscitation promoting factor | -52 |
| V Conserved hypotheticals | *rv0365c ^C^* | *rv0365c* | conserved hypothetical protein | -7 |
| V Conserved hypotheticals | *rv1354c ^C^* | *rv1354c* | conserved hypothetical protein | 86 |
| V Conserved hypotheticals | *rv1362c ^C^* | *rv1362c* | conserved hypothetical protein | 65 |
| V Conserved hypotheticals | *rv1425 ^C^* | *rv1425* | conserved hypothetical protein | -351 |
| V Conserved hypotheticals | *rv2166 ^C^* | *rv2166* | conserved hypothetical protein | -16 |
| V Conserved hypotheticals | *rv2372c ^C^* | *rv2372c* | conserved hypothetical protein | -715 |
| V Conserved hypotheticals | *rv2526 ^C^* | *vapB17* | conserved hypothetical protein | 58 |
| V Conserved hypotheticals | *rv2638 ^C^* | *rv2638* | conserved hypothetical protein | 2 |
| V Conserved hypotheticals | *rv2742c ^C^* | *rv2742c* | conserved hypothetical protein | -193 |
| VI Unknowns | *rv0209 ^A^* | *rv0209* | hypothetical protein | -17 |
| VI Unknowns | *rv0257 ^A^* | *rv0257* | hypothetical protein | -727 |
| VI Unknowns | *rv0887c ^A^* | *rv0887c* | hypothetical protein | -112 |
| VI Unknowns | *rv1115 ^A^* | *rv1115* | hypothetical protein | 27 |
| VI Unknowns | *rv1116 ^A, M, CH^* | *rv1116* | hypothetical protein | 10 |
| VI Unknowns | *rv1265 ^A^* | *rv1265* | hypothetical protein | 88 |
| VI Unknowns | *rv1507c ^A^* | *rv1507c* | hypothetical protein | 134 |
| VI Unknowns | *rv1976c ^A^* | *rv1976c* | hypothetical protein | -156 |
| VI Unknowns | *rv2293c ^A, M^* | *rv2293c* | hypothetical protein | -69 |
| VI Unknowns | *rv2336 ^A^* | *rv2336* | hypothetical protein | 20 |

| VI Unknowns | *rv2517c ^A^* | *rv2517c* | hypothetical protein | 65 |
| --- | --- | --- | --- | --- |
| VI Unknowns | *rv2949c ^A, CH^* | *rv2949c* | hypothetical protein | 97 |
| VI Unknowns | *rv3294 ^A^* | *rv3294c* | hypothetical protein | 109 |
| VI Unknowns | *rv3528c ^A^* | *rv3528c* | hypothetical protein | 37 |
| VI Unknowns | *rv3698 ^A^* | *rv3698* | hypothetical protein | 70 |
| VI Unknowns | *rv0309 ^B, M^* | *rv0309* | hypothetical protein | -14 |
| VI Unknowns | *rv0790c ^B^* | *rv0790c* | hypothetical protein | 57 |
| VI Unknowns | *rv1268c ^B, M, CH^* | *rv1268c* | hypothetical protein | 3 |
| VI Unknowns | *rv1772 ^B, M^* | *rv1772* | hypothetical protein | -74 |
| VI Unknowns | *rv2147c ^B^* | *rv2147c* | hypothetical protein | 80 |
| VI Unknowns | *rv3256c ^B^* | *rv3256c* | hypothetical protein | -171 |
| VI Unknowns | *rv3788 ^B^* | *rv3788* | hypothetical protein | -7 |
| VI Unknowns | *rv3857c ^B, M, CH^* | *rv3857c* | hypothetical protein | -37 |
| VI Unknowns | *rv2342 ^B^* | *rv2342* | hypothetical protein | -58 |
| VI Unknowns | *rv3675 ^B, M^* | *rv3675* | hypothetical protein | 147 |
| VI Unknowns | *rv1322A ^B^* | *rv1322A* | hypothetical protein | 74 |
| VI Unknowns | *rv0508 ^C^* | *rv0508* | hypothetical protein | -227 |
| VI Unknowns | *rv2929 ^C^* | *rv2929* | hypothetical protein | -97 |
| VI Unknowns | *rv0996 ^C, M^* | *rv0996* | hypothetical protein | -59 |
| VI Unknowns | *rv1887 ^C^* | *rv1887* | hypothetical protein | -147 |
| VI Unknowns | *rv2054 ^C^* | *rv2054* | hypothetical protein | 35 |
| VI Unknowns | *rv2525c ^C^* | *rv2525c* | hypothetical protein | -9 |
| VI Unknowns | *rv1000c ^C^* | *rv1000c* | hypothetical protein | -482 |

| VI Unknowns | *rv1914c ^C^* | *rv1914c* | hypothetical protein | -8 |
| --- | --- | --- | --- | --- |
| VI Unknowns | *rv2219A ^C^* | *rv2219A* | hypothetical protein | -132 |
| VI Unknowns | *rv2274c ^C^* | *mazF8* | hypothetical protein | -212 |
| VI Unknowns | *rv3258c ^C^* | *rv3258c* | hypothetical protein | 48 |
| VI Unknowns | *rv3259 ^C^* | *rv3259* | hypothetical protein | -77 |

*Note:* The MtrA-regulon includes not only genes essential for survival in chemically defined media, e.g., *rip*A*, whiB2* (potential regulator of CD)*, gap* (glycolysis)*, suc*C (TCA cycle), *des*A3 (mycolic acid synthesis), *pgs*A3 (phospholipid synthesis), etc., but also those which are necessary for full virulence, persistence and the expansion of cell walls, e.g., *whi*B3, *sig*E, *sig*D, *fbp*B, *fbp*C, *acc*D2, *fad*A4, *mmp*S4, *mmp*L4 and *lpr*J, but are not necessary for survival in nutrient broth.

**References**

1 Cole, S. T. *et al.* Deciphering the biology of Mycobacterium tuberculosis from the complete genome sequence. *Nature* **393**, 537-+, doi:Doi 10.1038/31159 (1998).

2 Kana, B. D. *et al.* The resuscitation-promoting factors of Mycobacterium tuberculosis are required for virulence and resuscitation from dormancy but are collectively dispensable for growth in vitro. *Mol Microbiol* **67**, 672-684, doi:10.1111/j.1365-2958.2007.06078.x (2008).

3 Mukamolova, G. V. *et al.* Muralytic activity of Micrococcus luteus Rpf and its relationship to physiological activity in promoting bacterial growth and resuscitation. *Mol Microbiol* **59**, 84-98, doi:10.1111/j.1365-2958.2005.04930.x (2006).

4 Mukamolova, G. V. *et al.* A family of autocrine growth factors in Mycobacterium tuberculosis. *Mol Microbiol* **46**, 623-635, doi:DOI 10.1046/j.1365-2958.2002.03184.x (2002).

5 Russell-Goldman, E., Xu, J. Y., Wang, X. B., Chan, J. & Tufariello, J. M. A Mycobacterium tuberculosis Rpf double-knockout strain exhibits profound defects in reactivation from chronic tuberculosis and innate immunity phenotypes. *Infect Immun* **76**, 4269-4281, doi:10.1128/Iai.01735-07 (2008).

6 Tufariello, J. M. *et al.* Deletion of the Mycobacterium tuberculosis resuscitation-promoting factor Rv1009 gene results in delayed reactivation from chronic tuberculosis. *Infect Immun* **74**, 2985-2995, doi:10.1128/Iai.74.5.2985-2995.2006 (2006).

7 Tufariello, J. M., Jacobs, W. R. & Chan, J. Individual Mycobacterium tuberculosis resuscitation-promoting factor homologues are dispensable for growth in vitro and in vivo. *Infect Immun* **72**, 515-526, doi:10.1128/Iai.72.1.515-526.2004 (2004).

8 Alam, M. S., Garg, S. K. & Agrawal, P. Studies on structural and functional divergence among seven WhiB proteins of Mycobacterium tuberculosis H37Rv. *Febs J* **276**, 76-93, doi:10.1111/j.1742-4658.2008.06755.x (2009).

9 Geiman, D. E., Raghunand, T. R., Agarwal, N. & Bishai, W. R. Differential gene expression in response to exposure to antimycobacterial agents and other stress conditions among seven Mycobacterium tuberculosis whiB-like genes. *Antimicrob Agents Ch* **50**, 2836-2841, doi:10.1128/Aac.00295-06 (2006).

10 Singh, A. *et al.* Mycobacterium tuberculosis WhiB3 Maintains Redox Homeostasis by Regulating Virulence Lipid Anabolism to Modulate Macrophage Response. *Plos Pathog* **5**, doi:ARTN e100054510.1371/journal.ppat.1000545 (2009).

11 Rodrigue, S., Provvedi, R., Jacques, P. E., Gaudreau, L. & Manganelli, R. The sigma factors of Mycobacterium tuberculosis. *Fems Microbiol Rev* **30**, 926-941, doi:10.1111/j.1574-6976.2006.00040.x (2006).

12 Calamita, H. *et al.* The Mycobacterium tuberculosis SigD sigma factor controls the expression of ribosome-associated gene products in stationary phase and is required for full virulence. *Cell Microbiol* **7**, 233-244, doi:10.1111/j.1462-5822.2004.00454.x (2005).

13 Raman, S., Hazra, R., Dascher, C. C. & Husson, R. N. Transcription regulation by the Mycobacterium tuberculosis alternative sigma factor SigD and its role in virulence. *J Bacteriol* **186**, 6605-6616, doi:10.1128/Jb.186.19.6605-6616.2004 (2004).

14 He, H. J., Hovey, R., Kane, J., Singh, V. & Zahrt, T. C. MprAB is a stress-responsive two-component system that directly regulates expression of sigma factors SigB and SigE in Mycobacterium tuberculosis. *J Bacteriol* **188**, 2134-2143, doi:10.1128/Jb.188.6.2134-2143.2006 (2006).

15 Song, T., Song, S. E., Raman, S., Anaya, M. & Husson, R. N. Critical role of a single position in the-35 element for promoter recognition by Mycobacterium tuberculosis SigE and SigH. *J Bacteriol* **190**, 2227-2230, doi:10.1128/Jb.01642-07 (2008).

16 Plocinska, R. *et al.* Septal Localization of the Mycobacterium tuberculosis MtrB Sensor Kinase Promotes MtrA Regulon Expression. *J Biol Chem* **287**, 23887-23899, doi:10.1074/jbc.M112.346544 (2012).

17 Nair, N. *et al.* Synchronous replication initiation in novel Mycobacterium tuberculosis dnaA cold-sensitive mutants. *Mol Microbiol* **71**, 291-304, doi:10.1111/j.1365-2958.2008.06523.x (2009).

18 Fol, M. *et al.* Modulation of Mycobacterium tuberculosis proliferation by MtrA, an essential two-component response regulator. *Mol Microbiol* **60**, 643-657, doi:10.1111/j.1365-2958.2006.05137.x (2006).

19 Darwin, K. H., Ehrt, S., Gutierrez-Ramos, J. C., Weich, N. & Nathan, C. F. The proteasome of Mycobacterium tuberculosis is required for resistance to nitric oxide. *Science* **302**, 1963-1966, doi:DOI 10.1126/science.1091176 (2003).

20 Dziedzic, R. *et al.* Mycobacterium tuberculosis ClpX Interacts with FtsZ and Interferes with FtsZ Assembly. *Plos One* **5**, doi:ARTN e1105810.1371/journal.pone.0011058 (2010).

21 Parish, T. & Stoker, N. G. Use of a flexible cassette method to generate a double unmarked Mycobacterium tuberculosis tlyA plcABC mutant by gene replacement. *Microbiol-Uk* **146**, 1969-1975, doi:Doi 10.1099/00221287-146-8-1969 (2000).
