# Supplementary material for: MtrA Response Regulator Controls Cell Division and Cell Wall Metabolism and Affects Susceptibility of Mycobacteria to the First Line Antituberculosis Drugs
Source: Front Microbiol. 2018 Nov 23;9:2839. doi: 10.3389/fmicb.2018.02839 (PMC6265350; doi:10.3389/fmicb.2018.02839)
Supplement: Supplementary file 2 [file Data_Sheet_2.PDF]

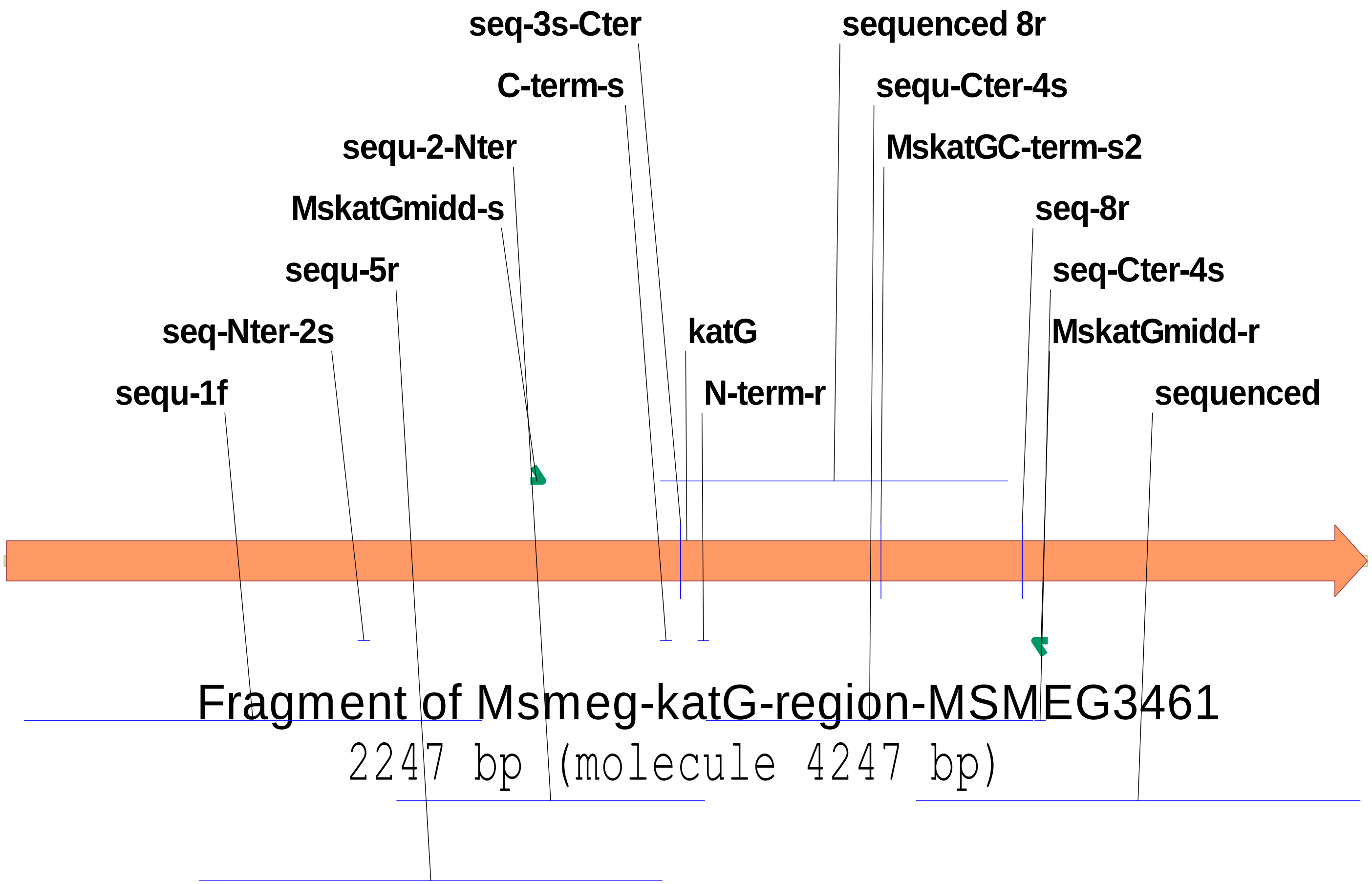


Blue lines represent the individualy sequenced fragments of *msmeg_3461*. The sequencing primers are marked above the ORF.

Untitled

```
                                                                    Section 1
                    (1)  1        10        20        30              49
Msmeg-katG-coding   (1)  -------------------------------------------------
  5-10-katGNter6R   (1)  TGGACGGCCCCTTGATCACAGCCCACATCACCAACCACCCGGAAGGATT
                                                                    Section 2
                   (50)  50       60        70        80              98
Msmeg-katG-coding   (1)  -----GTGTCATCCGATACATCTGACAGCCGCCCACCCAACCCGGATAC
  5-10-katGNter6R  (50)  TCGCTGTGTCATCCGATACATCTGACAGCCGCCCACCCAACCCGGATAC
                                                                    Section 3
                   (99)  99        110       120       130           147
Msmeg-katG-coding  (45)  GAAGACCGCGAGCACGAGCGAGAGCGAAAACCCCGCGATCCCTTCCCCG
  5-10-katGNter6R  (99)  GAAGACCGCGAGCACGAGCGAGAGCGAAAACCCCGCGATCCCTTCCCCG
                                                                    Section 4
                  (148)  148        160       170       180          196
Msmeg-katG-coding  (94)  AAGCCGAAATCGGGCGCTCCGCTTCGCAACCAGGACTGGTGGCCCAACC
  5-10-katGNter6R (148)  AAGCCGAAATCGGGCGCTCCGCTTCGCAACCAGGACTGGTGGCCCAACC
                                                                    Section 5
                  (197)  197         210       220       230         245
Msmeg-katG-coding (143)  AGATCGACGTATCACGCCTGCATCCGCATCCCCCGCAGGGCAACCCGCT
  5-10-katGNter6R (197)  AGATCGACGTATCACGCCTGCATCCGCATCCCCCGCAGGGCAACCCGCT
                                                                    Section 6
                  (246)  246          260       270       280        294
Msmeg-katG-coding (192)  GGGCGAAGACTTCGACTACGCCGAGGAATTCGCCAAGCTCGACGTGAAC
  5-10-katGNter6R (246)  GGGCGAAGACTTCGACTACGCCGAGGAATTCGCCAAGCTCGACGTGAAC
                                                                    Section 7
                  (295)  295  300       310       320       330       343
Msmeg-katG-coding (241)  GCGCTCAAGGCCGACCTCACGGCGCTGATGACGCAGTCCCAGGACTGGT
  5-10-katGNter6R (295)  GCGCTCAAGGCCGACCTCACGGCGCTGATGACGCAGTCCCAGGACTGGT
                                                                    Section 8
                  (344)  344   350       360       370       380      392
Msmeg-katG-coding (290)  GGCCCGCCGACTACGGGCACTACGGCGGCCTGTTCATCCGTATGAGCTG
  5-10-katGNter6R (344)  GGCCCGCCGACTACGGGCACTACGGCGGCCTGTTCATCCGTATGAGCTG
                                                                    Section 9
                  (393)  393    400       410       420       430     441
Msmeg-katG-coding (339)  GCACTCGGCAGGCACCTACCGCATCCATGACGGCCGCGGGGGCGGCGGG
  5-10-katGNter6R (393)  GCACTCGGCAGGCACCTACCGCATCCATGACGGCCGCGGGGGCGGCGGG
```

```
                                                                  Section 10
                  (442) 442     450       460       470       480      490
Msmeg-katG-coding (388) CAGGGCGCCCAACGGTTCGCACCGATCAACTCGTGGCCCGACAACGTCA
  5-10-katGNter6R (442) CAGGGCGCCCAACGGTTCGCACCGATCAACTCGTGGCCCGACAACGTCA
                                                                  Section 11
                  (491) 491      500       510       520               539
Msmeg-katG-coding (437) GCCTGGACAAGGCCAGAAGACTGTTGTGGCCCATCAAGCAG----AAGT
  5-10-katGNter6R (491) GCCTGGACAAGGCCAGAAGACTGTTGTGGCCCATCA-GCAGTCTTAATA
                                                                  Section 12
                  (540) 540       550       560       570              588
Msmeg-katG-coding (482) ACGGCAACAAGATCTCCTGGGCCGATCTTCTGGTGTTCACCGGCAACGT
  5-10-katGNter6R (539) ACCGCCTAKAGGTTT----------------------------------
                                                                  Section 13
                  (589) 589        600       610       620             637
Msmeg-katG-coding (531) GGCTTTGGAATCCATGGGCTTCAAGACGTTTGGCTTCGGCTTCGGCCGC
  5-10-katGNter6R (554) -------------------------------------------------
                                                                  Section 14
                  (638) 638         650       660       670            686
Msmeg-katG-coding (580) GAGGACATCTGGGAGCCAGAGGAGATCCTGTTCGGCGAGGAAGACGAAT
  5-10-katGNter6R (554) -------------------------------------------------
                                                                  Section 15
                  (687) 687          700       710       720           735
Msmeg-katG-coding (629) GGCTGGGCACCGACAAGCGGTACGGCGGCGGCGAGCAGCGCCAACTCGC
  5-10-katGNter6R (554) -------------------------------------------------
                                                                  Section 16
                  (736) 736           750       760       770          784
Msmeg-katG-coding (678) CGAACCGTACGGTGCGACCACGATGGGCCTGATCTACGTCAACCCCGAA
  5-10-katGNter6R (554) -------------------------------------------------
                                                                  Section 17
                  (785) 785  790       800       810       820         833
Msmeg-katG-coding (727) GGCCCGGAGGGTCAACCCGACCCGCTCGCCGCGGCACACGACATCCGCG
  5-10-katGNter6R (554) -------------------------------------------------
                                                                  Section 18
                  (834) 834   840       850       860       870        882
Msmeg-katG-coding (776) AGACGTTCGGCCGCATGGCGATGAACGACGAGGAGACCGCGGCGCTGAT
  5-10-katGNter6R (554) -------------------------------------------------
```

Section 1

```
                    (1)  1       10        20        30              49
Msmeg-katG-coding   (1)  GTGTCATCCGATACATCTGACAGCCGCCCACCCAACCCGGATACGAAGA
5-10-katGNter1      (1)  --------------GCGGGGCCCGCCGCCACCCAACCCGGATACGAAGA
5-10-katGNter2      (1)  -------------------------------------------------
5-10-katGNter5R     (1)  ---------------------------------------------ARTC
```

Section 2

```
                    (50)  50        60        70        80              98
Msmeg-katG-coding   (50)  CCGCGAGCACGAGCGAGAGCGAAAACCCCGCGATCCCTTCCCCGAAGCC
5-10-katGNter1      (36)  CCGCGAGCACGAGCGAGAGCGAAAACCCCGCGATCCCTTCCCCGAAGCC
5-10-katGNter2      (1)   -------------------------------------------------
5-10-katGNter5R     (5)   GAGCGGGAMCGA-CGAGA-CGAA---CCCGCGATCCTT---CCGA-GCC
```

Section 3

```
                    (99)  99         110       120       130            147
Msmeg-katG-coding   (99)  GAAATCGGGCGCTCCGCTTCGCAACCAGGACTGGTGGCCCAACCAGATC
5-10-katGNter1      (85)  RAAATCGGGCGCTCCGCTTCGCAACCAGGACTGGTGGCCCAACCAGATC
5-10-katGNter2      (1)   -------------------------------------------------
5-10-katGNter5R     (45)  GAA-TCGT-CGTTC-GCTG-GCAACA--GACTGATAGCC--AACAGATC
```

Section 4

```
                    (148)  148        160       170       180           196
Msmeg-katG-coding   (148)  GACGTATCACGCCTGCATCCGCATCCCCCGCAGGGCAACCCGCTGGGCG
5-10-katGNter1      (134)  GACGTATCACGCCTGCATCCGCATCCCCCGCAGGGCAACCCGCTGGGCG
5-10-katGNter2      (1)    -------------------------------------------------
5-10-katGNter5R     (86)   GACGTATCACGC-TGCATTCGCATCCCC-GCAGACCATCC-GCTGRGCG
```

Section 5

```
                    (197)  197         210       220       230          245
Msmeg-katG-coding   (197)  AA-GACTTCGACTACGCCGAGGAATTCGCCAAGCTCGACGTGAACGCGC
5-10-katGNter1      (183)  AA-GACTTCGACTACGCCGAGGAATTCGCCAAGCTCGACGTGAACGCGC
5-10-katGNter2      (1)    -------------------------------------------------
5-10-katGNter5R     (132)  AAAGACTTCGACTACGCCGAAGAATTCGCCAAGCTCGACGTGAACGCGC
```

Section 6

```
                    (246)  246           260       270       280        294
Msmeg-katG-coding   (245)  TCAAGG-CCGACCTCACGGCGCTGATGACGCAGTCCCA-GGACTGGTGG
5-10-katGNter1      (231)  TCAAGG-CCGACCTCACGGCGCTGATGACGCAGTCCCA-GGACTGGTGG
5-10-katGNter2      (1)    -------------------------------------------------
5-10-katGNter5R     (181)  TCAAAGACCGACCTCACGGCGCTGATGACGCAGTCCCAAGGACTGT--G
```

Section 7

```
                    (295)  295  300       310       320       330        343
Msmeg-katG-coding   (292)  CCCGCCGACTACGGGCACTACGGCGGCCTGTTCATCCGTATGAGCTGGC
5-10-katGNter1      (278)  CCCGCCGACTACGGGCACTACGGCGGCCTGTTCATCCGTATGAGCTGGC
5-10-katGNter2      (1)    -------------------------------------------------
5-10-katGNter5R     (228)  TCCGC-GACTACRRGCACTACRGCRGCCTGTTCATCCGTATGAGCTGGC
```

Section 8

```
                           344   350                 360                 370                 380             392
Msmeg-katG-coding  (341)  ACTCGGCAGGCACCTACCGCATCCATGACGGCCGCGGGGCGGCGGGCA
5-10-katGNter1     (327)  ACTCGGCAGGCACCTACCGCATCCATGACGGCCGCGGGGCGGCGGGCA
5-10-katGNter2       (1)  -------------------------------------------------
5-10-katGNter5R    (276)  ACTCGGCAGGCACCTACCGCATCCATGACGGCCGCGGGGCGGCGGGCA
```

Section 9

```
                           393     400                 410                 420                 430           441
Msmeg-katG-coding  (390)  GGGCGCCCAACGGTTCGCACCGATCAACTCGTGGCCCGACAACGTCAGC
5-10-katGNter1     (376)  GGGCGCCCAACGGTTCGCACCGATCAACTCGTGGCCCGACAACGTCAGC
5-10-katGNter2       (1)  -------------------------------------------------
5-10-katGNter5R    (325)  GRGCGCCCAACGGTTCGCACCGATCAACTCGTGGCCCGACAACGTCAGC
```

Section 10

```
                           442       450                 460                 470                 480         490
Msmeg-katG-coding  (439)  CTGGACAAGGCCAGAAGACTGTTGTGGCCCATCAAGCAGAAGTACGGCA
5-10-katGNter1     (425)  CTGGACAAGGCCAGAAGACTGTTGTGGCCCATCAAGCAGAAGTACGGCA
5-10-katGNter2       (1)  -------------------------------------------------
5-10-katGNter5R    (374)  CTGGACAAGGCCAGAAGACTGTTGTGGCCCATCAAGCAGAAGTACGGCA
```

Section 11

```
                           491         500                 510                 520                       539
Msmeg-katG-coding  (488)  ACAAGATCTCCTGGGCCGATCTTCTGGTGTTCACCGGCAACGTGGCTTT
5-10-katGNter1     (474)  ACAAGATCTCCTGGGCCGATCTTCTGGTGTTCACCGGCAACGTGGCTTT
5-10-katGNter2       (1)  -------------------------------------------------
5-10-katGNter5R    (423)  ACAAGATCTCCTGGGCCGATCTTCTGGTGTTCACCGGCAACGTGGCTTT
```

Section 12

```
                           540       550                 560                 570                         588
Msmeg-katG-coding  (537)  GGAATCCATGGGCTTCAAGACGTTTGGCTTCGGCTTCGGCCGCGAGGAC
5-10-katGNter1     (523)  GGAATCCATGGGCTTCAAGACGTTTGGCTTCGGCTTCGGCCGCGAGGAC
5-10-katGNter2       (1)  -------------------------------------------------
5-10-katGNter5R    (472)  GGAATCCATGGGCTTCAAGACGTTTGGCTTCGGCTTCGGCCGCGAGGAC
```

Section 13

```
                           589         600                 610                 620                       637
Msmeg-katG-coding  (586)  ATCTGGGAGCCAGAGGAGATCCTGTTCGGCGAGGAAGACGA-ATGGCTG
5-10-katGNter1     (572)  ATCTGGGAGCCAGAGGAGATCCTGTTCGGCGAGGAAGACGA-ATGGCTG
5-10-katGNter2       (1)  -------------------------ACGGTAACKGSGGTGCGATGGCTG
5-10-katGNter5R    (521)  ATCTGGGAGCCAGAGGAGATCCTGTTCGGCGAGGAAGACGA-ATGGCTG
```

Section 14

```
                           638         650                 660                 670                       686
Msmeg-katG-coding  (634)  GGCACCGACAAGCGGTACGGCGGCGGCGAGCAGCGCCAACTCGCCGAAC
5-10-katGNter1     (620)  GGCACCGACAAGCGGTACGGCGGCGGCGAGCAGCGCCAACTCGCCGAAC
5-10-katGNter2      (25)  GGCACCGACA-GCGGTACGGCGGCGGCGAGCAGCGCCAACTCGCCGAAC
5-10-katGNter5R    (569)  GGCACCGACAAGCGGTACGGCGGCGGCGAGCAGCGCCAACTCGCCGAAC
```

```
                                                                          Section 15
                         (687) 687          700       710       720            735
      Msmeg-katG-coding  (683) CGTACGGTGCGACCACGATGGGCCTGATCTACGTCAACCCCGAAGGCCC
        5-10-katGNter1   (669) CGTACGGTGCGACCACGATGGGCCTGATCTACGTCAACCCCGAAGGCCC
        5-10-katGNter2    (73) CGTACGGTGCGACCACGATGGGCCTGATCTACGTCAACCCCGAAGGCCC
        5-10-katGNter5R  (618) CGTACGGTGCGACCACGATGGGCCTGATCTACGTCAACCCCGAAGGCCC
                                                                          Section 16
                         (736) 736           750       760       770           784
      Msmeg-katG-coding  (732) GGAGGGTCAACCCGACCCGCTCGCCGCGGCACACGACATCCGCGAGACG
        5-10-katGNter1   (718) GGAGGGTCAACCCGACCCGCTCGCCGCGGCACACGACATCCGCGAGACG
        5-10-katGNter2   (122) GGAGGGTCAACCCGACCCGCTCGCCGCGGCACACGACATCCGCGAGACG
        5-10-katGNter5R  (667) GGAGGGTCAACCCGACCCGCTCGCCGCGGCACACGACATCCGCGAGACG
                                                                          Section 17
                         (785) 785  790       800       810       820          833
      Msmeg-katG-coding  (781) TTCGGCCGCATGGCGATGAACGACGAGGAGACCGCGGCGCTGATCGTCG
        5-10-katGNter1   (767) TTCGGGCGCATGGCGATGAACGACGAGGAGACCGCG-CGCTGATCGTCG
        5-10-katGNter2   (171) TTCGGCCGCATGGCGATGAACGACGAGGAGACCGCGGCGCTGATCGTCG
        5-10-katGNter5R  (716) TTCGGCCGCATGGCGATGAACGACGAGGAGACCGCGGCGCTGATCGTCG
                                                                          Section 18
                         (834) 834   840       850       860       870         882
      Msmeg-katG-coding  (830) GCGGGCACACGTTCGGCAAGACCCACGGCGCCGGCGATGCCAGCCTCGT
        5-10-katGNter1   (815) C--GGCACACGTTCGGCAAGACCCACGGCGC-GGCGATGC-AGCYTCGT
        5-10-katGNter2   (220) GCGGGCACACGTTCGGCAAGACCCACGGCGCCGGCGATGCCAGCCTCGT
        5-10-katGNter5R  (765) GCGGGCACACGTTCGGCAAGACCCACGGCGCCGGCGATGCCAGCCTCGT
                                                                          Section 19
                         (883) 883    890       900       910       920        931
      Msmeg-katG-coding  (879) CGGTCCCGAGCCCGAGGCCGCACCGATCGAGCAGCAGGGTCTCGGCTGG
        5-10-katGNter1   (860) CGGTCC-GAGCYCGAGTC-GCAC-GATCGAGCAGCAAGA-CTCG-CTG-
        5-10-katGNter2   (269) CGGTCCCGAGCCCGAGGCCGCACCGATCGAGCAGCAGGGTCTCGGCTGG
        5-10-katGNter5R  (814) CGGTCCCGAGCCCGAGGCCGCACCGATCGAGCAGCAGGGTCTCGGCTGG
                                                                          Section 20
                         (932) 932     940       950       960       970       980
      Msmeg-katG-coding  (928) AAGAGTTCCTACGGCACGGGCAAGGGTCCCGACACCATCACCAGCGGGC
        5-10-katGNter1   (903) -AGAGTTC-TACG-CACGG-CAA--GTTCCGAC----TCATCACAGCGC
        5-10-katGNter2   (318) AAGAGTTCCTACGGCACGGGCAAGGGTCCCGACACCATCACCAGCGGGC
        5-10-katGNter5R  (863) AAGAGTTCCTACGGCACGGGCAAGGGTCCCGACACCATCACCAGCGGGC
                                                                          Section 21
                         (981) 981      990       1000      1010              1029
      Msmeg-katG-coding  (977) TGGAGGTCGTGTGGACCAACACACCGACCAAGTGGGACAACAGCTTCCT
        5-10-katGNter1   (942) TG-AGATCGTGTG----A-CAACTCGAC--AGTG--ACA-CAGCT--CT
        5-10-katGNter2   (367) TGGAGGTCGTGTGGACCAACACACCGACCAAGTGGGACAACAGCTTCCT
        5-10-katGNter5R  (912) TGGAGGTCGTGTGGACCAACACACCGACCAAGTGGGACAACAGCTTCCT
```

```
                                                                      Section 22
                   (1030) 1030      1040      1050      1060              1078
Msmeg-katG-coding  (1026) GGAGATCCTCTACGGCTACGAGTGGGAGCTGACCAAGAGCCCGGCCGGC
5-10-katGNter1      (978) GA--ATCCT-TACG-CTAC-AGATGAAGCTGACTA-GAGC---------
5-10-katGNter2      (416) GGAGATCCTCTACGGCTACGAGTGGGAGCTGACCAAGAGCCCGGCCGGC
5-10-katGNter5R     (961) GGAGATCCTCTACGGCTACGAGTGGGAGCTGACCAAGAGCCCGGCCGGC
                                                                      Section 23
                   (1079) 1079       1090      1100      1110             1127
Msmeg-katG-coding  (1075) GCCTGGCAGTTCACCGCGAAGGACGGGGCAGGCGCGGGCACCATCCCGG
5-10-katGNter1     (1012) -------------------------------------------------
5-10-katGNter2      (465) GCCTGGCAGTTCACCGCGAAGGACGGGGCAGGCGCGGGCACCATCCCGG
5-10-katGNter5R    (1010) GCCTGGCAGT-CACCGCGAARMAAAWAC---------------------
                                                                      Section 24
                   (1128) 1128        1140      1150      1160            1176
Msmeg-katG-coding  (1124) ATCCGTTCGGCGGGCCAGGACGCAACCCGACCATGCTGGTCACCGACAT
5-10-katGNter1     (1012) -------------------------------------------------
5-10-katGNter2      (514) ATCCGTTCGGCGGGCCAGGACGCAACCCGAMCCATGCTA----------
5-10-katGNter5R    (1037) -------------------------------------------------
                                                                      Section 25
                   (1177) 1177         1190      1200      1210           1225
Msmeg-katG-coding  (1173) CTCGATGCGCGTGGACCCGATCTACGGGAAGATCACGCGCCGGTGGCTC
5-10-katGNter1     (1012) -------------------------------------------------
5-10-katGNter2      (553) -------------------------------------------------
5-10-katGNter5R    (1037) -------------------------------------------------
                                                                      Section 26
                   (1226) 1226          1240      1250      1260          1274
Msmeg-katG-coding  (1222) GACCATCCCGAGGAGCTCTCGGAGGCCTTCGCGAAGGCCTGGTACAAGC
5-10-katGNter1     (1012) -------------------------------------------------
5-10-katGNter2      (553) -------------------------------------------------
5-10-katGNter5R    (1037) -------------------------------------------------
                                                                      Section 27
                   (1275) 1275 1280      1290      1300      1310         1323
Msmeg-katG-coding  (1271) TGCTGCACCGCGACATGGGACCGATCTCGCGGTATCTGGGGCCCTGGGT
5-10-katGNter1     (1012) -------------------------------------------------
5-10-katGNter2      (553) -------------------------------------------------
5-10-katGNter5R    (1037) -------------------------------------------------
                                                                      Section 28
                   (1324) 1324  1330      1340      1350      1360        1372
Msmeg-katG-coding  (1320) CGCCGAACCGCAGCTGTGGCAGGACCCGGTCCCGGCGGTCGATCATCCG
5-10-katGNter1     (1012) -------------------------------------------------
5-10-katGNter2      (553) -------------------------------------------------
5-10-katGNter5R    (1037) -------------------------------------------------
```

Section 25

```
                    (1153) 1153   1160                1170                1180                1190             1200
Msmeg-katG-coding   (1153) ACCATGCTGGTCACCGACATCTCGATGCGCGTGGACCCGATCTACGGG
5-10-katG-Cterm-1-8R  (1)  ------------------------------------------------
5-10-katG-Cterm-3s   (12)  MCAATGCTGGTCACCGACATCTCGATGCGCGTGGACCCGATCTACGGG
```

Section 26

```
                    (1201) 1201              1210                1220                1230                  1248
Msmeg-katG-coding   (1201) AAGATCACGCGCCGGTGGCTCGACCATCCCGAGGAGCTCTCGGAGGCC
5-10-katG-Cterm-1-8R  (1)  ------------------------------------------------
5-10-katG-Cterm-3s   (60)  AAGATCACGCGCCGGTGGCTCGACCATCCCGAGGAGCTCTCGGAGGCC
```

Section 27

```
                    (1249) 1249                   1260                1270                1280             1296
Msmeg-katG-coding   (1249) TTCGCGAAGGCCTGGTACAAGCTGCTGCACCGCGACATGGGACCGATC
5-10-katG-Cterm-1-8R  (1)  ------------------------------------------------
5-10-katG-Cterm-3s  (108)  TTCGCGAAGGCCTGGTACAAGCTGCTGCACCGCGACATGGGACCGATC
```

Section 28

```
                    (1297) 1297                        1310                1320                1330        1344
Msmeg-katG-coding   (1297) TCGCGGTATCTGGGGCCCTGGGTCGCCGAACCGCAGCTGTGGCAGGAC
5-10-katG-Cterm-1-8R  (1)  ------------------------------------------------
5-10-katG-Cterm-3s  (156)  TCGCGGTATCTGGGGCCCTGGGTCGCCGAACCGCAGCTGTGGCAGGAC
```

Section 29

```
                    (1345) 1345  1350                1360                1370                1380         1392
Msmeg-katG-coding   (1345) CCGGTCCCGGCGGTCGATCATCCGCTGGTCGACGACCAGGACATCGCC
5-10-katG-Cterm-1-8R  (1)  ------------------------------------------------
5-10-katG-Cterm-3s  (204)  CCGGTCCCGGCGGTCGATCATCCGCTGGTCGACGACCAGGACATCGCC
```

Section 30

```
                    (1393) 1393   1400                1410                1420                1430             1440
Msmeg-katG-coding   (1393) GCGCTGAAGTCCACGGTGCTCGATTCCGGGCTGTCGACGGGACAACTG
5-10-katG-Cterm-1-8R  (1)  ------------------------------------------------
5-10-katG-Cterm-3s  (252)  GCGCTGAAGTCCACGGTGCTCGATTCCGGGCTGTCGACGGGACAACTG
```

Section 31

```
                    (1441) 1441              1450                1460                1470                  1488
Msmeg-katG-coding   (1441) ATCAAGACTGCGTGGGCGTCGGCGGCGAGCTACCGCAACACCGACAAG
5-10-katG-Cterm-1-8R  (1)  ----AGACTGCGTGGGCGTCGGCGGCGAGCTACCGCAACACCGACAAG
5-10-katG-Cterm-3s  (300)  ATCAAGACTGCGTGGGCGTCGGCGGCGAGCTACCGCAACACCGACAAG
```

Section 32

```
                    (1489) 1489                   1500                1510                1520             1536
Msmeg-katG-coding   (1489) CGCGGCGGCGCCAACGGGGCCCGGGTGCGTCTGGAACCGCAGAAGAAC
5-10-katG-Cterm-1-8R (45)  CGCGGCGGCGCCAACGGGGCCCGGGTGCGTCTGGAACCGCAGAAGAAC
5-10-katG-Cterm-3s  (348)  CGCGGCGGCGCCAACGGGGCCCGGGTGCGTCTGGAACCGCAGAAGAAC
```

Section 33

| | | |
|---|---|---|
| | (1537) | 1537 1550 1560 1570 1584 |
| Msmeg-katG-coding | (1537) | TGGGACGTCAACGAACCGGCCGAACTGGCCACGGTGCTGCCGGTGCTC |
| 5-10-katG-Cterm-1-8R | (93) | TGGGACGTCAACGAACCGGCCGAACTGGCCACGGTGCTGCCGGTGCTC |
| 5-10-katG-Cterm-3s | (396) | TGGGACGTCAACGAACCGGCCGAACTGGCCACGGTGCTGCCGGTGCTC |

Section 34

| | | |
|---|---|---|
| | (1585) | 1585 1590 1600 1610 1620 1632 |
| Msmeg-katG-coding | (1585) | GAGCGGATCCAGCAGGACTTCAACGCCTCGGCATCCGGCGGCAAGAAG |
| 5-10-katG-Cterm-1-8R | (141) | GAGCGGATCCAGCAGGACTTCAACGCCTCGGCATCCGGCGGCAAGAAG |
| 5-10-katG-Cterm-3s | (444) | GAGCGGATCCAGCAGGACTTCAACGCCTCGGCATCCGGCGGCAAGAAG |

Section 35

| | | |
|---|---|---|
| | (1633) | 1633 1640 1650 1660 1670 1680 |
| Msmeg-katG-coding | (1633) | GTCTCGTTGGCCGACCTGATCGTGCTGGCCGGTTCGGCGGCGATCGAG |
| 5-10-katG-Cterm-1-8R | (189) | GTCTCGTTGGCCGACCTGATCKKTWWRYKA------------------ |
| 5-10-katG-Cterm-3s | (492) | GTCTCGTTGGCCGACCTGATCGTGCTGGCCGGTTCGGCGGCGATCGAG |

Section 36

| | | |
|---|---|---|
| | (1681) | 1681 1690 1700 1710 1728 |
| Msmeg-katG-coding | (1681) | AAGGCCGCCAAGGACGGTGGCTACAACGTCACGGTGCCGTTCGCACCG |
| 5-10-katG-Cterm-1-8R | (219) | ------------------------------------------------ |
| 5-10-katG-Cterm-3s | (540) | AAGGCCGCCAAGGACA-------------------------------- |

Section 37

| | | |
|---|---|---|
| | (1729) | 1729 1740 1750 1760 1776 |
| Msmeg-katG-coding | (1729) | GGACGCACGGACGCGAGCCAGGAGAACACCGACGTGGAGTCCTTCGCG |
| 5-10-katG-Cterm-1-8R | (219) | ------------------------------------------------ |
| 5-10-katG-Cterm-3s | (556) | ------------------------------------------------ |

Section 38

| | | |
|---|---|---|
| | (1777) | 1777 1790 1800 1810 1824 |
| Msmeg-katG-coding | (1777) | GTGCTCGAGCCGCGGGCCGACGGGTTCCGCAACTACGTCCGCCCGGGT |
| 5-10-katG-Cterm-1-8R | (219) | ------------------------------------------------ |
| 5-10-katG-Cterm-3s | (556) | ------------------------------------------------ |

Section 39

| | | |
|---|---|---|
| | (1825) | 1825 1830 1840 1850 1860 1872 |
| Msmeg-katG-coding | (1825) | GAGAAGGTCCAGCTGGAGAAGATGCTGCTCGAACGGGCGTACTTCCTG |
| 5-10-katG-Cterm-1-8R | (219) | ------------------------------------------------ |
| 5-10-katG-Cterm-3s | (556) | ------------------------------------------------ |

Section 40

| | | |
|---|---|---|
| | (1873) | 1873 1880 1890 1900 1910 1920 |
| Msmeg-katG-coding | (1873) | GGCGTGACCGCACCGCAGCTGACGGCTCTGGTCGGCGGGTTGCGCGCG |
| 5-10-katG-Cterm-1-8R | (219) | ------------------------------------------------ |
| 5-10-katG-Cterm-3s | (556) | ------------------------------------------------ |

```
                                                                      Section 41
                        (1921) 1921      1930      1940      1950              1968
     Msmeg-katG-coding (1921) CTGGACGTCAACCACGGCGGCACCAAACACGGTGTGTTCACCGACCGG
    5-10-katG-Cterm-1-8R (219) ------------------------------------------------
     5-10-katG-Cterm-3s (556) ------------------------------------------------
                                                                      Section 42
                        (1969) 1969       1980      1990      2000            2016
     Msmeg-katG-coding (1969) CCGGGCGCTTTGACCAACGACTTCTTCGTCAACCTGCTCGACATGGGC
    5-10-katG-Cterm-1-8R (219) ------------------------------------------------
     5-10-katG-Cterm-3s (556) ------------------------------------------------
                                                                      Section 43
                        (2017) 2017         2030      2040      2050          2064
     Msmeg-katG-coding (2017) ACCGAGTGGAAGACCTCGGAGACGACGGAGAACGTCTACGAGGGAGTC
    5-10-katG-Cterm-1-8R (219) ------------------------------------------------
     5-10-katG-Cterm-3s (556) ------------------------------------------------
                                                                      Section 44
                        (2065) 2065 2070      2080      2090      2100        2112
     Msmeg-katG-coding (2065) GATCGCAAGACCGGACAGTTGAAGTGGACCGCGACCGCGAATGACCTT
    5-10-katG-Cterm-1-8R (219) ------------------------------------------------
     5-10-katG-Cterm-3s (556) ------------------------------------------------
                                                                      Section 45
                        (2113) 2113   2120      2130      2140      2150      2160
     Msmeg-katG-coding (2113) GTGTTCGGGTCACATTCGGTGCTGCGTGCCGTGGCCGAGGTTTATGCC
    5-10-katG-Cterm-1-8R (219) ------------------------------------------------
     5-10-katG-Cterm-3s (556) ------------------------------------------------
                                                                      Section 46
                        (2161) 2161     2170      2180      2190              2208
     Msmeg-katG-coding (2161) CAGTCCGACAACGGCGAACGGTTCGTCAACGACTTCGTCAAGGCCTGG
    5-10-katG-Cterm-1-8R (219) ------------------------------------------------
     5-10-katG-Cterm-3s (556) ------------------------------------------------
                                                                      Section 47
                        (2209) 2209       2220      2230          2247
     Msmeg-katG-coding (2209) GTCAAGGTCATGAACAACGACCGGTTCGACCTCAAGTAA
    5-10-katG-Cterm-1-8R (219) ---------------------------------------
     5-10-katG-Cterm-3s (556) ---------------------------------------
```

```
                                                                              Section 19
                         (883) 883       890        900        910        920          931
Msmeg-katG-coding        (883) CCCGAGCCCGAGGCCGCACCGATCGAGCAGCAGGGTCTCGGCTGGAAGA
5-10-katG-Cmidd            (1) -------------------------------------------------
                                                                              Section 20
                         (932) 932        940        950        960        970         980
Msmeg-katG-coding        (932) GTTCCTACGGCACGGGCAAGGGTCCCGACACCATCACCAGCGGGCTGGA
5-10-katG-Cmidd            (1) -------------------------------------------------
                                                                              Section 21
                         (981) 981         990        1000       1010              1029
Msmeg-katG-coding        (981) GGTCGTGTGGACCAACACACCGACCAAGTGGGACAACAGCTTCCTGGAG
5-10-katG-Cmidd            (1) -------------------------------------------------
                                                                              Section 22
                        (1030) 1030        1040       1050       1060              1078
Msmeg-katG-coding       (1030) ATCCTCTACGGCTACGAGTGGGAGCTGACCAAGAGCCCGGCCGGCGCCT
5-10-katG-Cmidd            (1) -------------------------------------------------
                                                                              Section 23
                        (1079) 1079         1090       1100       1110             1127
Msmeg-katG-coding       (1079) GGCAGTTCACCGCGAAGGACGGGGCAGGCGCGGGCACCATCCCGGATCC
5-10-katG-Cmidd            (1) -------------------------------------------------
                                                                              Section 24
                        (1128) 1128          1140       1150       1160            1176
Msmeg-katG-coding       (1128) GTTCGGCGGGCCAGGACGCAACCCGACCATGCTGGTCACCGACATCTCG
5-10-katG-Cmidd            (1) -------------------------------------------------
                                                                              Section 25
                        (1177) 1177           1190       1200       1210           1225
Msmeg-katG-coding       (1177) ATGCGCGTGGACCCGATCTACGGGAAGATCACGCGCCGGTGGCTCGACC
5-10-katG-Cmidd            (1) -------------------------------------------------
                                                                              Section 26
                        (1226) 1226            1240       1250       1260          1274
Msmeg-katG-coding       (1226) ATCCCGAGGAGCTCTCGGAGGCCTTCGCGAAGGCCTGGTACAAGCTGCT
5-10-katG-Cmidd            (1) -------------------------------------------------
                                                                              Section 27
                        (1275) 1275 1280      1290       1300       1310           1323
Msmeg-katG-coding       (1275) GCACCGCGACATGGGACCGATCTCGCGGTATCTGGGGCCCTGGGTCGCC
5-10-katG-Cmidd            (1) ---------CATGGGCWCGATCTKCGCGTATCTGGGGCCCTGGGTCGCC
```

```
                                                                     Section 28
             (1324) 1324  1330                1340                1350                1360              1372
Msmeg-katG-coding (1324) GAACCGCAGCTGTGGCAGGACCCGGTCCCGGCGGTCGATCATCCGCTGG
 5-10-katG-Cmidd    (41) GAACCGCAGCTGTGGCAGGACCCGGTCCCGGCGGTCGATCATCCGCTGG
                                                                     Section 29
             (1373) 1373    1380                1390                1400                1410            1421
Msmeg-katG-coding (1373) TCGACGACCAGGACATCGCCGCGCTGAAGTCCACGGTGCTCGATTCCGG
 5-10-katG-Cmidd    (90) TCGACGACCAGGACATCGCCGCGCTGAAGTCCACGGTGCTCGATTCCGG
                                                                     Section 30
             (1422) 1422      1430                1440                1450                1460          1470
Msmeg-katG-coding (1422) GCTGTCGACGGGACAACTGATCAAGACTGCGTGGGCGTCGGCGGCGAGC
 5-10-katG-Cmidd   (139) GCTGTCGACGGGACAACTGATCAAGACTGCGTGGGCGTCGGCGGCGAGC
                                                                     Section 31
             (1471) 1471        1480                1490                1500                            1519
Msmeg-katG-coding (1471) TACCGCAACACCGACAAGCGCGGCGGCGCCAACGGGGCCCGGGTGCGTC
 5-10-katG-Cmidd   (188) TACCGCAACACCGACAAGCGCGGCGGCGCCAACGGGGCCCGGGTGCGTC
                                                                     Section 32
             (1520) 1520          1530                1540                1550                          1568
Msmeg-katG-coding (1520) TGGAACCGCAGAAGAACTGGGACGTCAACGAACCGGCCGAACTGGCCAC
 5-10-katG-Cmidd   (237) TGGAACCGCAGAAGAACTGGGACGTCAACGAACCGGCCGAACTGGCCAC
                                                                     Section 33
             (1569) 1569            1580                1590                1600                        1617
Msmeg-katG-coding (1569) GGTGCTGCCGGTGCTCGAGCGGATCCAGCAGGACTTCAACGCCTCGGCA
 5-10-katG-Cmidd   (286) GGTGCTGCCGGTGCTCGAGCGGATCCAGCAGGACTTCAACGCCTCGGCA
                                                                     Section 34
             (1618) 1618              1630                1640                1650                      1666
Msmeg-katG-coding (1618) TCCGGCGGCAAGAAGGTCTCGTTGGCCGACCTGATCGTGCTGGCCGGTT
 5-10-katG-Cmidd   (335) TCCGGCGGCAAGAAGGTCTCGTTGGCCGACCTGATCGTGCTGGCCGGTT
                                                                     Section 35
             (1667) 1667                1680                1690                1700                    1715
Msmeg-katG-coding (1667) CGGCGGCGATCGAGAAGGCCGCCAAGGACGGTGGCTACAACGTCACGGT
 5-10-katG-Cmidd   (384) CGGCGGCGATCGAGAAGGCCGCCAAGGACGGTGGCTACAACGTCACGGT
                                                                     Section 36
             (1716) 1716                  1730                1740                1750                  1764
Msmeg-katG-coding (1716) GCCGTTCGCACCGGGACGCACGGACGCGAGCCAGGAGAACACCGACGTG
 5-10-katG-Cmidd   (433) GCCGTTCGCACCGGGACGCACGGACGCGAGCCAGGAGAACACCGACGTG
```

```
                                                                   Section 37
                   (1765) 1765 1770      1780      1790      1800          1813
Msmeg-katG-coding (1765) GAGTCCTTCGCGGTGCTCGAGCCGCGGGCCGACGGGTTCCGCAACTACG
     5-10-katG-Cmidd (482) GAGTCCTTCGCGGTGCTCGAGCCGCGGGCC-------------------
                                                                   Section 38
                   (1814) 1814  1820      1830      1840      1850        1862
Msmeg-katG-coding (1814) TCCGCCCGGGTGAGAAGGTCCAGCTGGAGAAGATGCTGCTCGAACGGGC
     5-10-katG-Cmidd (512) -------------------------------------------------
                                                                   Section 39
                   (1863) 1863   1870      1880      1890      1900       1911
Msmeg-katG-coding (1863) GTACTTCCTGGGCGTGACCGCACCGCAGCTGACGGCTCTGGTCGGCGGG
     5-10-katG-Cmidd (512) -------------------------------------------------
                                                                   Section 40
                   (1912) 1912    1920      1930      1940      1950      1960
Msmeg-katG-coding (1912) TTGCGCGCTGGACGTCAACCACGGCGGCACCAAACACGGTGTGTTCA
     5-10-katG-Cmidd (512) -------------------------------------------------
                                                                   Section 41
                   (1961) 1961     1970      1980      1990               2009
Msmeg-katG-coding (1961) CCGACCGGCCGGGCGCTTTGACCAACGACTTCTTCGTCAACCTGCTCGA
     5-10-katG-Cmidd (512) -------------------------------------------------
                                                                   Section 42
                   (2010) 2010      2020      2030      2040              2058
Msmeg-katG-coding (2010) CATGGGCACCGAGTGGAAGACCTCGGAGACGACGGAGAACGTCTACGAG
     5-10-katG-Cmidd (512) -------------------------------------------------
                                                                   Section 43
                   (2059) 2059       2070      2080      2090             2107
Msmeg-katG-coding (2059) GGAGTCGATCGCAAGACCGGACAGTTGAAGTGGACCGCGACCGCGAATG
     5-10-katG-Cmidd (512) -------------------------------------------------
                                                                   Section 44
                   (2108) 2108        2120      2130      2140            2156
Msmeg-katG-coding (2108) ACCTTGTGTTCGGGTCACATTCGGTGCTGCGTGCCGTGGCCGAGGTTTA
     5-10-katG-Cmidd (512) -------------------------------------------------
                                                                   Section 45
                   (2157) 2157         2170      2180      2190           2205
Msmeg-katG-coding (2157) TGCCCAGTCCGACAACGGCGAACGGTTCGTCAACGACTTCGTCAAGGCC
     5-10-katG-Cmidd (512) -------------------------------------------------
```

```
                                                                          Section 1
                    (1)  1       10        20        30        40        51
  Msmeg-katG-coding (1)  GTGTCATCCGATACATCTGACAGCCGCCCACCCAACCCGGATACGAAGACC
      9-06-2-4fkatG (1)  ---------------------------------------------------
   9-06-4fCter-katG (1)  ---------------------------------------------------
                                                                          Section 2
                   (52)  52     60        70        80        90        102
  Msmeg-katG-coding (52) GCGAGCACGAGCGAGAGCGAAAACCCCGCGATCCCTTCCCCGAAGCCGAAA
      9-06-2-4fkatG (1)  ---------------------------------------------------
   9-06-4fCter-katG (1)  ---------------------------------------------------
                                                                          Section 3
                  (103)  103    110       120       130       140       153
  Msmeg-katG-coding (103) TCGGGCGCTCCGCTTCGCAACCAGGACTGGTGGCCCAACCAGATCGACGTA
      9-06-2-4fkatG (1)  ---------------------------------------------------
   9-06-4fCter-katG (1)  ---------------------------------------------------
                                                                          Section 4
                  (154)  154   160       170       180       190       204
  Msmeg-katG-coding (154) TCACGCCTGCATCCGCATCCCCCGCAGGGCAACCCGCTGGGCGAAGACTTC
      9-06-2-4fkatG (1)  ---------------------------------------------------
   9-06-4fCter-katG (1)  ---------------------------------------------------
                                                                          Section 5
                  (205)  205  210       220       230       240        255
  Msmeg-katG-coding (205) GACTACGCCGAGGAATTCGCCAAGCTCGACGTGAACGCGCTCAAGGCCGAC
      9-06-2-4fkatG (1)  ---------------------------------------------------
   9-06-4fCter-katG (1)  ---------------------------------------------------
                                                                          Section 6
                  (256)  256           270       280       290           306
  Msmeg-katG-coding (256) CTCACGGCGCTGATGACGCAGTCCCAGGACTGGTGGCCCGCCGACTACGGG
      9-06-2-4fkatG (1)  ---------------------------------------------------
   9-06-4fCter-katG (1)  ---------------------------------------------------
                                                                          Section 7
                  (307)  307          320       330       340            357
  Msmeg-katG-coding (307) CACTACGGCGGCCTGTTCATCCGTATGAGCTGGCACTCGGCAGGCACCTAC
      9-06-2-4fkatG (1)  ---------------------------------------------------
   9-06-4fCter-katG (1)  ---------------------------------------------------
```

```
                                                                    Section 8
                    (358) 358         370       380       390                 408
Msmeg-katG-coding   (358) CGCATCCATGACGGCCGCGGGGGCGGCGGGCAGGGCGCCCAACGGTTCGCA
9-06-2-4fkatG         (1) ---------------------------------------------------
9-06-4fCter-katG      (1) ---------------------------------------------------
                                                                    Section 9
                    (409) 409        420       430       440                  459
Msmeg-katG-coding   (409) CCGATCAACTCGTGGCCCGACAACGTCAGCCTGGACAAGGCCAGAAGACTG
9-06-2-4fkatG         (1) ---------------------------------------------------
9-06-4fCter-katG      (1) ---------------------------------------------------
                                                                   Section 10
                    (460) 460       470       480       490       500       510
Msmeg-katG-coding   (460) TTGTGGCCCATCAAGCAGAAGTACGGCAACAAGATCTCCTGGGCCGATCTT
9-06-2-4fkatG         (1) ---------------------------------------------------
9-06-4fCter-katG      (1) ---------------------------------------------------
                                                                   Section 11
                    (511) 511      520       530       540       550        561
Msmeg-katG-coding   (511) CTGGTGTTCACCGGCAACGTGGCTTTGGAATCCATGGGCTTCAAGACGTTT
9-06-2-4fkatG         (1) ---------------------------------------------------
9-06-4fCter-katG      (1) ---------------------------------------------------
                                                                   Section 12
                    (562) 562     570       580       590       600         612
Msmeg-katG-coding   (562) GGCTTCGGCTTCGGCCGCGAGGACATCTGGGAGCCAGAGGAGATCCTGTTC
9-06-2-4fkatG         (1) ---------------------------------------------------
9-06-4fCter-katG      (1) ---------------------------------------------------
                                                                   Section 13
                    (613) 613    620       630       640       650          663
Msmeg-katG-coding   (613) GGCGAGGAAGACGAATGGCTGGGCACCGACAAGCGGTACGGCGGCGGCGAG
9-06-2-4fkatG         (1) ---------------------------------------------------
9-06-4fCter-katG      (1) ---------------------------------------------------
                                                                   Section 14
                    (664) 664   670       680       690       700           714
Msmeg-katG-coding   (664) CAGCGCCAACTCGCCGAACCGTACGGTGCGACCACGATGGGCCTGATCTAC
9-06-2-4fkatG         (1) ---------------------------------------------------
9-06-4fCter-katG      (1) ---------------------------------------------------
```

```
                                                            Section 15
                    (715) 715  720       730       740       750            765
Msmeg-katG-coding   (715) GTCAACCCCGAAGGCCCGGAGGGTCAACCCGACCCGCTCGCCGCGGCACAC
9-06-2-4fkatG         (1) ---------------------------------------------------
9-06-4fCter-katG      (1) ---------------------------------------------------
                                                            Section 16
                    (766) 766           780       790       800             816
Msmeg-katG-coding   (766) GACATCCGCGAGACGTTCGGCCGCATGGCGATGAACGACGAGGAGACCGCG
9-06-2-4fkatG         (1) ---------------------------------------------------
9-06-4fCter-katG      (1) ---------------------------------------------------
                                                            Section 17
                    (817) 817          830       840       850              867
Msmeg-katG-coding   (817) GCGCTGATCGTCGGCGGGCACACGTTCGGCAAGACCCACGGCGCCGGCGAT
9-06-2-4fkatG         (1) ---------------------------------------------------
9-06-4fCter-katG      (1) ---------------------------------------------------
                                                            Section 18
                    (868) 868         880       890       900               918
Msmeg-katG-coding   (868) GCCAGCCTCGTCGGTCCCGAGCCCGAGGCCGCACCGATCGAGCAGCAGGGT
9-06-2-4fkatG         (1) ---------------------------------------------------
9-06-4fCter-katG      (1) ---------------------------------------------------
                                                            Section 19
                    (919) 919        930       940       950                969
Msmeg-katG-coding   (919) CTCGGCTGGAAGAGTTCCTACGGCACGGGCAAGGGTCCCGACACCATCACC
9-06-2-4fkatG         (1) ---------------------------------------------------
9-06-4fCter-katG      (1) ---------------------------------------------------
                                                            Section 20
                    (970) 970       980       990       1000      1010     1020
Msmeg-katG-coding   (970) AGCGGGCTGGAGGTCGTGTGGACCAACACACCGACCAAGTGGGACAACAGC
9-06-2-4fkatG         (1) ---------------------------------------------------
9-06-4fCter-katG      (1) ---------------------------------------------------
                                                            Section 21
                   (1021) 1021     1030      1040      1050      1060      1071
Msmeg-katG-coding  (1021) TTCCTGGAGATCCTCTACGGCTACGAGTGGGAGCTGACCAAGAGCCCGGCC
9-06-2-4fkatG         (1) ---------------------------------------------------
9-06-4fCter-katG      (1) ---------------------------------------------------
```

```
                                                                          Section 22
                   (1072) 1072    1080      1090      1100      1110           1122
Msmeg-katG-coding  (1072) GGCGCCTGGCAGTTCACCGCGAAGGACGGGGCAGGCGCGGGCACCATCCCG
9-06-2-4fkatG         (1) ---------------------------------------------------
9-06-4fCter-katG      (1) ---------------------------------------------------
                                                                          Section 23
                   (1123) 1123   1130      1140      1150      1160            1173
Msmeg-katG-coding  (1123) GATCCGTTCGGCGGGCCAGGACGCAACCCGACCATGCTGGTCACCGACATC
9-06-2-4fkatG         (1) ---------------------------------------------------
9-06-4fCter-katG      (1) ---------------------------------------------------
                                                                          Section 24
                   (1174) 1174  1180      1190      1200      1210             1224
Msmeg-katG-coding  (1174) TCGATGCGCGTGGACCCGATCTACGGGAAGATCACGCGCCGGTGGCTCGAC
9-06-2-4fkatG         (1) ---------------------------------------------------
9-06-4fCter-katG      (1) ---------------------------------------------------
                                                                          Section 25
                   (1225) 1225 1230      1240      1250      1260              1275
Msmeg-katG-coding  (1225) CATCCCGAGGAGCTCTCGGAGGCCTTCGCGAAGGCCTGGTACAAGCTGCTG
9-06-2-4fkatG         (1) ---------------------------------------------------
9-06-4fCter-katG      (1) ---------------------------------------------------
                                                                          Section 26
                   (1276) 1276          1290      1300      1310               1326
Msmeg-katG-coding  (1276) CACCGCGACATGGGACCGATCTCGCGGTATCTGGGGCCCTGGGTCGCCGAA
9-06-2-4fkatG         (1) ---------------------------------------------------
9-06-4fCter-katG      (1) ---------------------------------------------------
                                                                          Section 27
                   (1327) 1327         1340      1350      1360                1377
Msmeg-katG-coding  (1327) CCGCAGCTGTGGCAGGACCCGGTCCCGGCGGTCGATCATCCGCTGGTCGAC
9-06-2-4fkatG         (1) ---------------------------------------------------
9-06-4fCter-katG      (1) ---------------------------------------------------
                                                                          Section 28
                   (1378) 1378        1390      1400      1410                 1428
Msmeg-katG-coding  (1378) GACCAGGACATCGCCGCGCTGAAGTCCACGGTGCTCGATTCCGGGCTGTCG
9-06-2-4fkatG         (1) ---------------------------------------------------
9-06-4fCter-katG      (1) ---------------------------------------------------
```

```
                                                                     Section 29
                  (1429) 1429      1440      1450      1460              1479
Msmeg-katG-coding (1429) ACGGGACAACTGATCAAGACTGCGTGGGCGTCGGCGGCGAGCTACCGCAAC
9-06-2-4fkatG        (1) ---------------------------------------------------
9-06-4fCter-katG     (1) ---------------------------------------------------
                                                                     Section 30
                  (1480) 1480      1490      1500      1510      1520      1530
Msmeg-katG-coding (1480) ACCGACAAGCGCGGCGGCGCCAACGGGGCCCGGGTGCGTCTGGAACCGCAG
9-06-2-4fkatG        (1) ---------------------------------------------------
9-06-4fCter-katG     (1) ---------------------------------------------------
                                                                     Section 31
                  (1531) 1531     1540      1550      1560      1570       1581
Msmeg-katG-coding (1531) AAGAACTGGGACGTCAACGAACCGGCCGAACTGGCCACGGTGCTGCCGGTG
9-06-2-4fkatG        (1) ---------------------------------------------------
9-06-4fCter-katG     (1) ---------------------------------------------------
                                                                     Section 32
                  (1582) 1582    1590      1600      1610      1620        1632
Msmeg-katG-coding (1582) CTCGAGCGGATCCAGCAGGACTTCAACGCCTCGGCATCCGGCGGCAAGAAG
9-06-2-4fkatG        (1) ---------------------------------------------------
9-06-4fCter-katG     (1) ---------------------------------------------------
                                                                     Section 33
                  (1633) 1633   1640      1650      1660      1670         1683
Msmeg-katG-coding (1633) GTCTCGTTGGCCGACCTGATCGTGCTGGCCGGTTCGGCGGCGATCGAGAAG
9-06-2-4fkatG        (1) ---------------------------------------------------
9-06-4fCter-katG     (1) ---------------------------------------------------
                                                                     Section 34
                  (1684) 1684  1690      1700      1710      1720          1734
Msmeg-katG-coding (1684) GCCGCCAAGGACGGTGGCTACAACGTCACGGTGCCGTTCGCACCGGGACGC
9-06-2-4fkatG        (1) -------------------------------------------CWWWRYMC
9-06-4fCter-katG     (1) -------------------------------------------CCTRAMAM
                                                                     Section 35
                  (1735) 1735 1740      1750      1760      1770           1785
Msmeg-katG-coding (1735) ACGGACGCGAGCCAGGAGAACACCGACGTGGAGTCCTTCGCGGTGCTCGAG
9-06-2-4fkatG        (9) MGGGM-GCGRGCCAGGAGA-CACCGACGTGGAGTCCTTCGCGGTGCTCGAG
9-06-4fCter-katG     (9) A-RGM-GCGAGCCAGGAGA-CACCGACGTGGAGTCCTTCGCGGTGCTCGAG
```

Section 36

```
                  (1786) 1786         1800      1810      1820           1836
Msmeg-katG-coding (1786) CCGCGGGCCGACGGGTTCCGCAACTACGTCCGCCCGGGTGAGAAGGTCCAG
9-06-2-4fkatG       (58) CCGCGGGCCGACGGGTTCCGCAACTACGTCCGCCCGGGTGAGAAGGTCCAG
9-06-4fCter-katG    (57) CCGCGGGCCGACGGGTTCCGCAACTACGTCCGCCCGGGTGAGAAGGTCCAG
```

Section 37

```
                  (1837) 1837        1850      1860      1870            1887
Msmeg-katG-coding (1837) CTGGAGAAGATGCTGCTCGAACGGGCGTACTTCCTGGGCGTGACCGCACCG
9-06-2-4fkatG      (109) CTGGAGAAGATGCTGCTCGAACGGGCGTACTTCCTGGGCGTGACCGCACCG
9-06-4fCter-katG   (108) CTGGAGAAGATGCTGCTCGAACGGGCGTACTTCCTGGGCGTGACCGCACCG
```

Section 38

```
                  (1888) 1888       1900      1910      1920             1938
Msmeg-katG-coding (1888) CAGCTGACGGCTCTGGTCGGCGGGTTGCGCGCGCTGGACGTCAACCACGGC
9-06-2-4fkatG      (160) CAGCTGACGGCTCTGGTCGGCGGGTTGCGCGCGCTGGACGTCAACCACGGC
9-06-4fCter-katG   (159) CAGCTGACGGCTCTGGTCGGCGGGTTGCGCGCGCTGGACGTCAACCACGGC
```

Section 39

```
                  (1939) 1939      1950      1960      1970              1989
Msmeg-katG-coding (1939) GGCACCAAACACGGTGTGTTCACCGACCGGCCGGGCGCTTTGACCAACGAC
9-06-2-4fkatG      (211) GGCACCAAACACGGTGTGTTCACCGACCGGCCGGGCGCTTTGACCAACGAC
9-06-4fCter-katG   (210) GGCACCAAACACGGTGTGTTCACCGACCGGCCGGGCGCTTTGACCAACGAC
```

Section 40

```
                  (1990) 1990     2000      2010      2020      2030     2040
Msmeg-katG-coding (1990) TTCTTCGTCAACCTGCTCGACATGGGCACCGAGTGGAAGACCTCGGAGACG
9-06-2-4fkatG      (262) TTCTTCGTCAACCTGCTCGACATGGGCACCGAGTGGAAGACCTCGGAGACG
9-06-4fCter-katG   (261) TTCTTCGTCAACCTGCTCGACATGGGCACCGAGTGGAAGACCTCGGAGACG
```

Section 41

```
                  (2041) 2041     2050      2060      2070      2080     2091
Msmeg-katG-coding (2041) ACGGAGAACGTCTACGAGGGAGTCGATCGCAAGACCGGACAGTTGAAGTGG
9-06-2-4fkatG      (313) ACGGAGAACGTCTACGAGGGAGTCGATCGCAAGACCGGACAGTTGAAGTGG
9-06-4fCter-katG   (312) ACGGAGAACGTCTACGAGGGAGTCGATCGCAAGACCGGACAGTTGAAGTGG
```

Section 42

```
                  (2092) 2092    2100      2110      2120      2130      2142
Msmeg-katG-coding (2092) ACCGCGACCGCGAATGACCTTGTGTTCGGGTCACATTCGGTGCTGCGTGCC
9-06-2-4fkatG      (364) ACCGCGACCGCGAATGACCTTGTGTTCGGGTCACATTCGGTGCTGCGTGCC
9-06-4fCter-katG   (363) ACCGCGACCGCGAATGACCTTGTGTTCGGGTCACATTCGGTGCTGCGTGCC
```

Section 43

```
                    (2143) 2143   2150      2160      2170      2180          2193
Msmeg-katG-coding   (2143) GTGGCCGAGGTTTATGCCCAGTCCGACAACGGCGAACGGTTCGTCAACGAC
9-06-2-4fkatG        (415) GTGGCCGAGGTTTATGCCCAGTCCGACAACGGCGAACGGTTCGTCAACGAC
9-06-4fCter-katG     (414) GTGGCCGAGGTTTATGCCCAGTCCGACAACGGCGAACGGTTCGTCAACGAC
```

Section 44

```
                    (2194) 2194  2200      2210      2220      2230           2244
Msmeg-katG-coding   (2194) TTCGTCAAGGCCTGGGTCAAGGTCATGAACAACGACCGGTTCGACCTCAAG
9-06-2-4fkatG        (466) TTCGTCAAGGCCTGGGTCAAGGTCATGAACAACGACCGGTTCGACCTCAAG
9-06-4fCter-katG     (465) TTCGTCAAGGCCTGGGTCAAGGTCATGAACAACGACCGGTTCGACCTCAAG
```

Section 45

```
                    (2245) 2245 2248
Msmeg-katG-coding   (2245) TAA-
9-06-2-4fkatG        (517) TAAA
9-06-4fCter-katG     (516) TAAA
```
